# Supplementary material for: Multiple polarity kinases inhibit phase separation of F-BAR protein Cdc15 and antagonize cytokinetic ring assembly in fission yeast
Source: eLife. 2023 Feb 7;12:e83062. doi: 10.7554/eLife.83062 (PMC9904764; doi:10.7554/eLife.83062)

Figure 1A

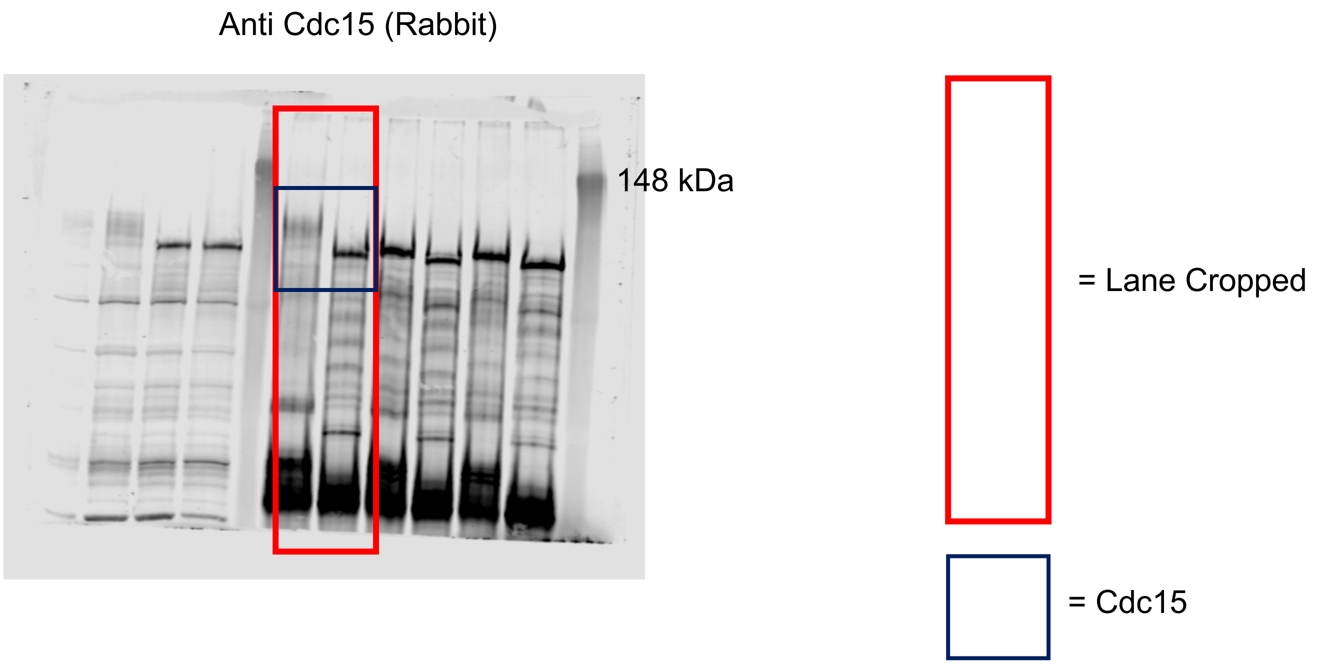

Figure 1B

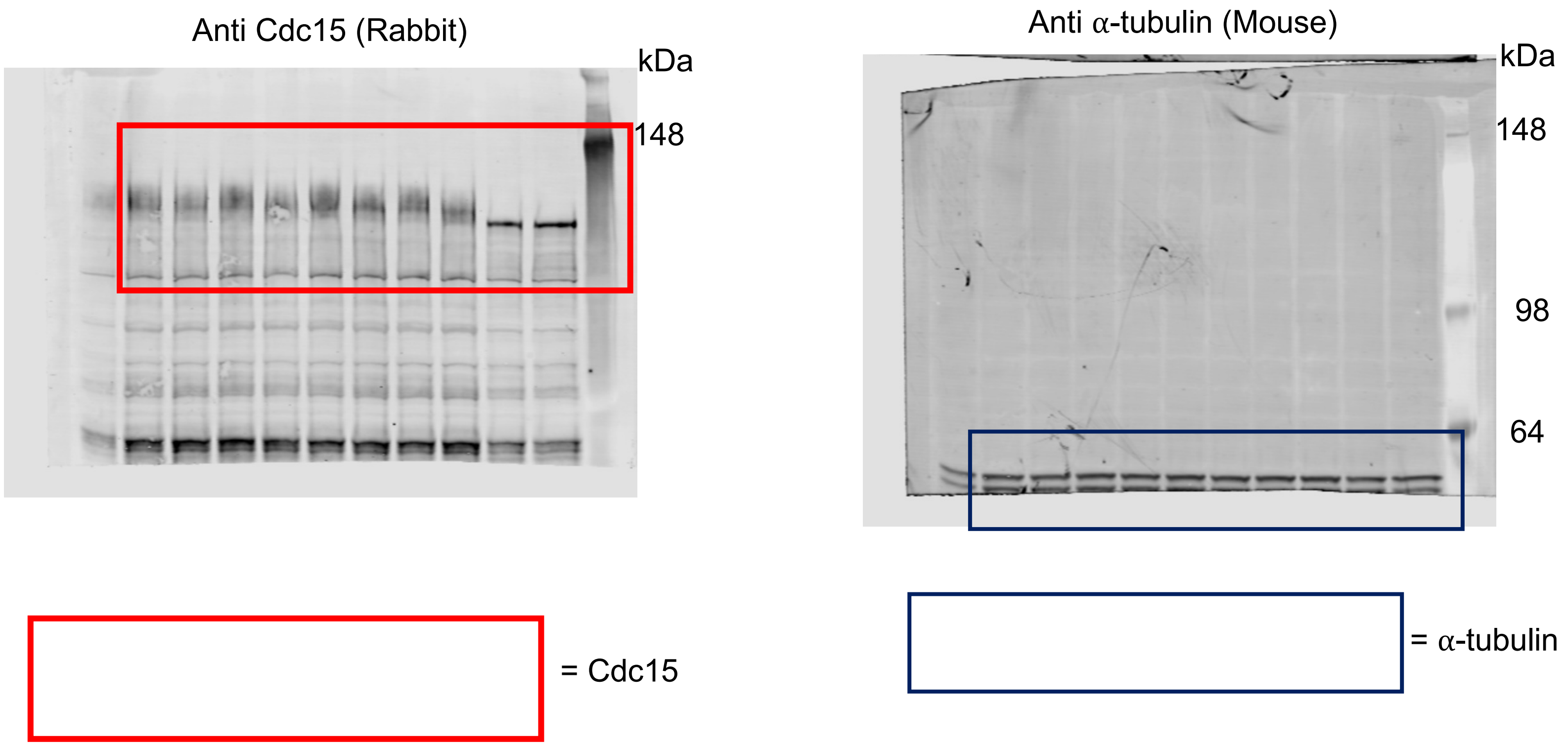
Anti Cdc15 (Rabbit)
kDa
148
= Cdc15
Anti α-tubulin (Mouse)
kDa
148
98
64
= α-tubulin

Figure 1C

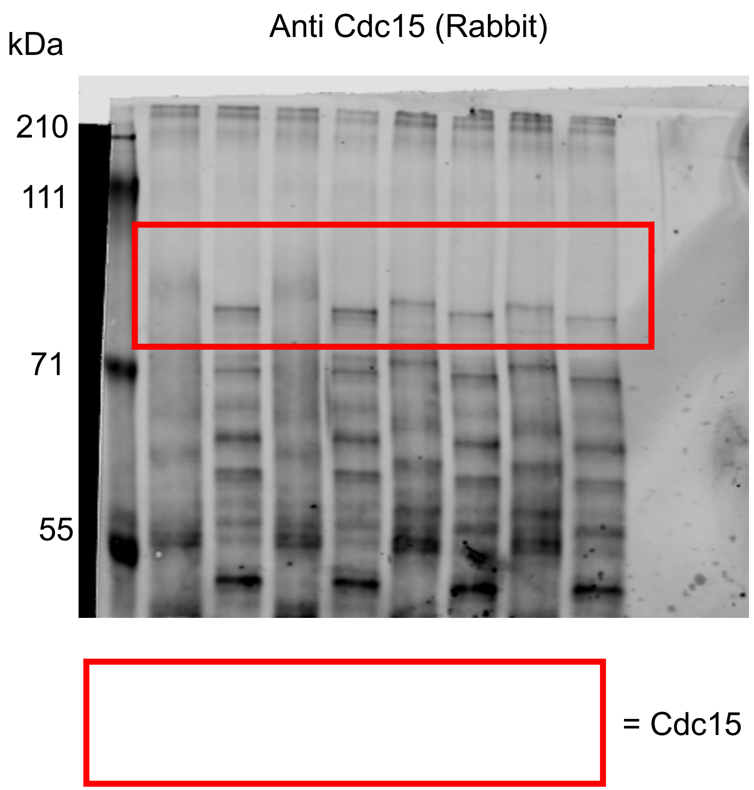

Figure 1D

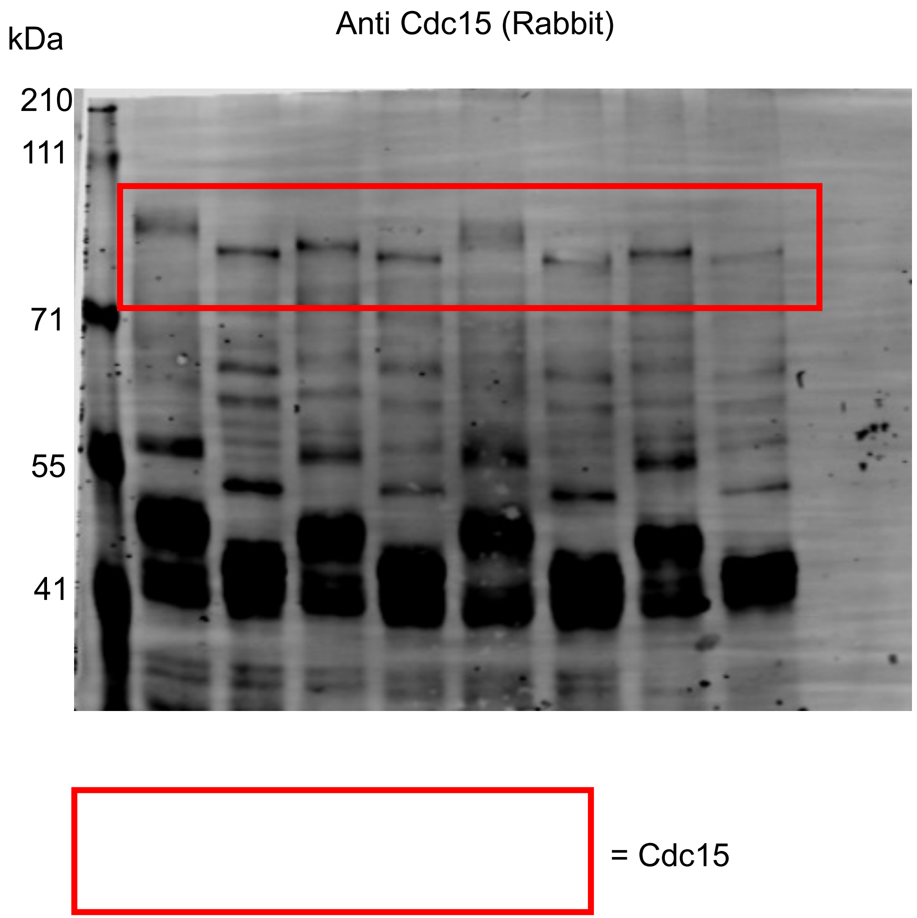

Figure 1E

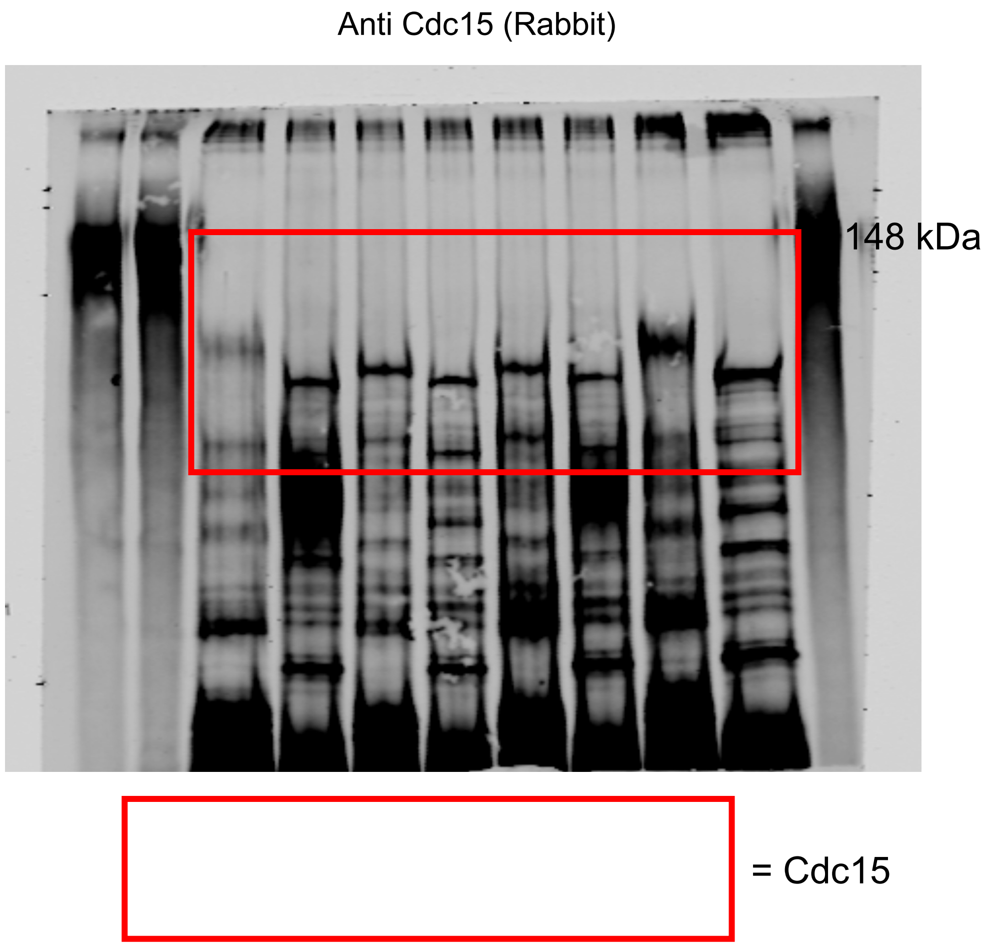

Figure 1F

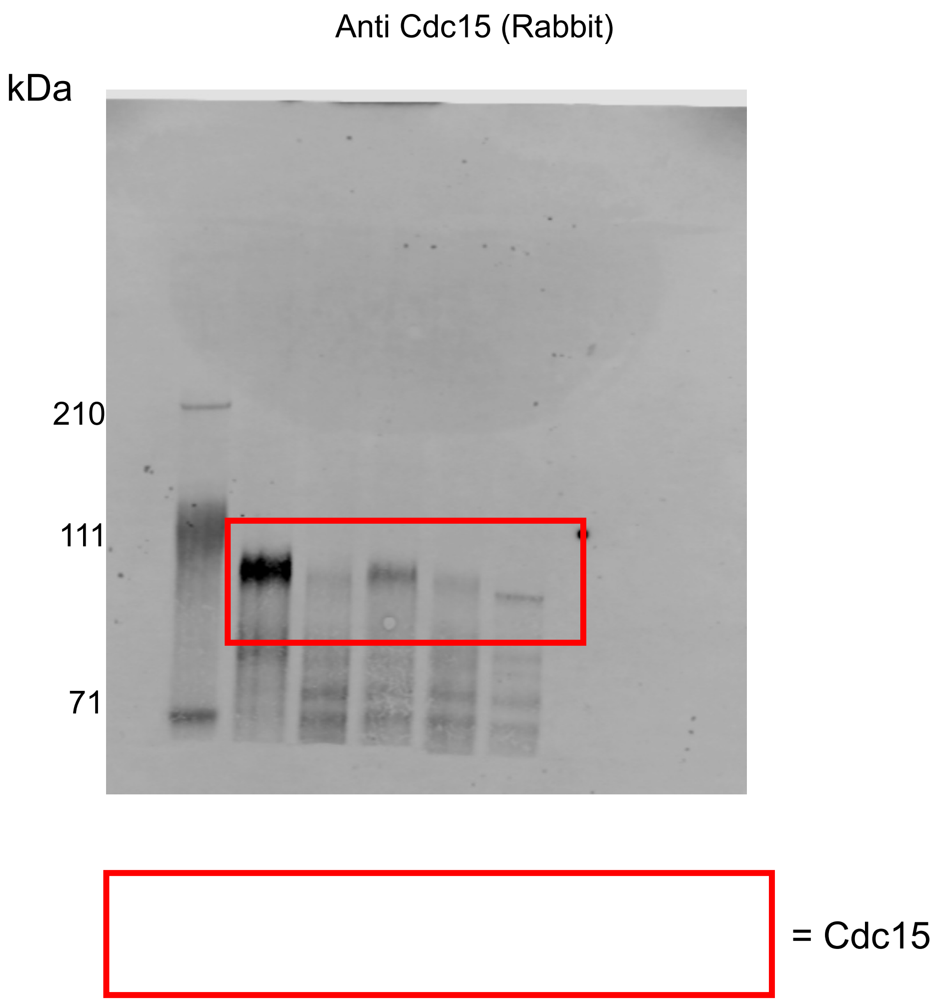

Figure 1G

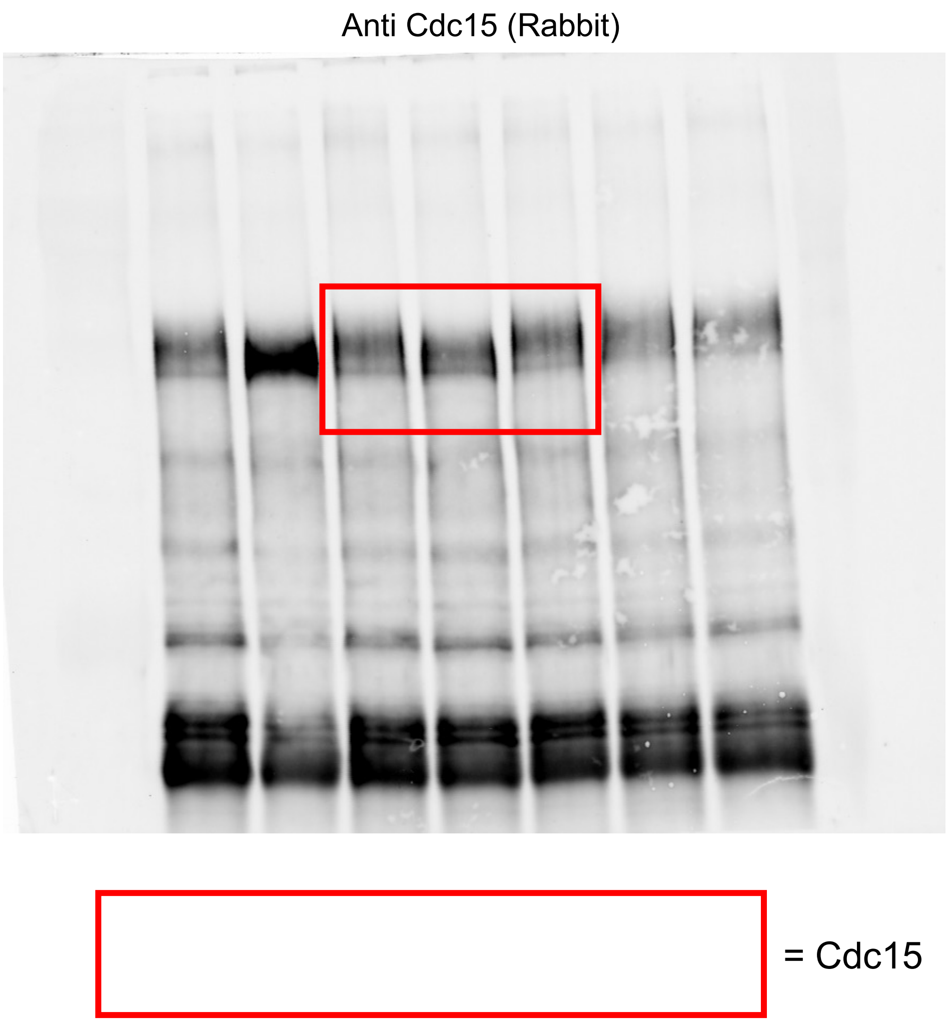

Figure 1H

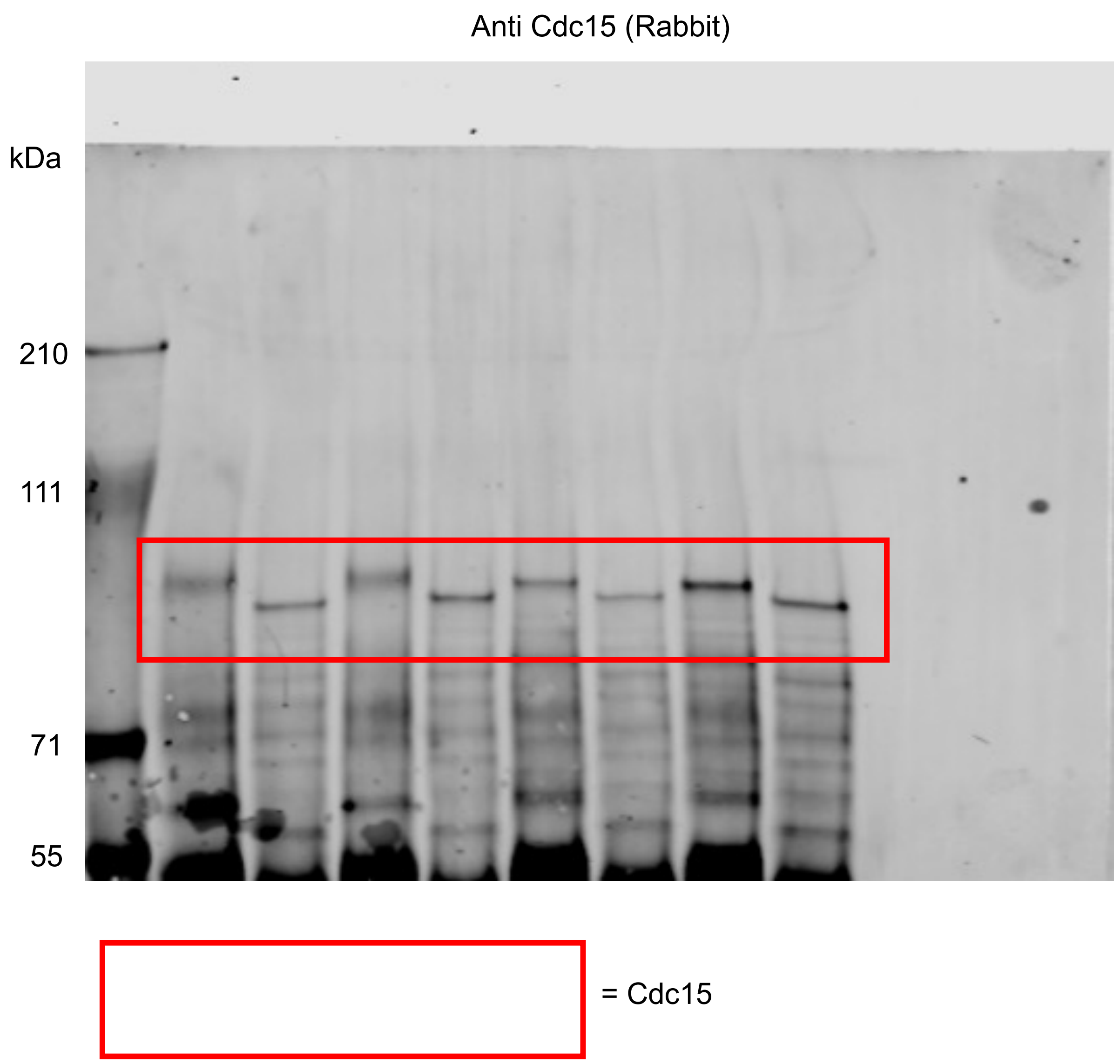

Figure 1I

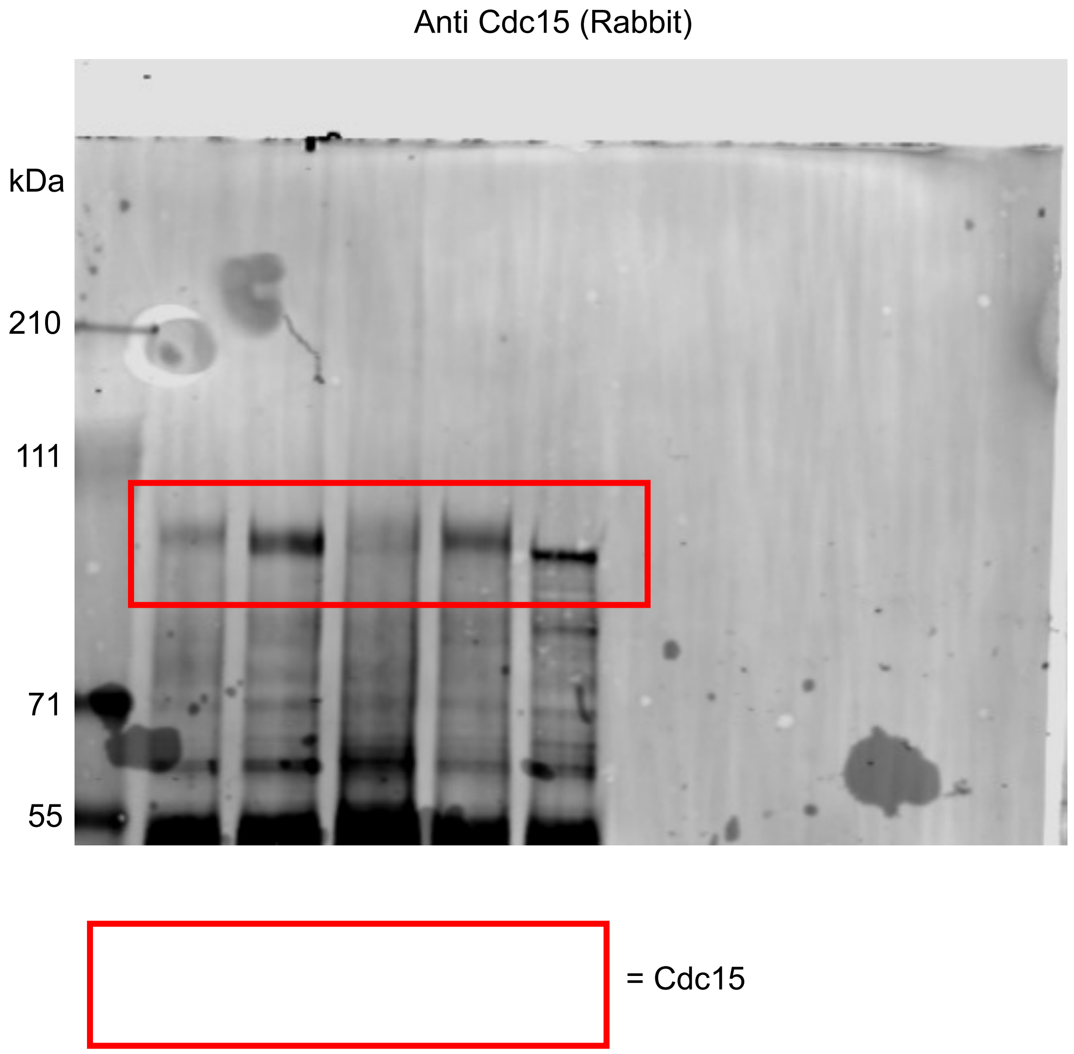

Supplement: Figure 1—source data 1. [file elife-83062-fig1-data1.zip › Figure 1/Figure 1-Source file labeled.pdf]
