# Supplementary figures and images for: Multiple polarity kinases inhibit phase separation of F-BAR protein Cdc15 and antagonize cytokinetic ring assembly in fission yeast
Source: eLife. 2023 Feb 7;12:e83062. doi: 10.7554/eLife.83062 (PMC9904764; doi:10.7554/eLife.83062)

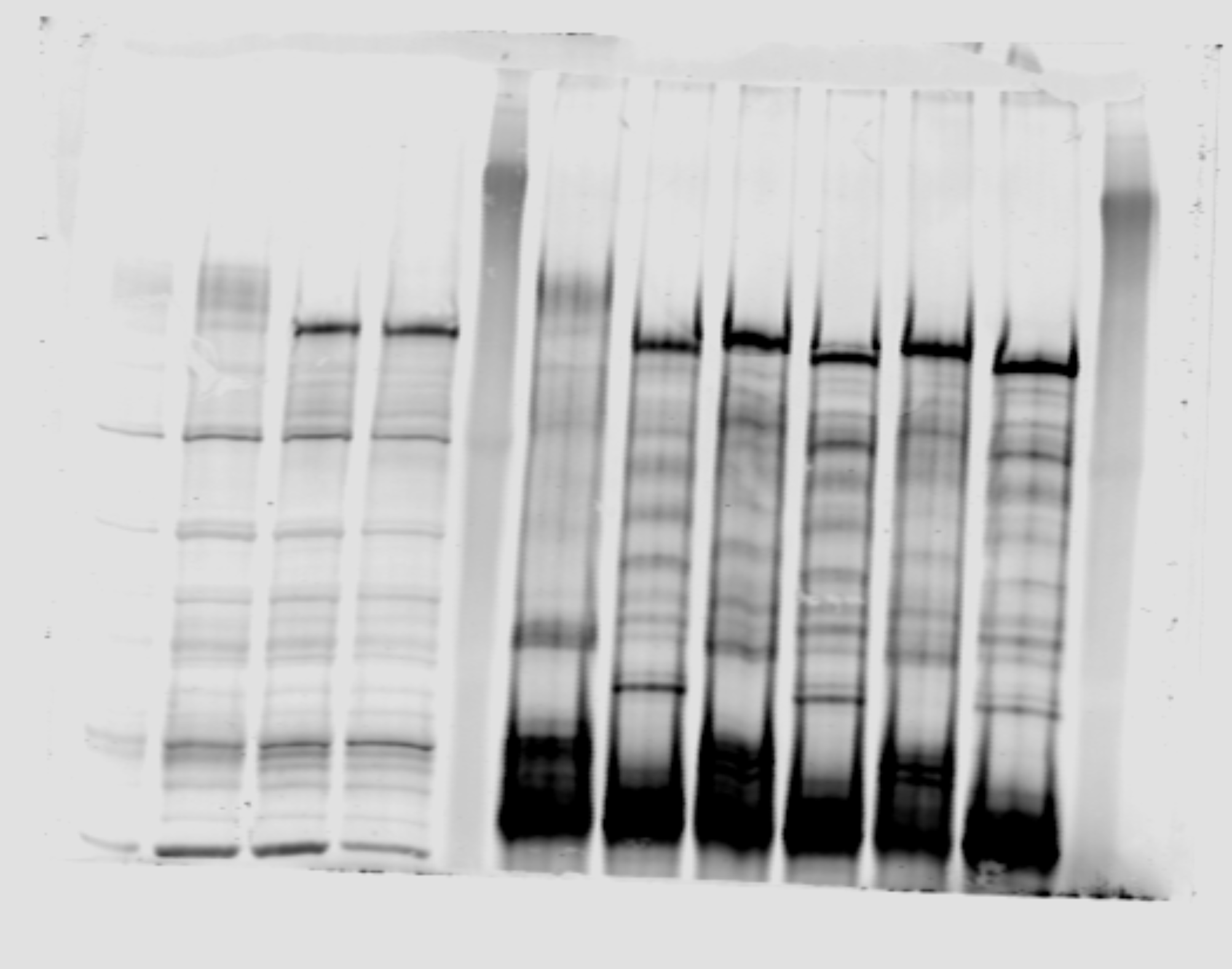

Supplement: Figure 1—source data 1. [file elife-83062-fig1-data1.zip › Figure 1/1A-anti Cdc15.tif]

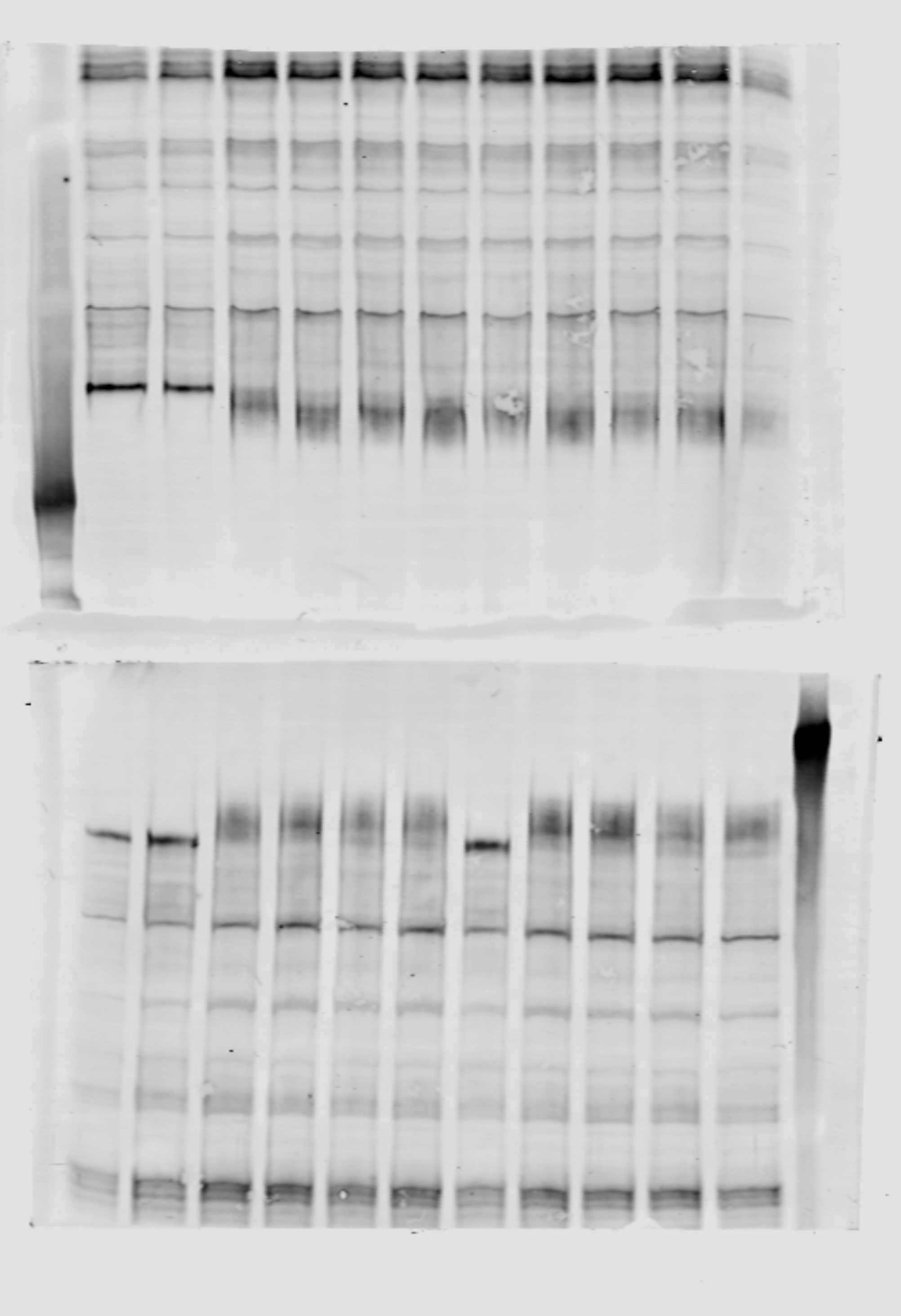

Supplement: Figure 1—source data 1. [file elife-83062-fig1-data1.zip › Figure 1/1B-anti Cdc15.tif]

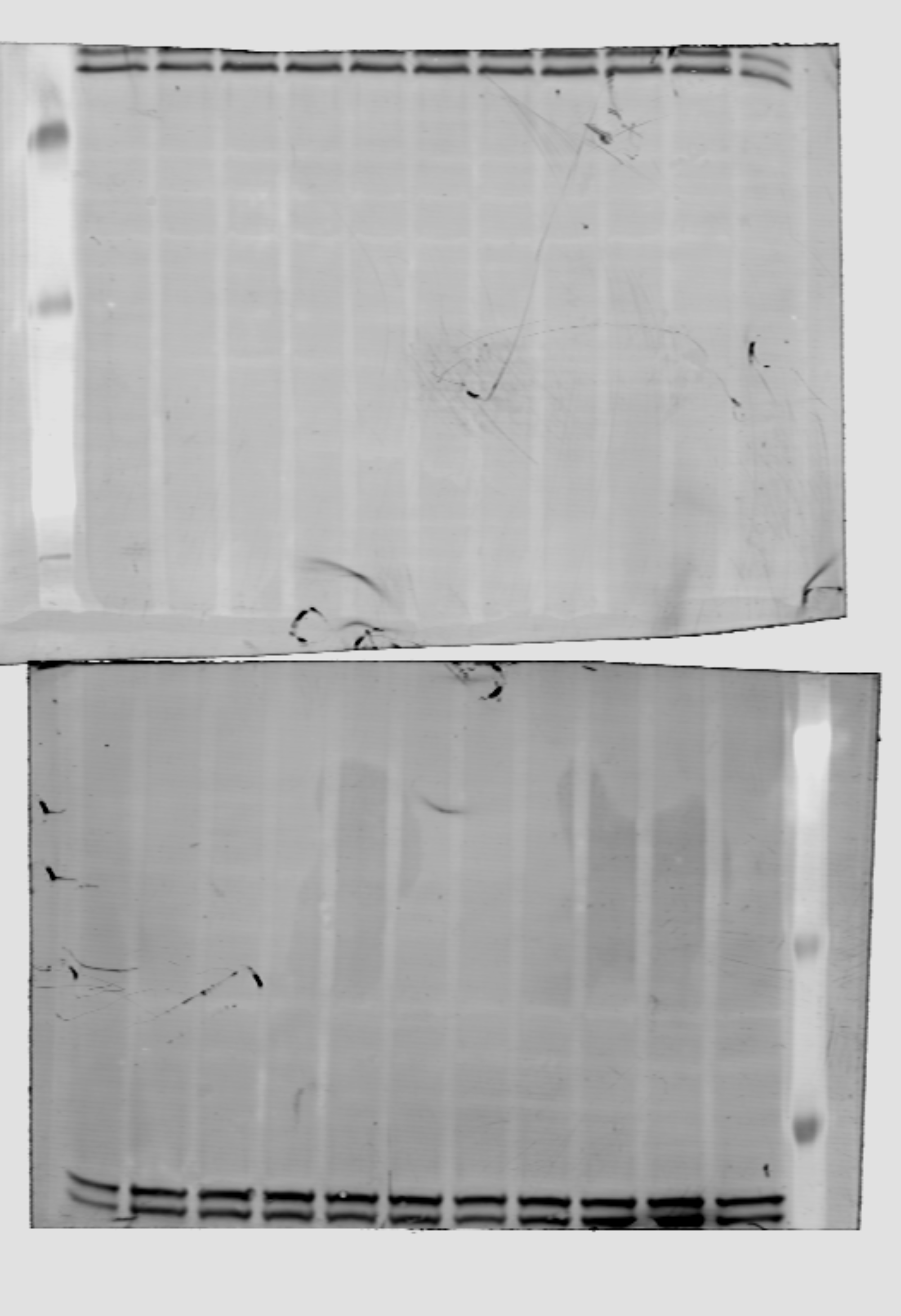

Supplement: Figure 1—source data 1. [file elife-83062-fig1-data1.zip › Figure 1/1B-anti tubulin.tif]

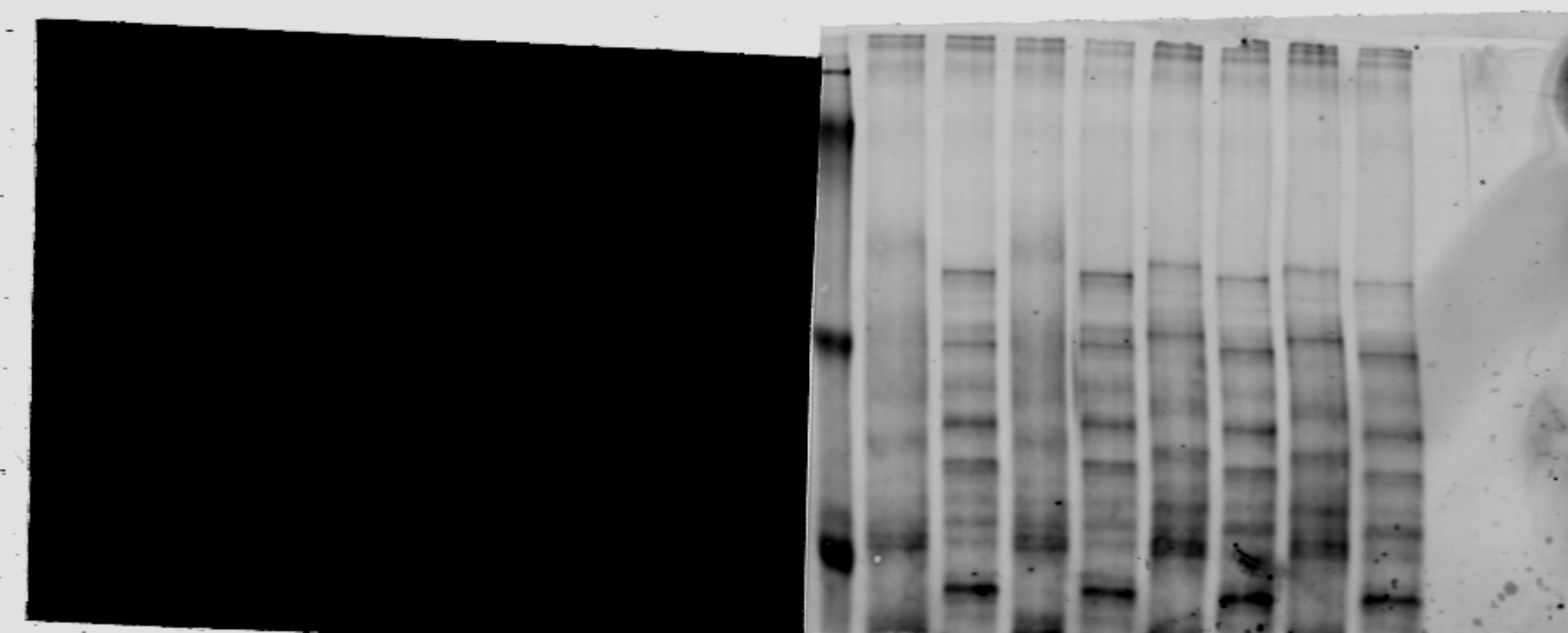

Supplement: Figure 1—source data 1. [file elife-83062-fig1-data1.zip › Figure 1/1C-anti Cdc15.tif]

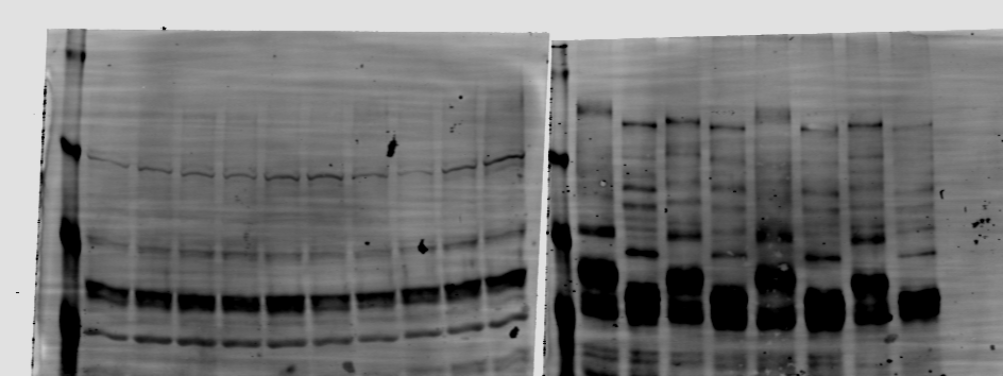

Supplement: Figure 1—source data 1. [file elife-83062-fig1-data1.zip › Figure 1/1D-anti Cdc15.tif]

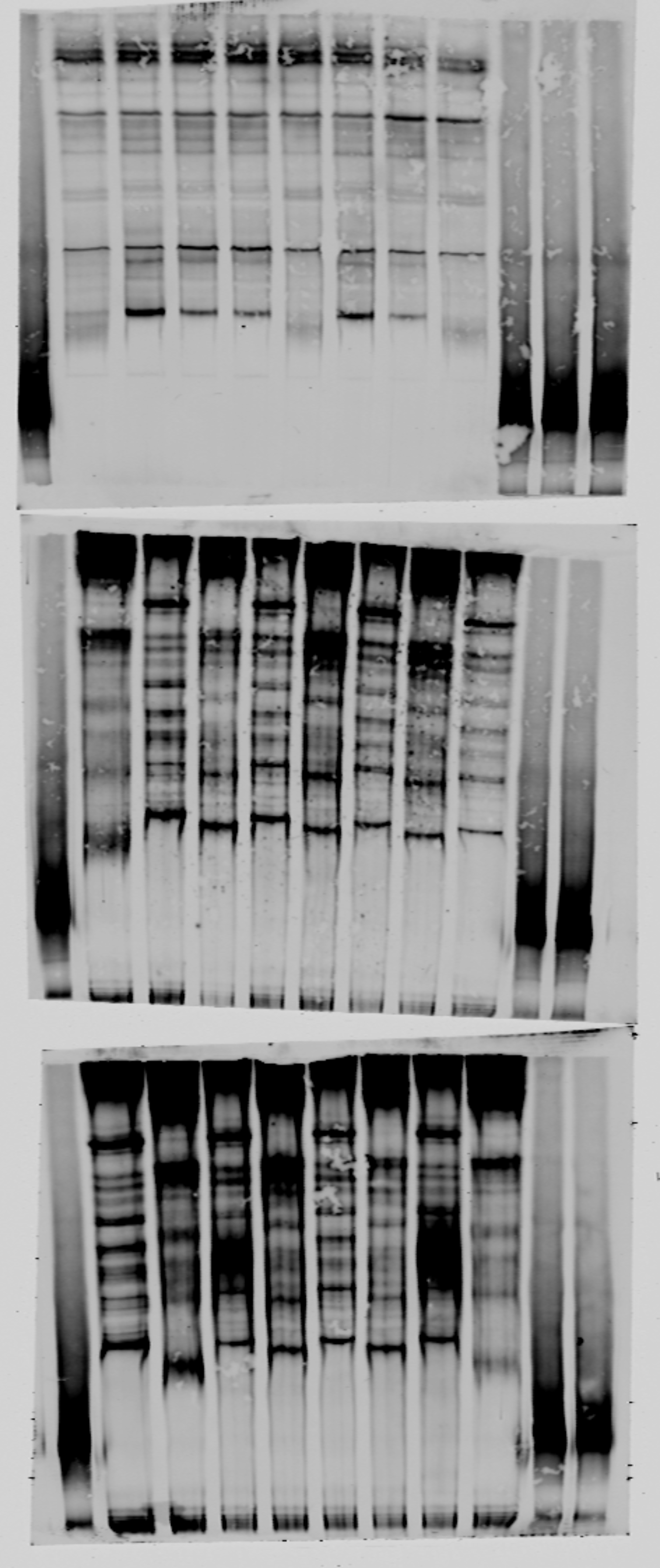

Supplement: Figure 1—source data 1. [file elife-83062-fig1-data1.zip › Figure 1/1E-anti Cdc15.tif]

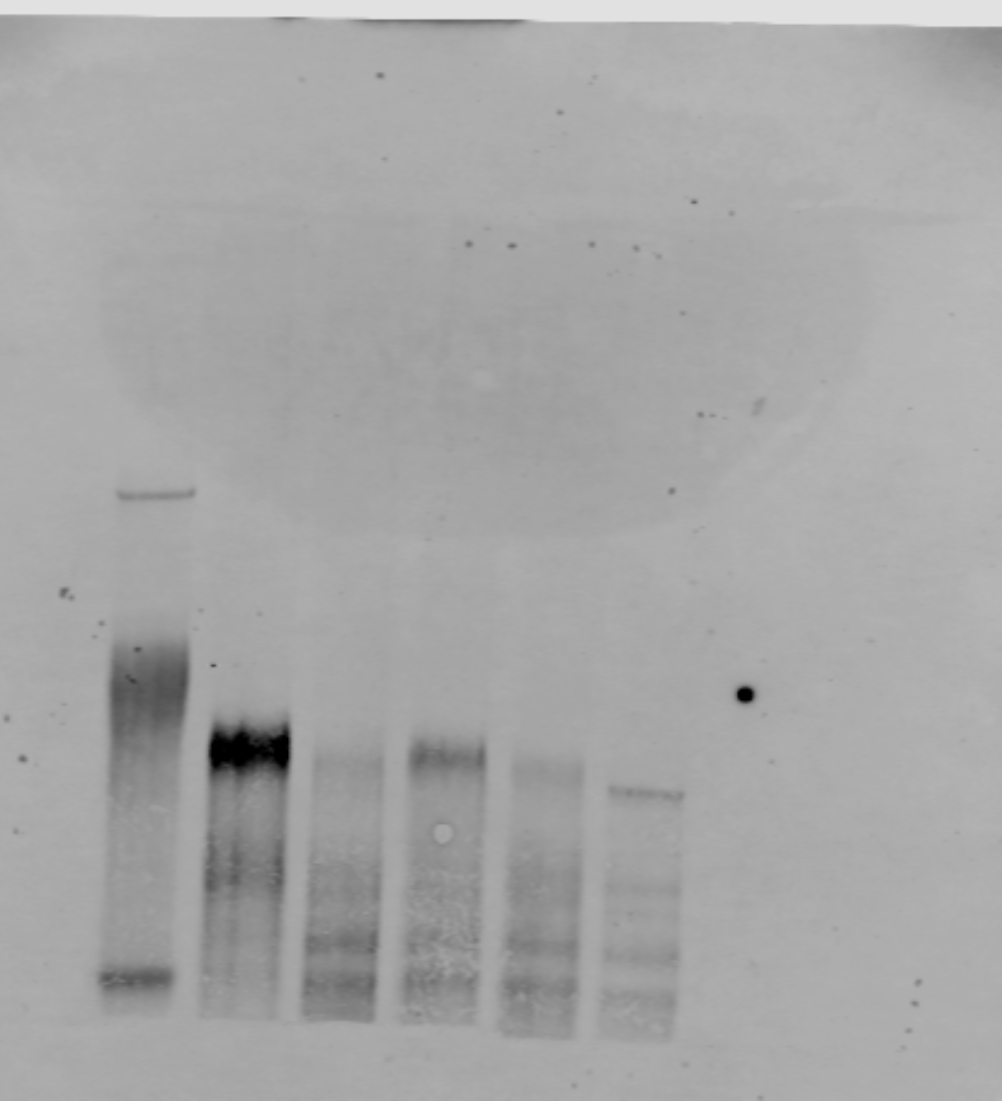

Supplement: Figure 1—source data 1. [file elife-83062-fig1-data1.zip › Figure 1/1F-anti Cdc15.tif]

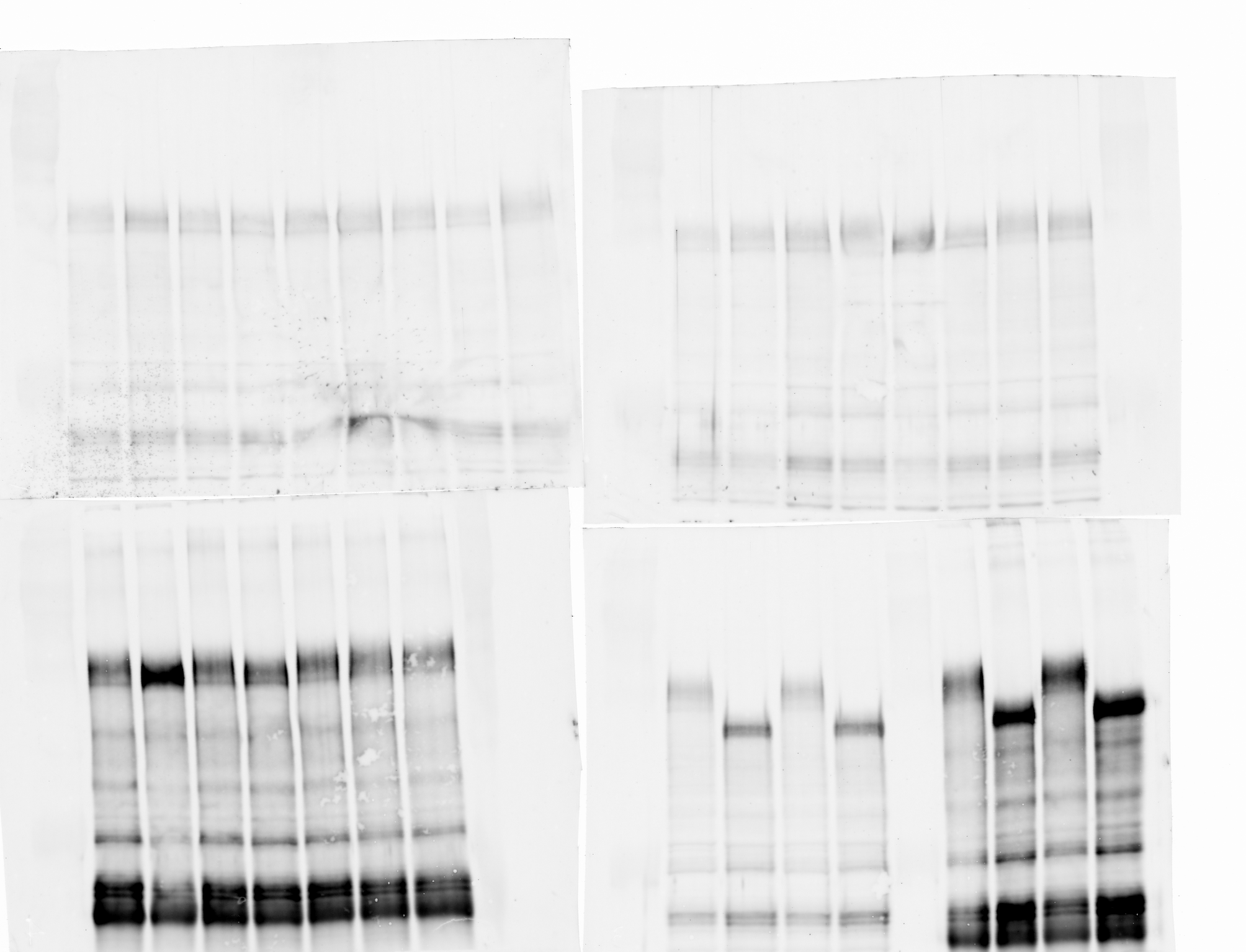

Supplement: Figure 1—source data 1. [file elife-83062-fig1-data1.zip › Figure 1/1G-anti Cdc15 (Bottom left).tif]

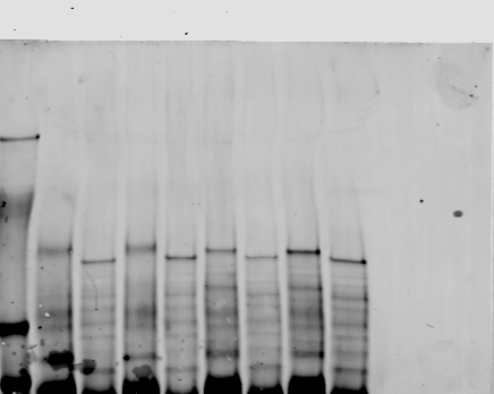

Supplement: Figure 1—source data 1. [file elife-83062-fig1-data1.zip › Figure 1/1H-anti Cdc15.tif]

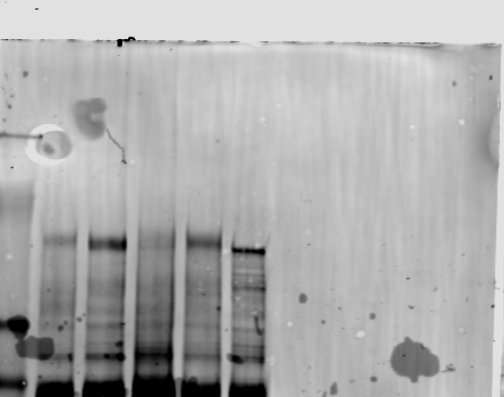

Supplement: Figure 1—source data 1. [file elife-83062-fig1-data1.zip › Figure 1/1I-anti Cdc15.tif]

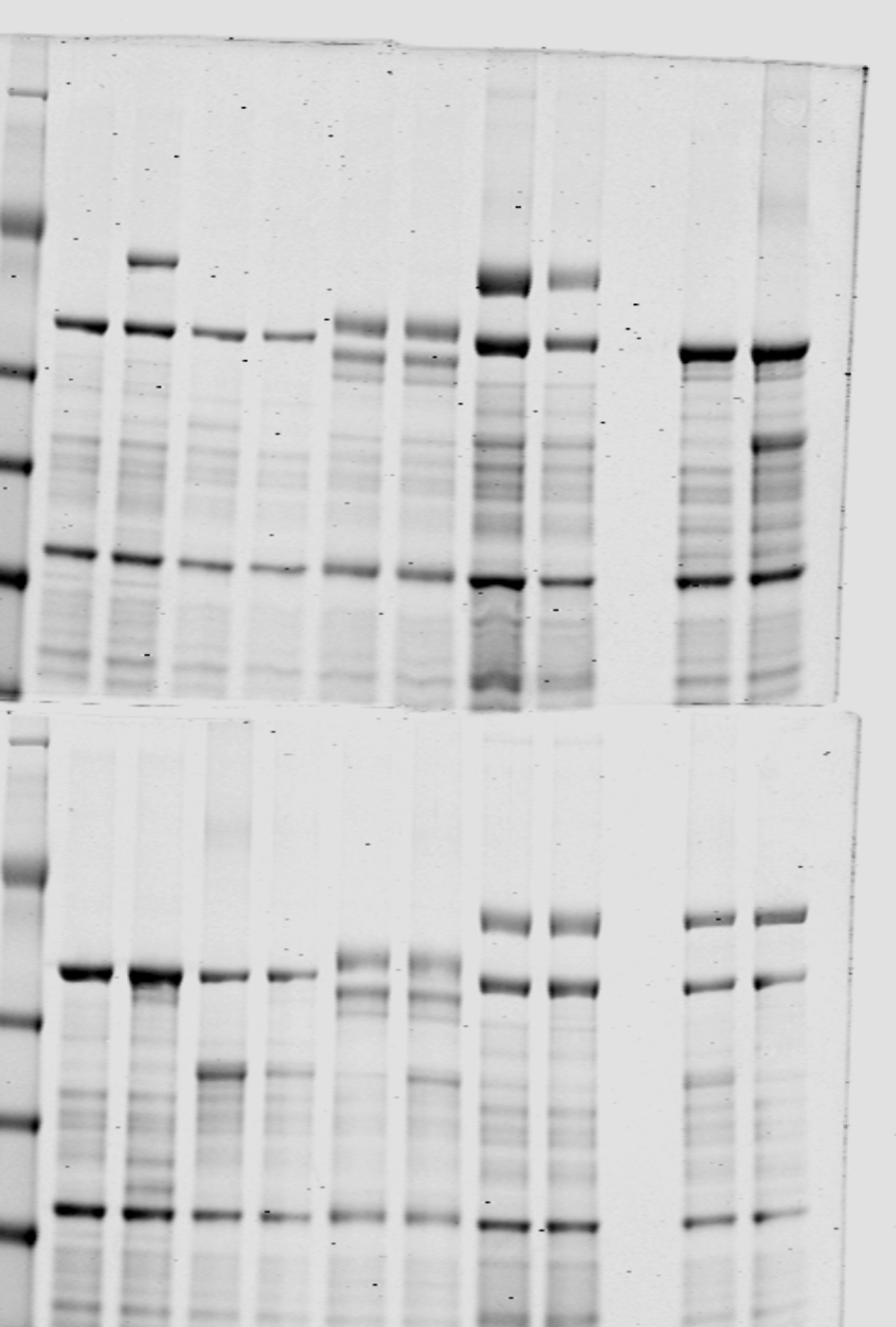

Supplement: Figure 2—source data 1. [file elife-83062-fig2-data1.zip › Figure 2-new source files/2C (top)_2D (bottom)-Coomassie stain.tif]

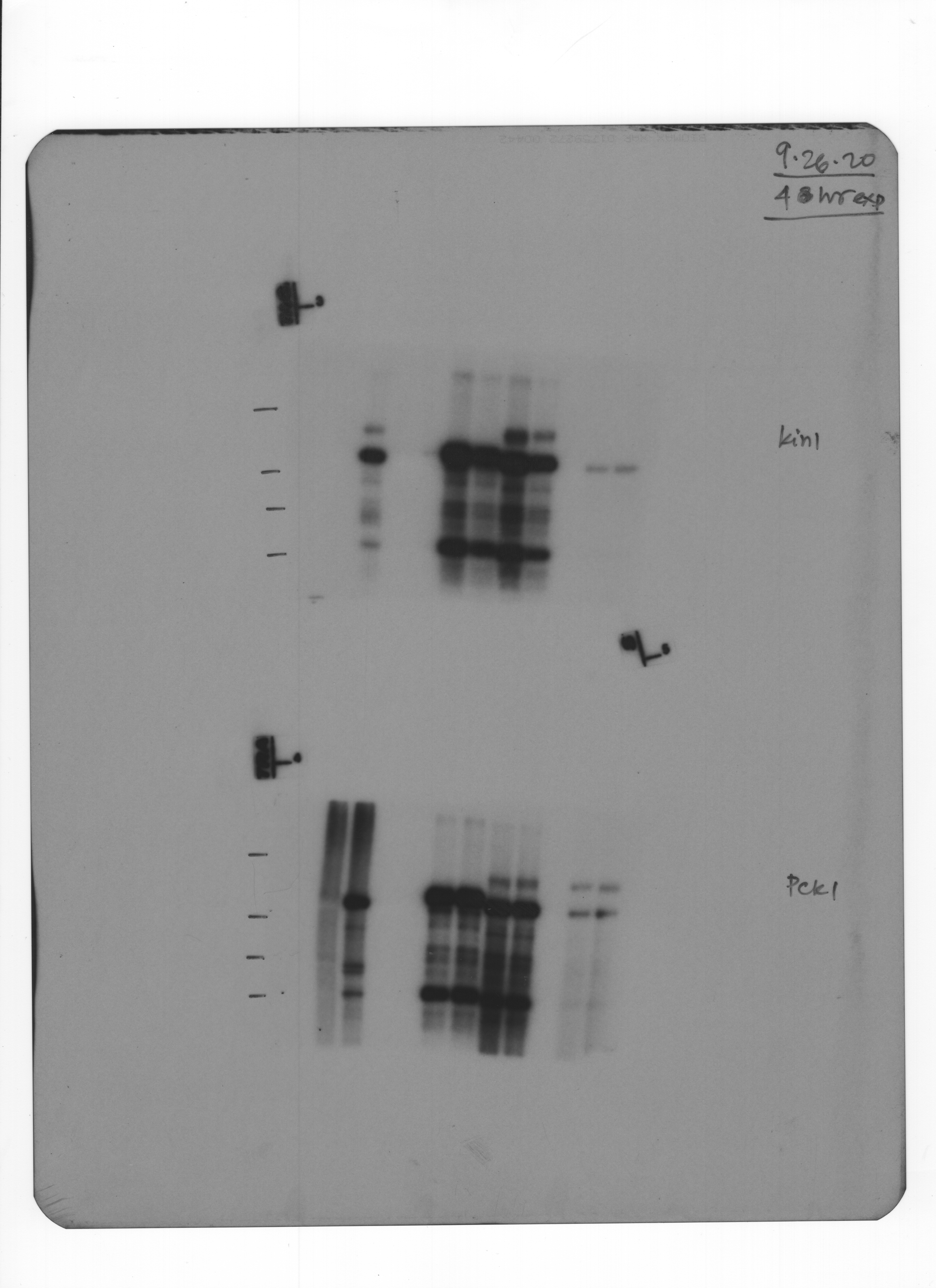

Supplement: Figure 2—source data 1. [file elife-83062-fig2-data1.zip › Figure 2-new source files/2C (top)_2D (bottom)-longer exposure-p32.tif]

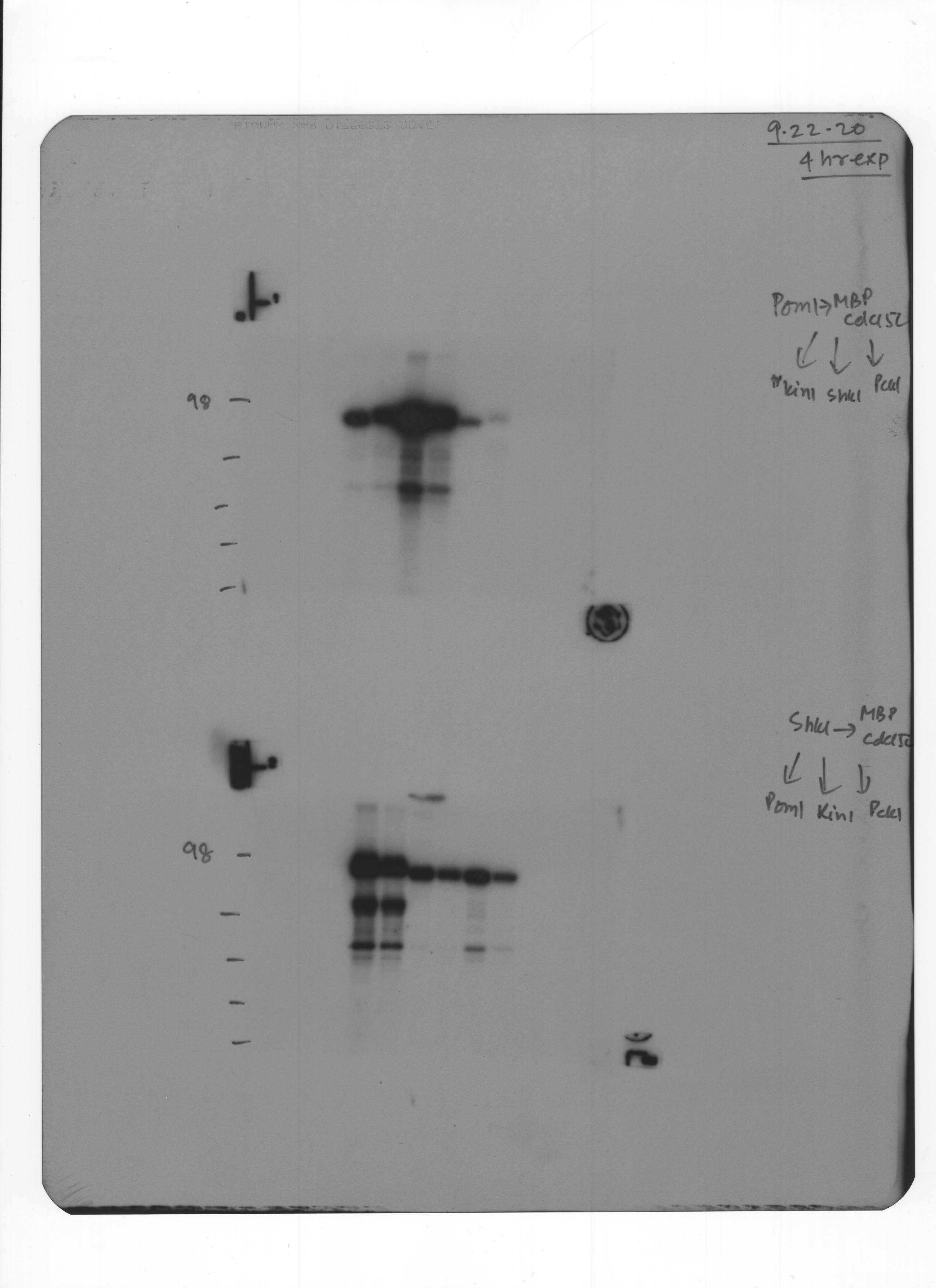

Supplement: Figure 2—source data 1. [file elife-83062-fig2-data1.zip › Figure 2-new source files/2A (top)_2B (bottom)-p32-longer exposure.tif]

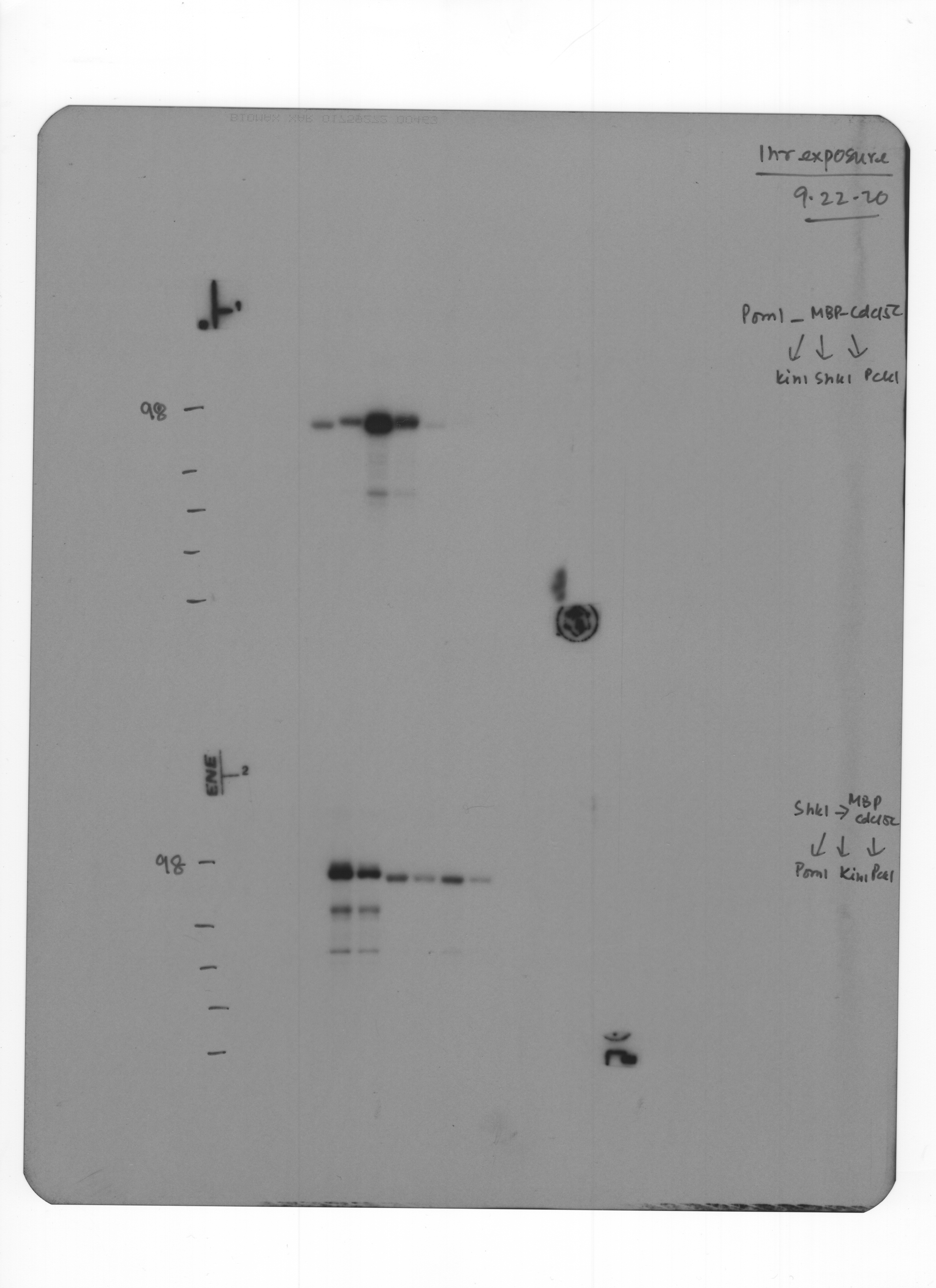

Supplement: Figure 2—source data 1. [file elife-83062-fig2-data1.zip › Figure 2-new source files/2A (top)_2B (bottom)-p32-short exposure.tif]

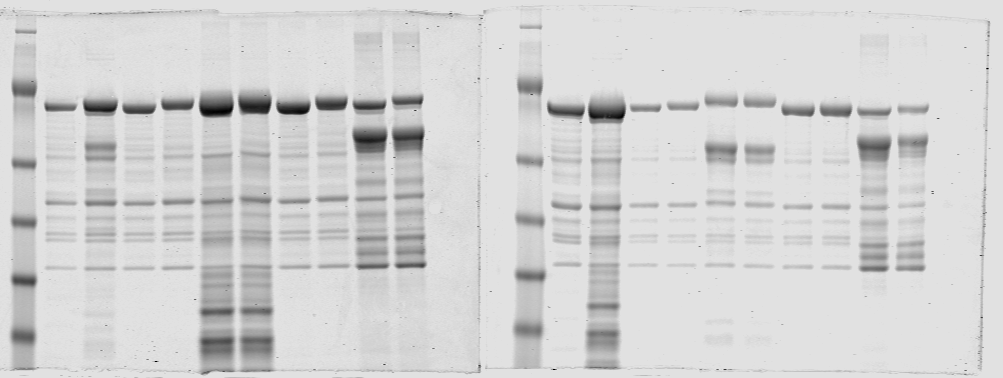

Supplement: Figure 2—source data 1. [file elife-83062-fig2-data1.zip › Figure 2-new source files/2A (left)-Coomassie stain.tif]

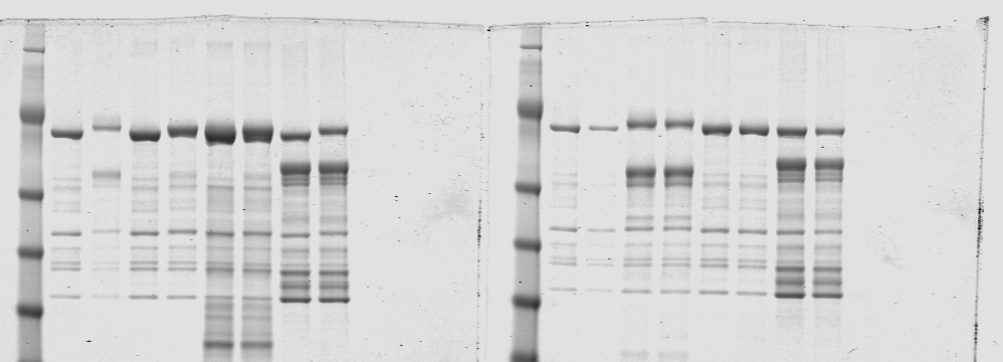

Supplement: Figure 2—source data 1. [file elife-83062-fig2-data1.zip › Figure 2-new source files/2B (right)-Commassie stain.tif]

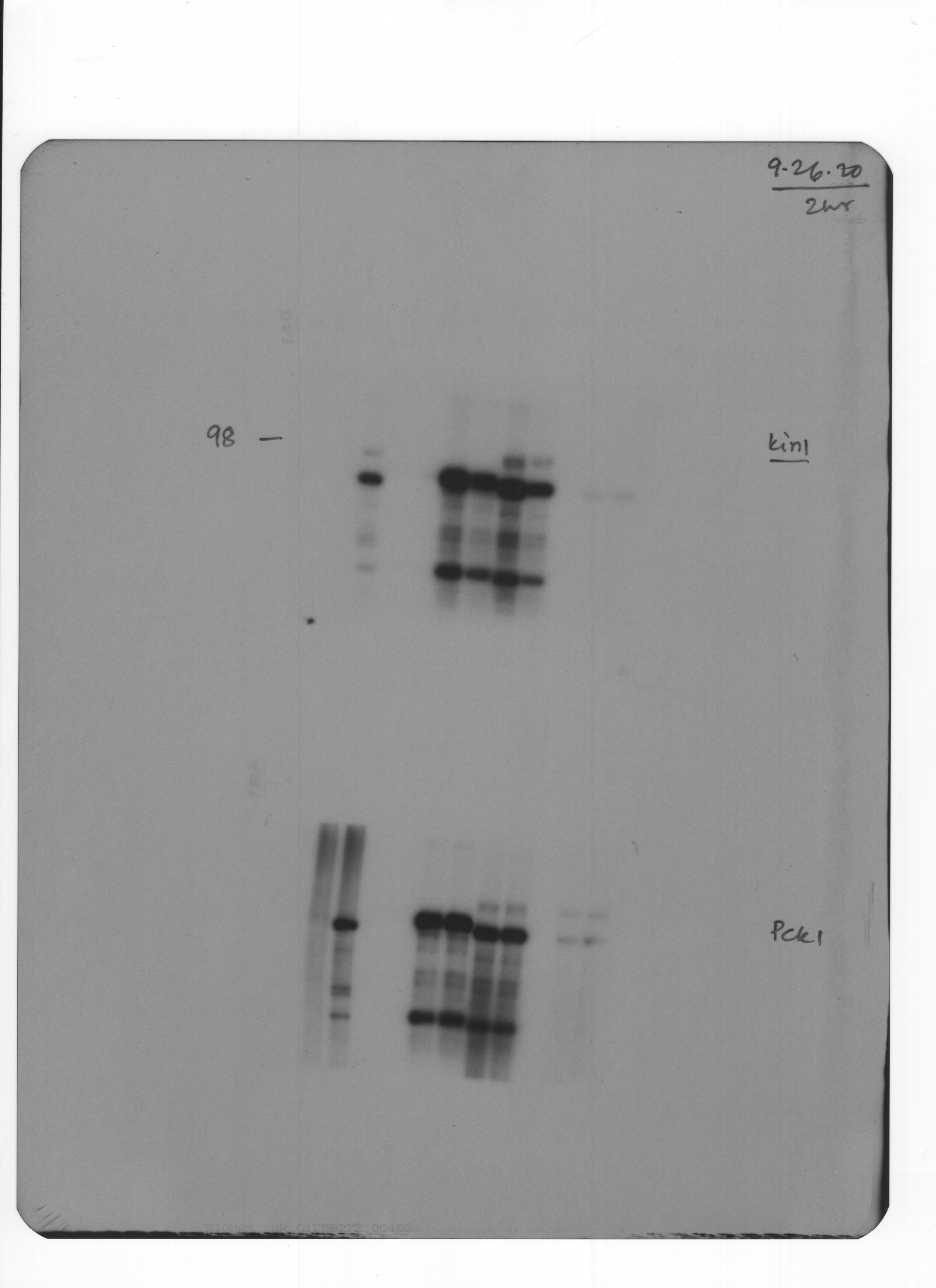

Supplement: Figure 2—source data 1. [file elife-83062-fig2-data1.zip › Figure 2-new source files/2C (top)_2D (bottom)-short exposure-p32.tif]

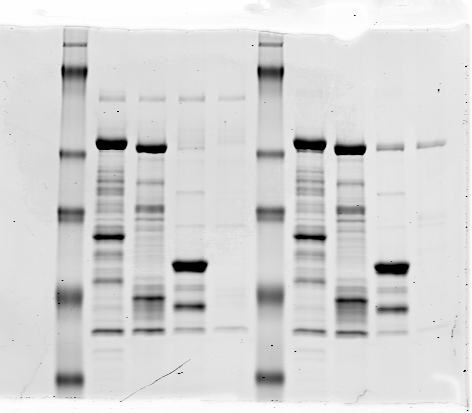

Supplement: Figure 2—figure supplement 1—source data 1. [file elife-83062-fig2-figsupp1-data1.zip › Figure 1-figure supplement 1/Figure 1-supplemen 1A (Kin1)/Kin1_Coomassie stain.tif]

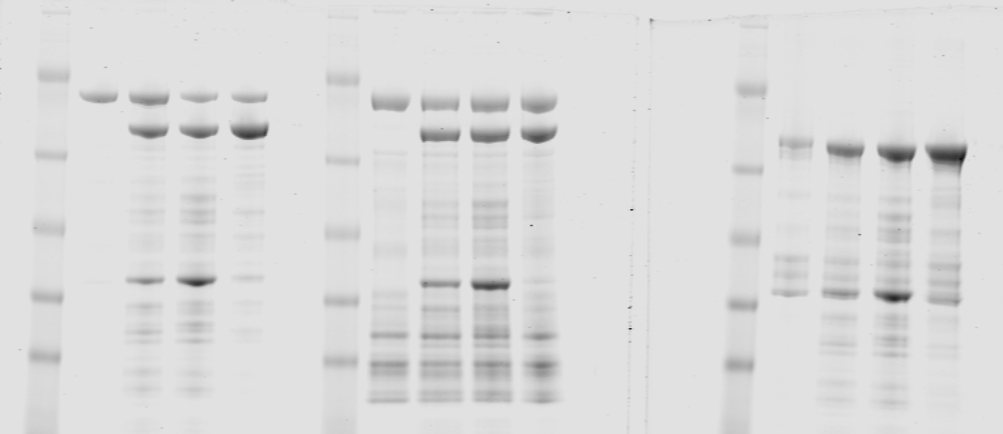

Supplement: Figure 2—figure supplement 1—source data 1. [file elife-83062-fig2-figsupp1-data1.zip › Figure 1-figure supplement 1/Figure 1-supplement 1B/Kin1 (left)_Shk1 (middle)_Pck1 (right)-Coomassie stain.tif]

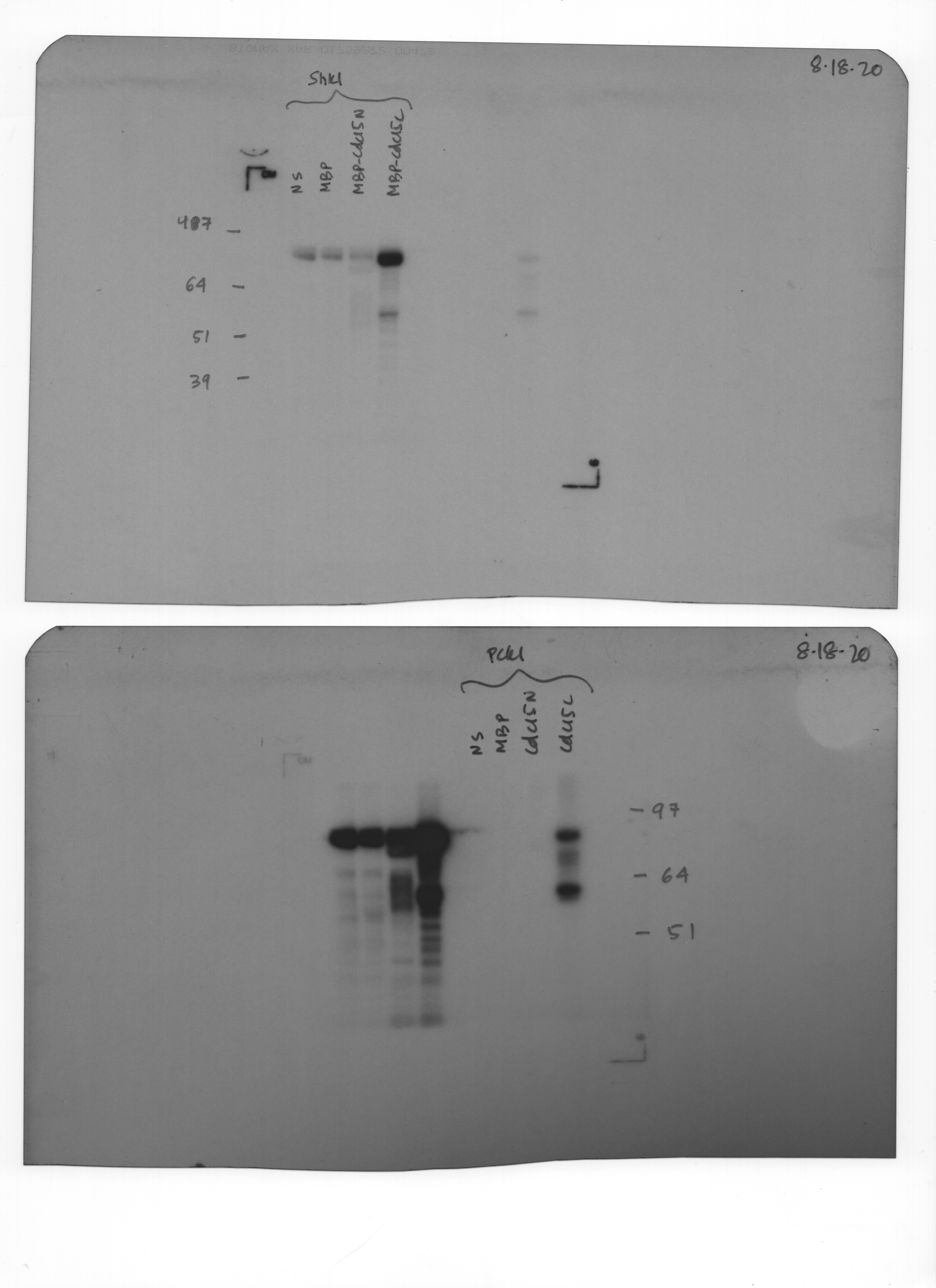

Supplement: Figure 2—figure supplement 1—source data 1. [file elife-83062-fig2-figsupp1-data1.zip › Figure 1-figure supplement 1/Figure 1-supplement 1A (Shk1 and Pck1)/1A-Shk1 (top)_Pck1 (bottom)-p32.tif]

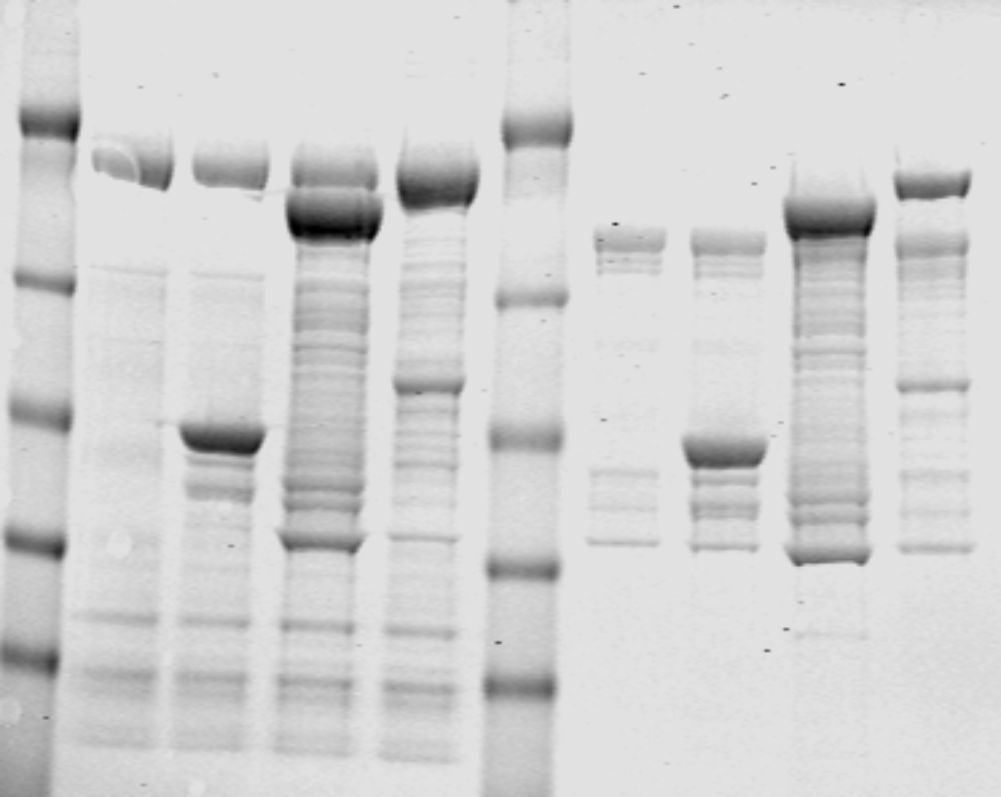

Supplement: Figure 2—figure supplement 1—source data 1. [file elife-83062-fig2-figsupp1-data1.zip › Figure 1-figure supplement 1/Figure 1-supplement 1A (Shk1 and Pck1)/1A-Shk1(Left)_Pck1 (right)-Coomassie stain.tif]

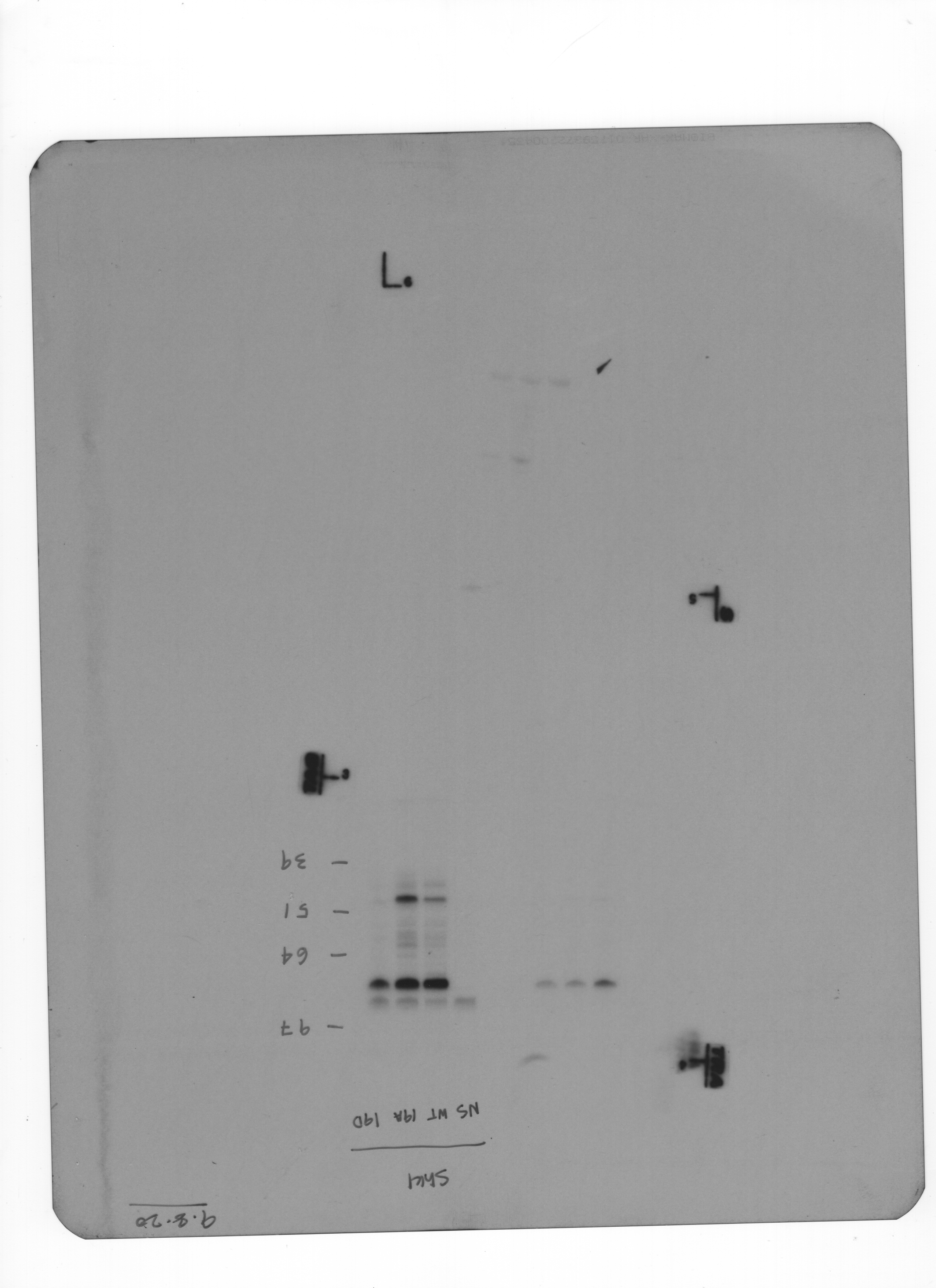

Supplement: Figure 2—figure supplement 1—source data 1. [file elife-83062-fig2-figsupp1-data1.zip › Figure 1-figure supplement 1/Figure 1-supplement 1B/p32/shk1-32p.tif]

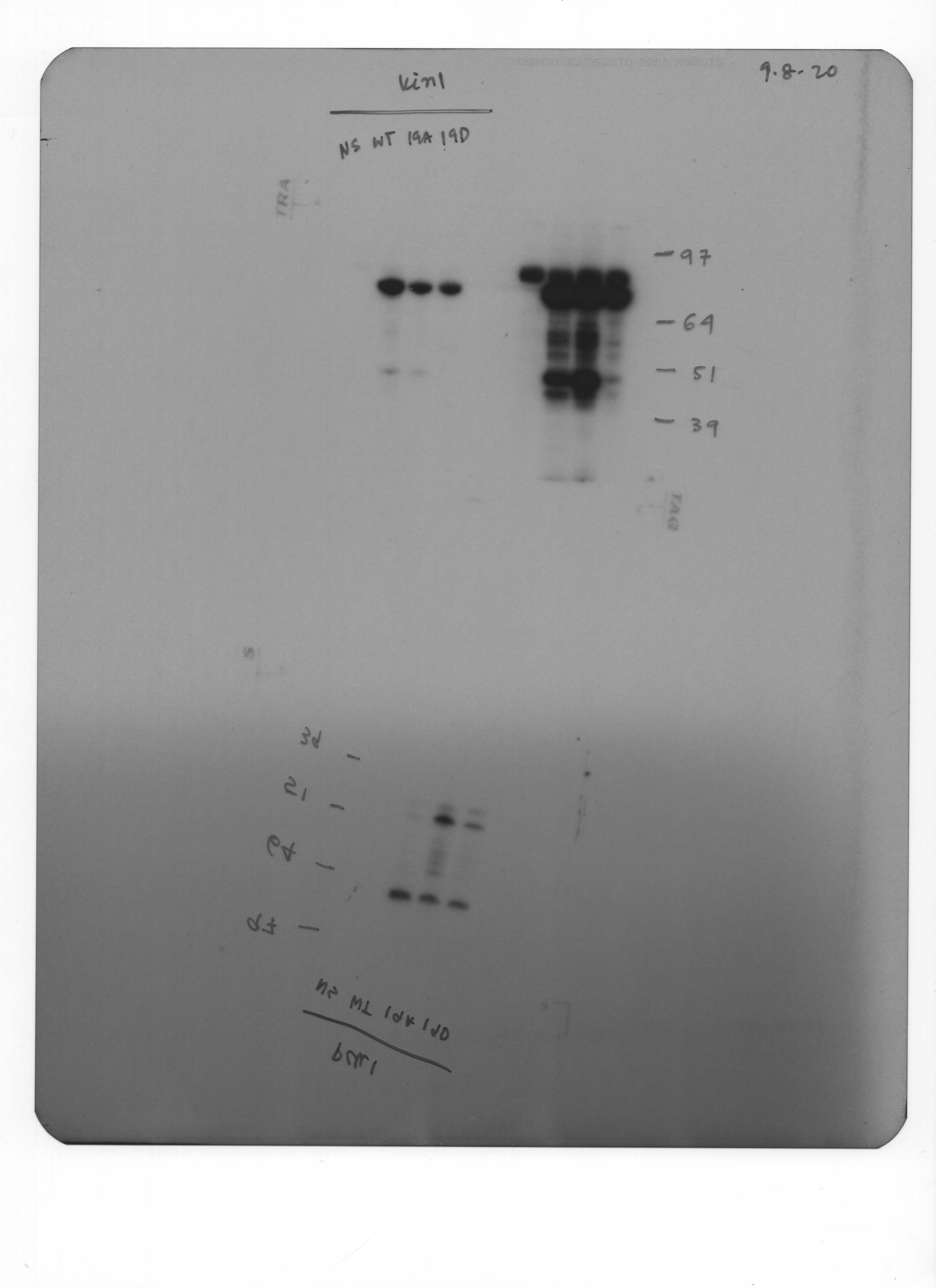

Supplement: Figure 2—figure supplement 1—source data 1. [file elife-83062-fig2-figsupp1-data1.zip › Figure 1-figure supplement 1/Figure 1-supplement 1B/p32/kin1(top)-pck1 (bottom)-32p.tif]

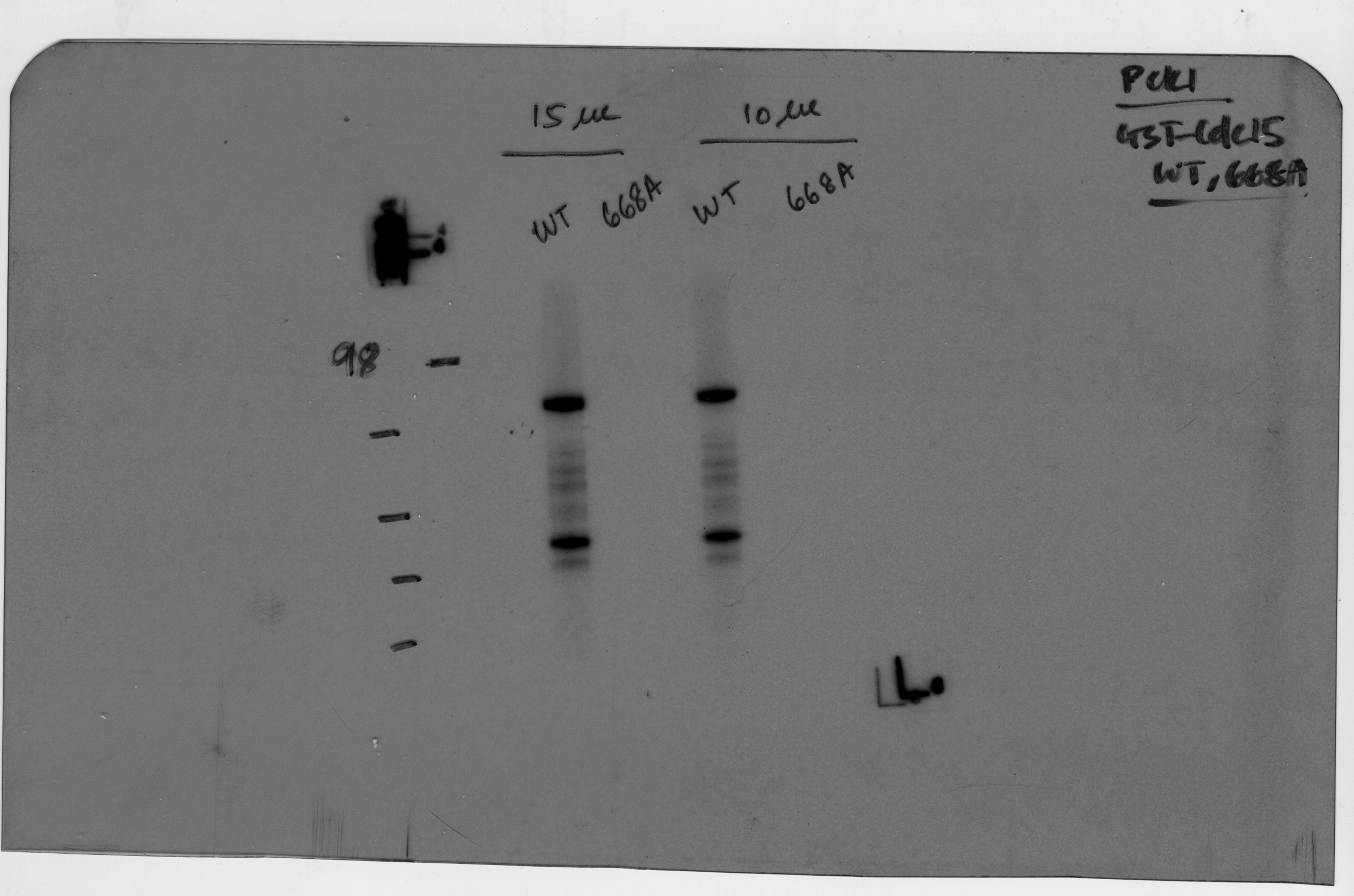

Supplement: Figure 3—source data 1. [file elife-83062-fig3-data1.zip › Figure 3-new source files/Figure 3D-wt and 1A_p32.tif]

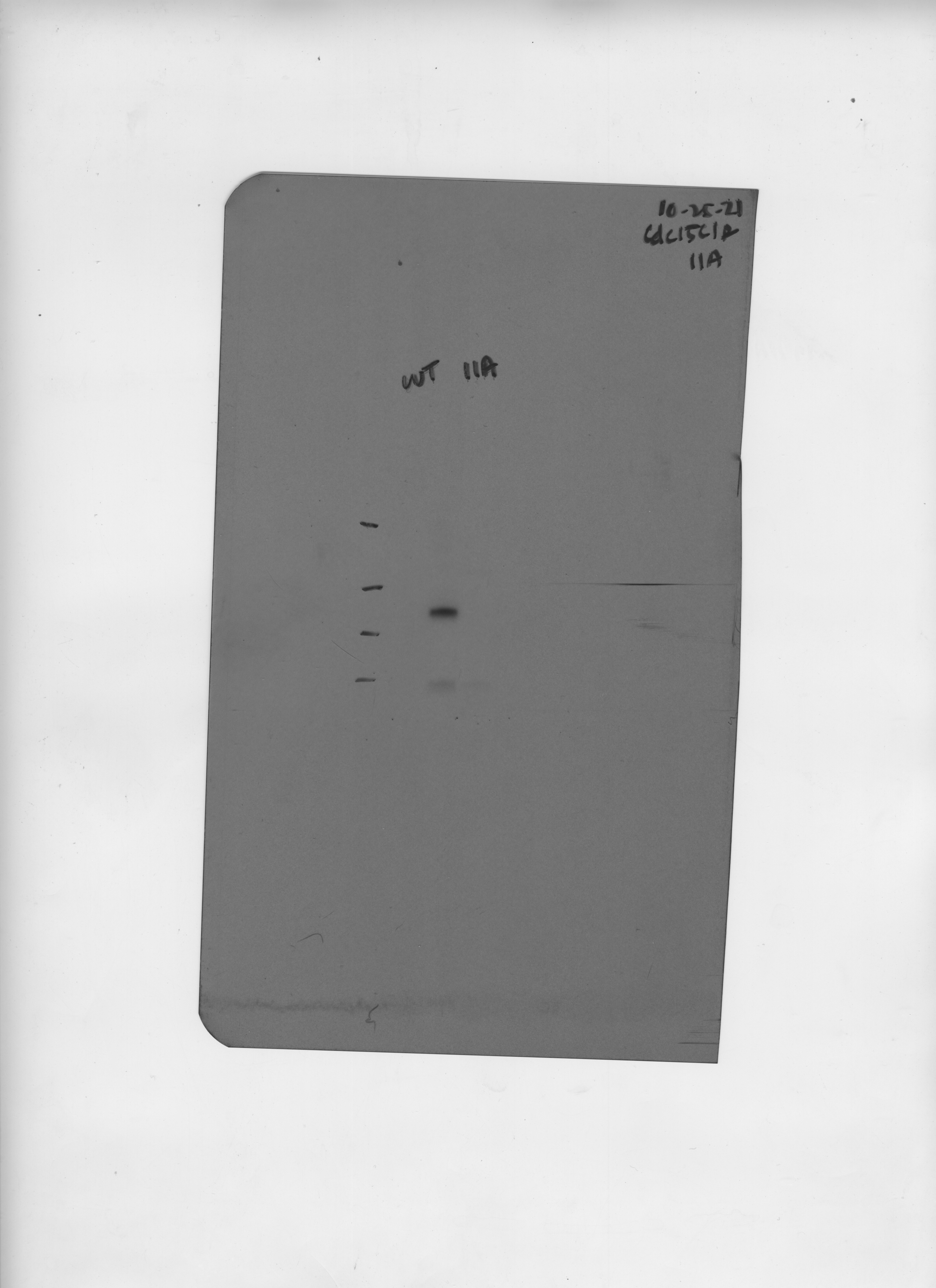

Supplement: Figure 3—source data 1. [file elife-83062-fig3-data1.zip › Figure 3-new source files/Figure 3C-wt and 11A-p32.tif]

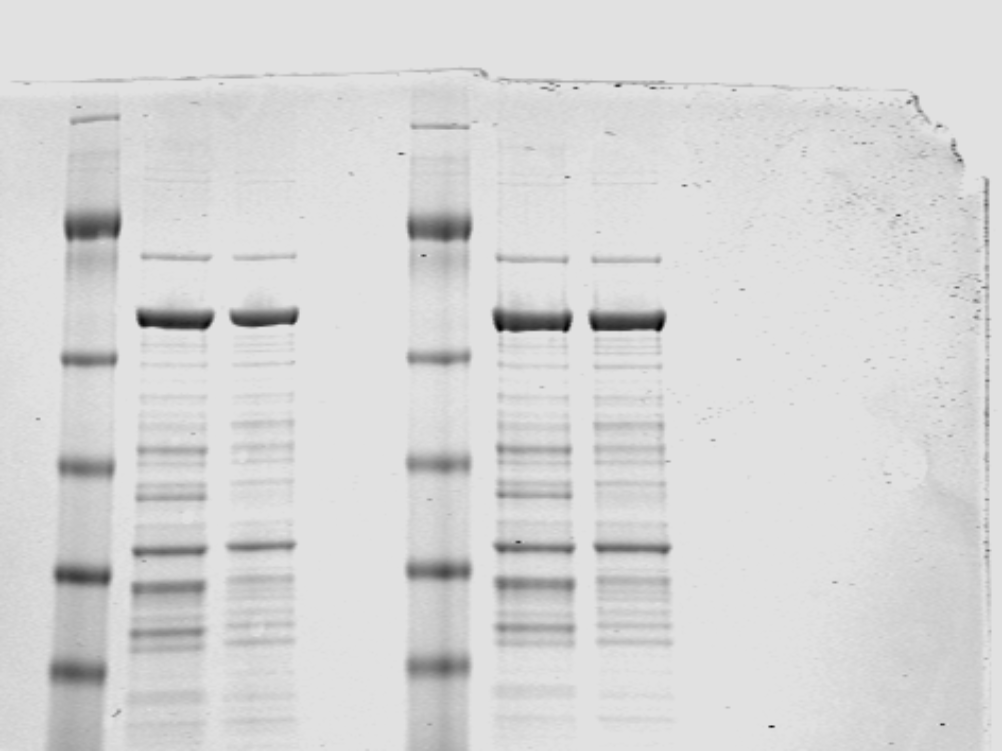

Supplement: Figure 3—source data 1. [file elife-83062-fig3-data1.zip › Figure 3-new source files/Figure 3B-wt and 5A_replicates- Coomassie stain.tif]

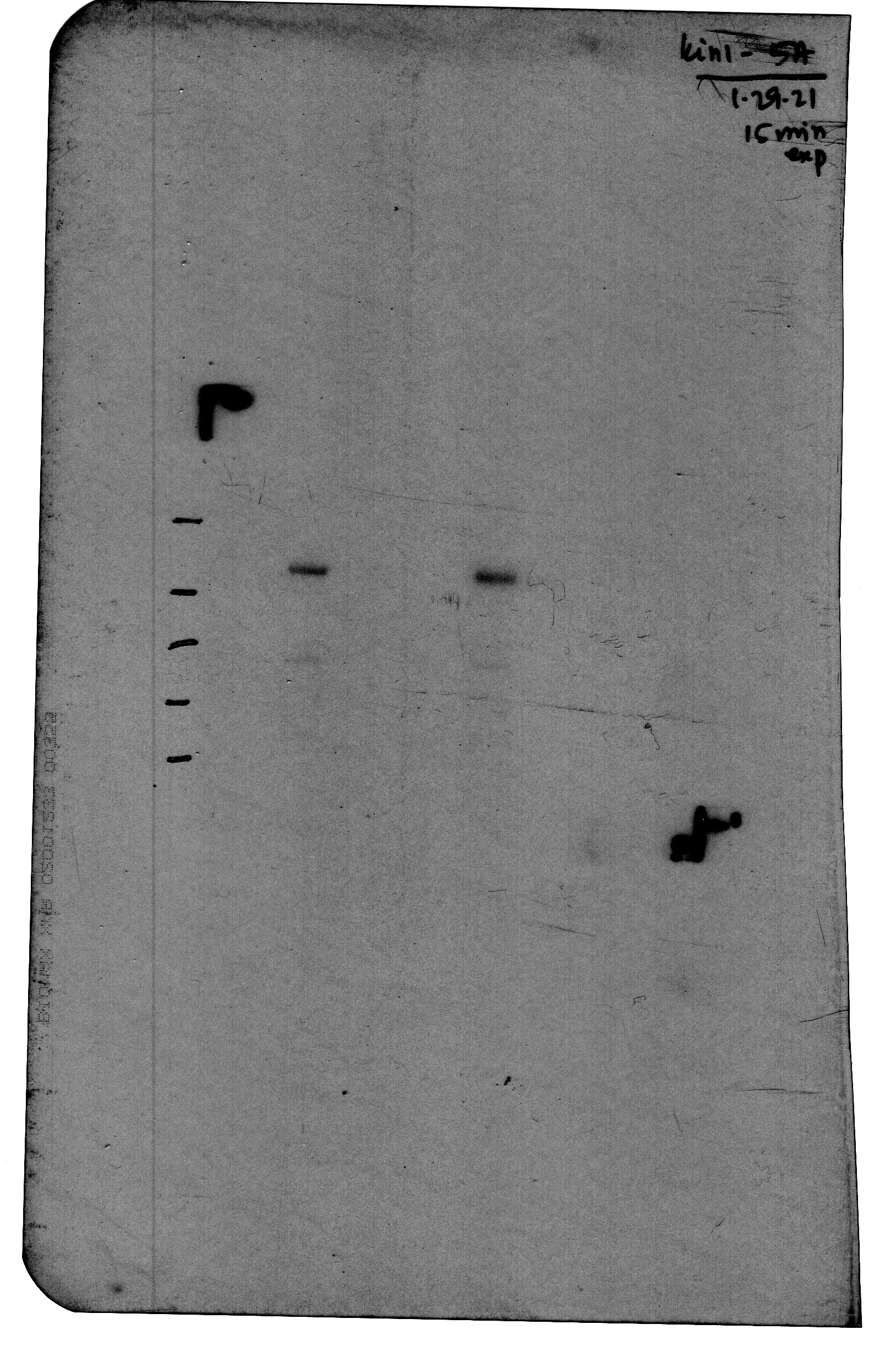

Supplement: Figure 3—source data 1. [file elife-83062-fig3-data1.zip › Figure 3-new source files/Figure 3B-wt and 5A_replicates_p32.tif]

Figure 3B

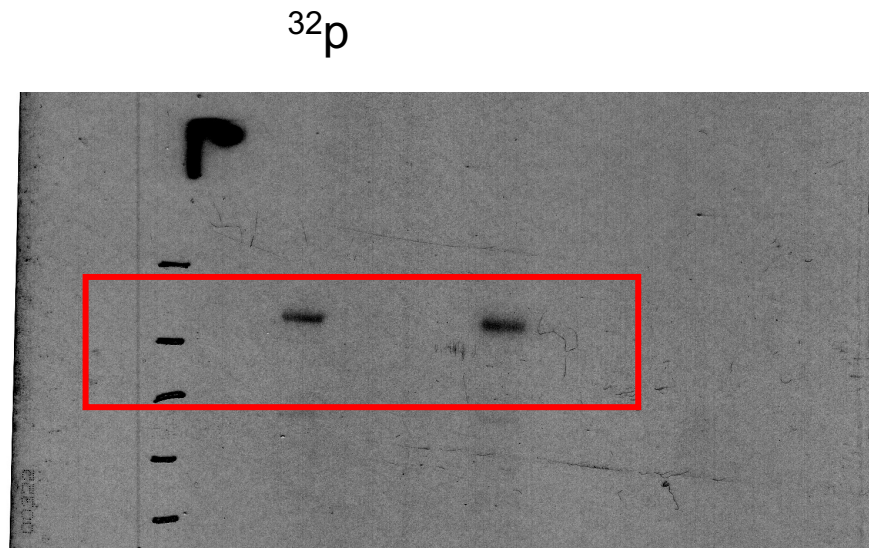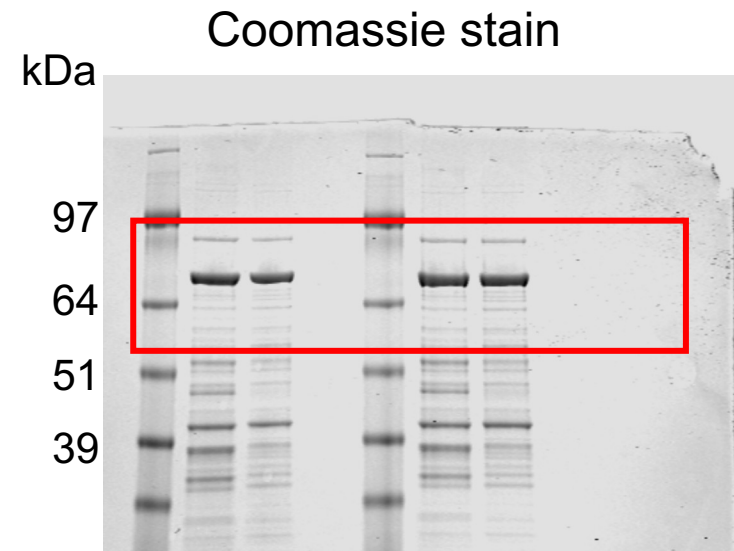

=GST-Cdc15C

Figure 3C

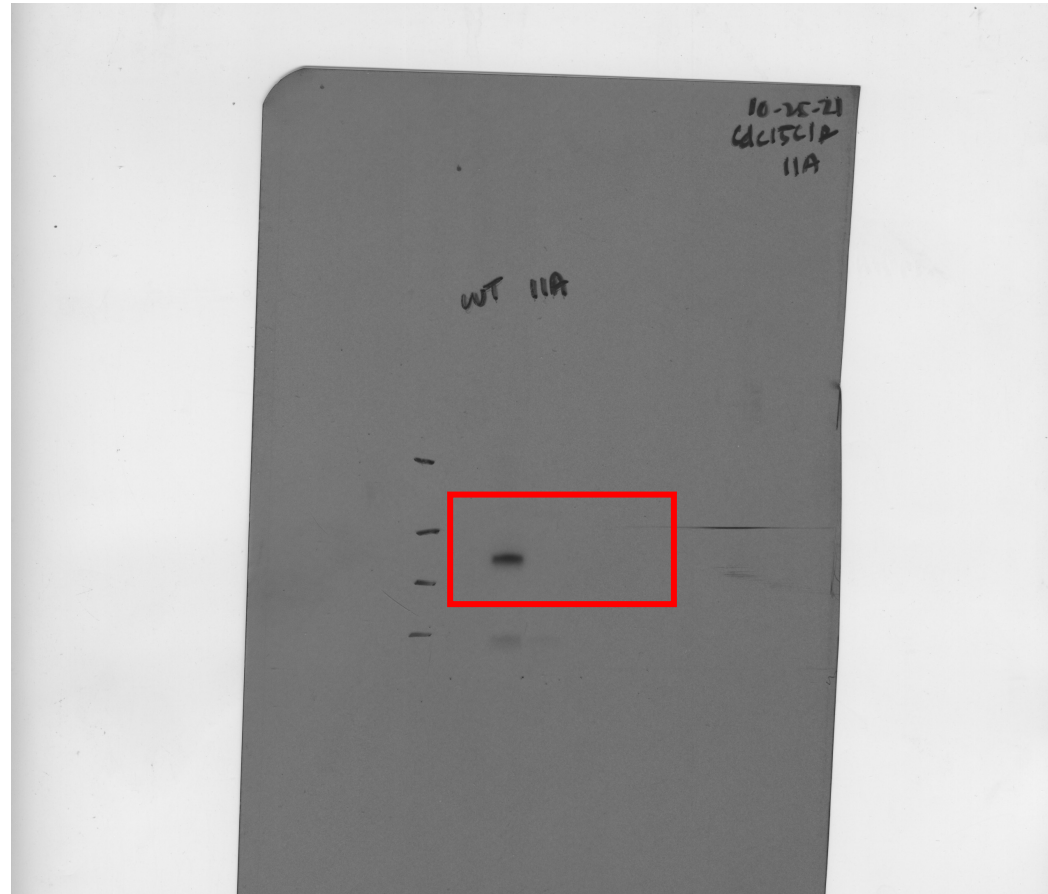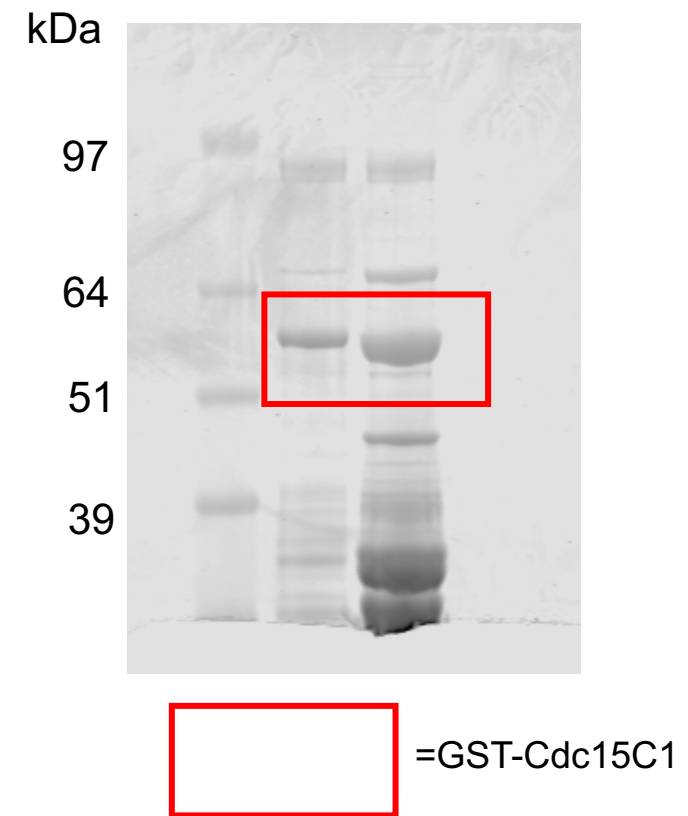

Figure 3D

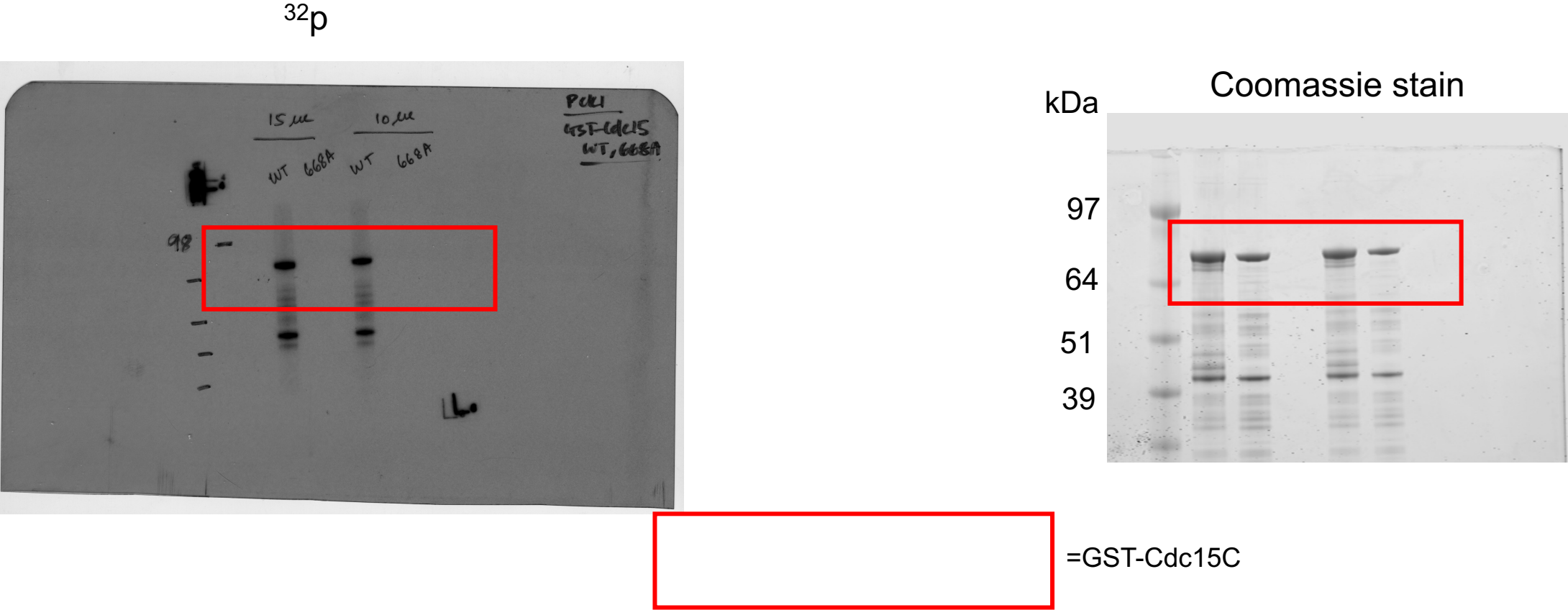

Figure 3E

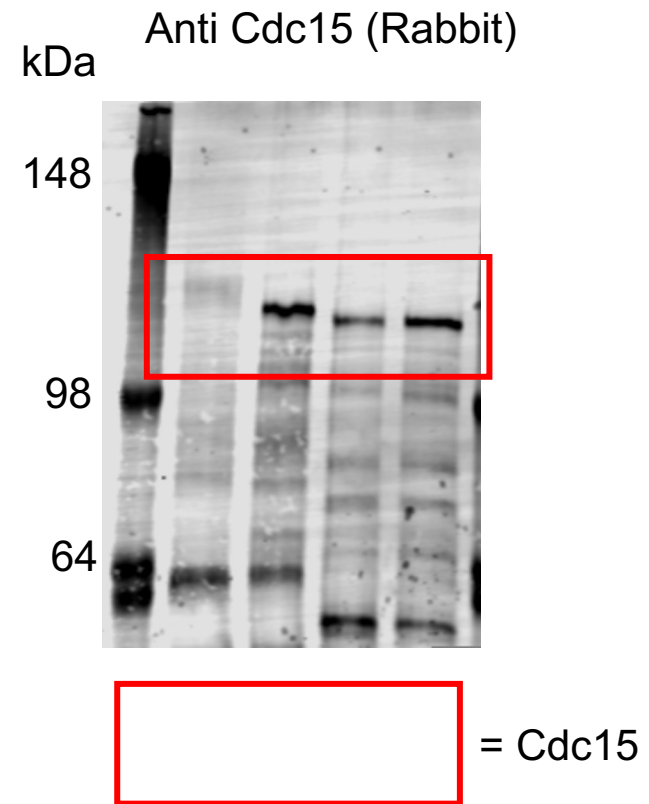

Figure 3F

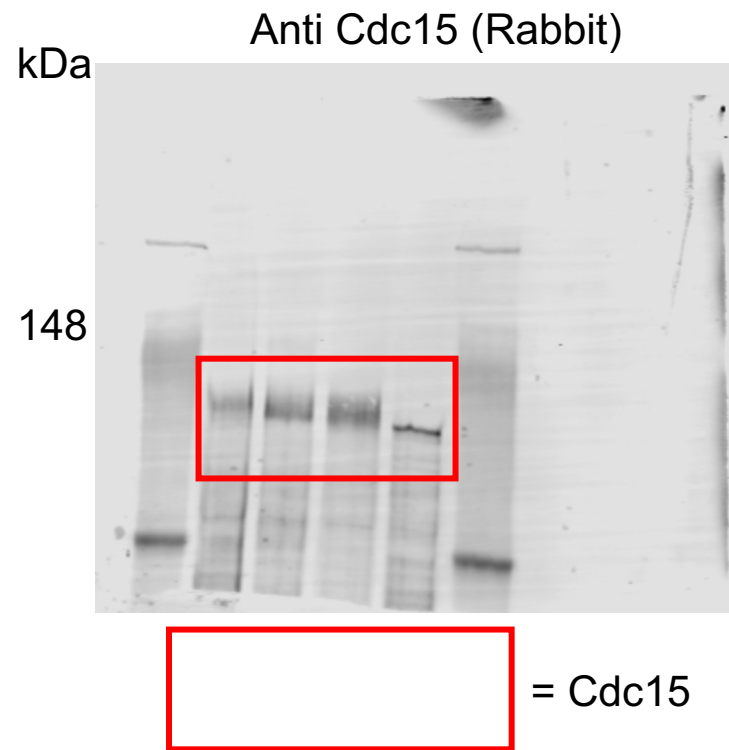

Supplement: Figure 3—source data 1. [file elife-83062-fig3-data1.zip › Figure 3-new source files/Figure 3-Source file-labeled.pdf]

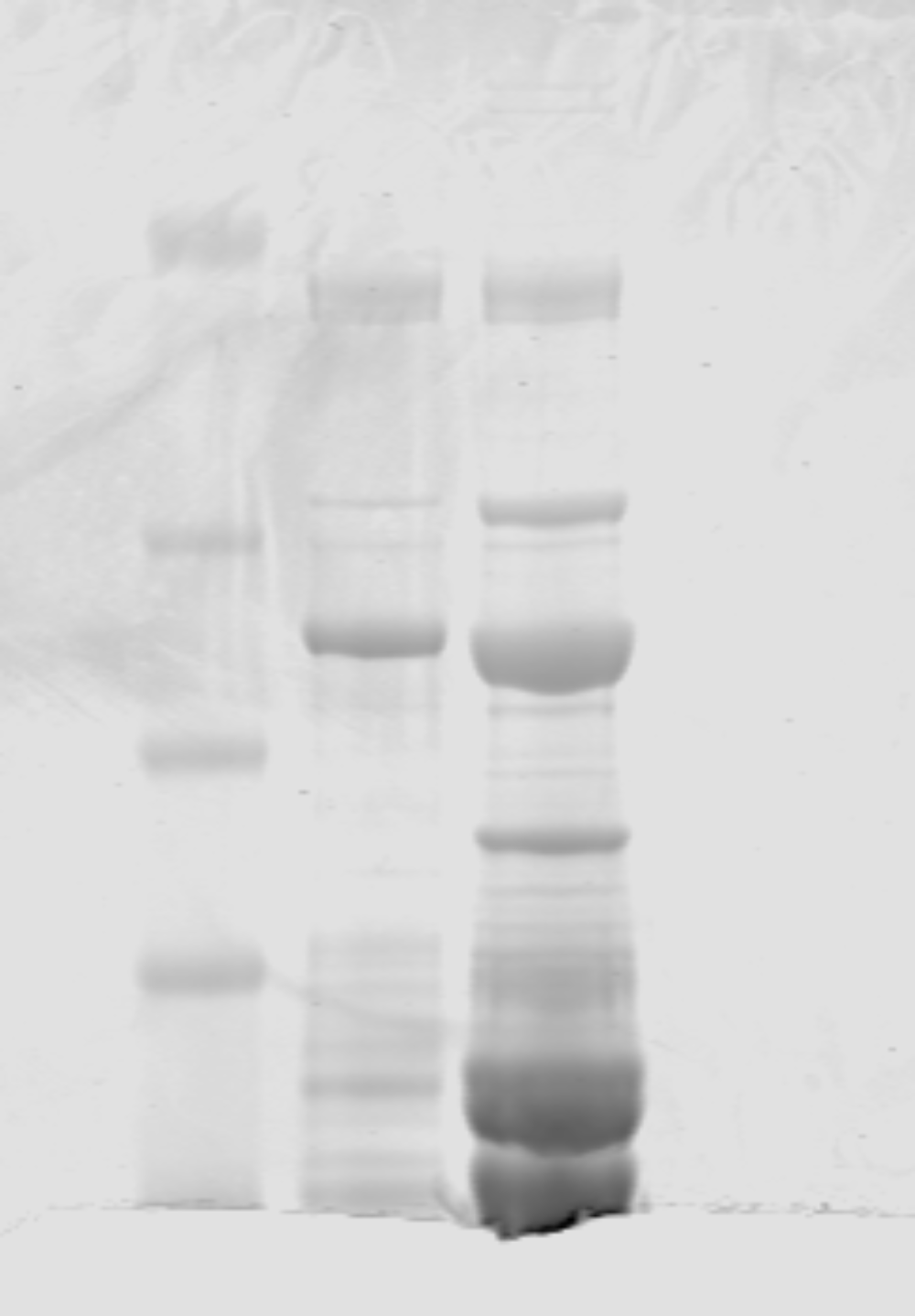

Supplement: Figure 3—source data 1. [file elife-83062-fig3-data1.zip › Figure 3-new source files/Figure 3C-wt and 11A-Coomassie stain.tif]

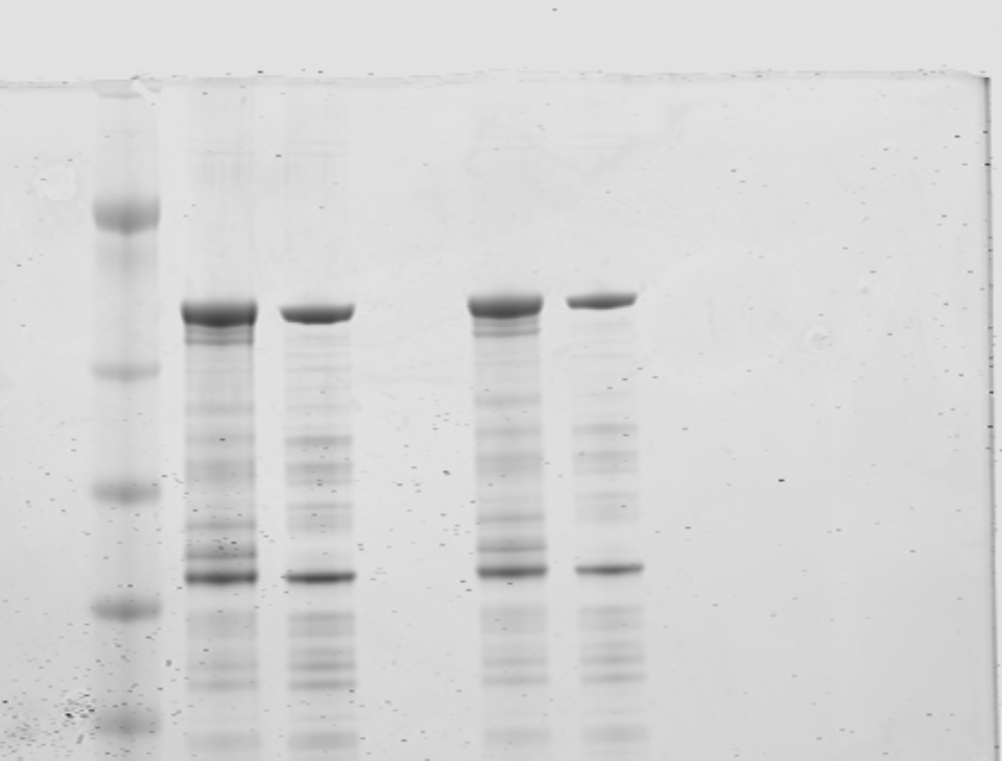

Supplement: Figure 3—source data 1. [file elife-83062-fig3-data1.zip › Figure 3-new source files/Figure 3D-wt and 1A-Coomassie.tif]

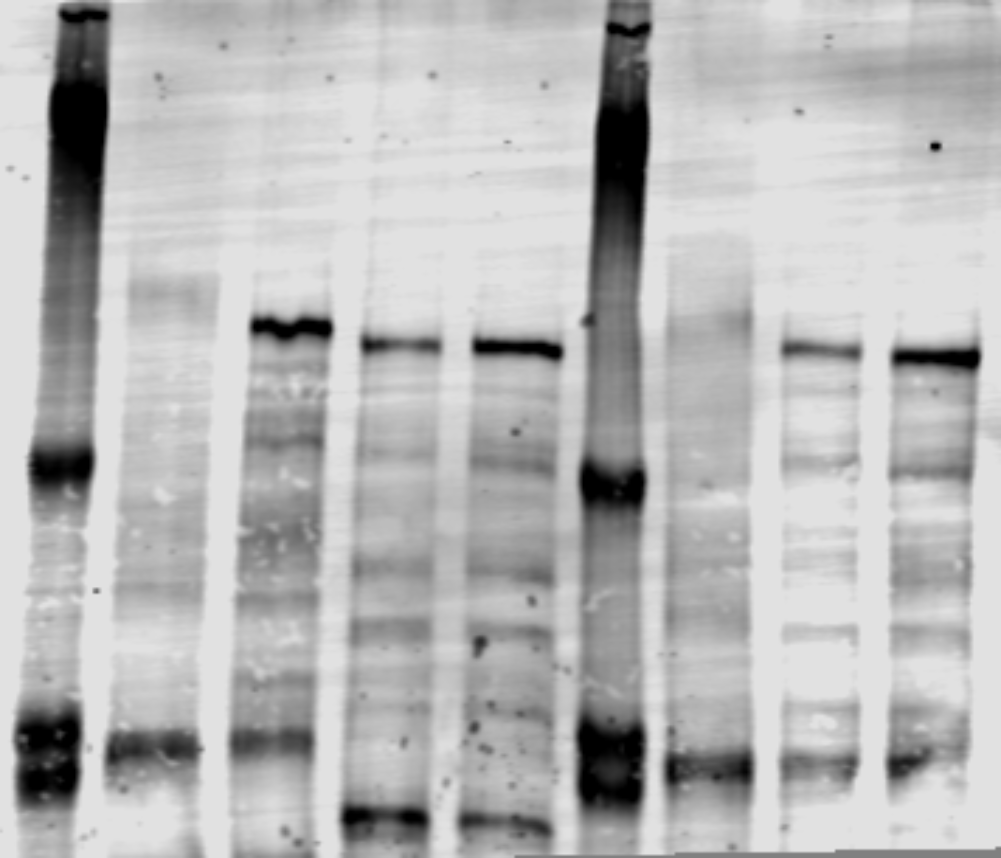

Supplement: Figure 3—source data 1. [file elife-83062-fig3-data1.zip › Figure 3-new source files/Figure 3E-Anti Cdc15-wt and 31A.tif]

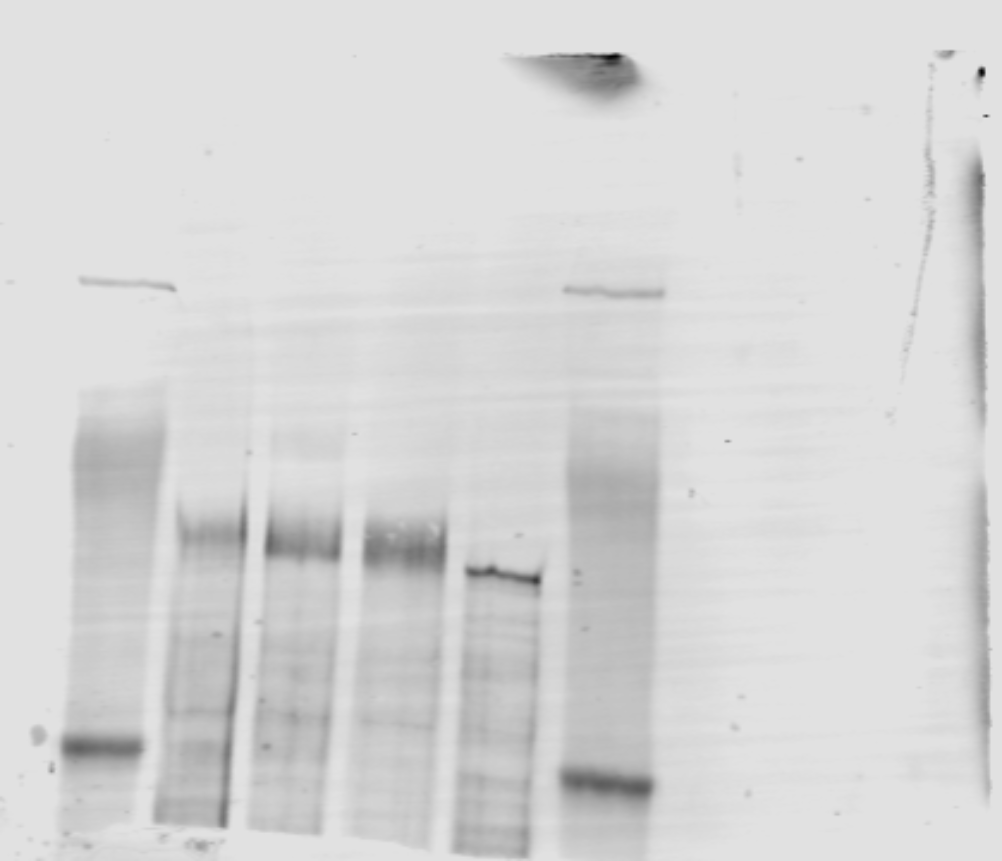

Supplement: Figure 3—source data 1. [file elife-83062-fig3-data1.zip › Figure 3-new source files/Figure 3F-Anti Cdc15_nda3_cps1_31A.tif]

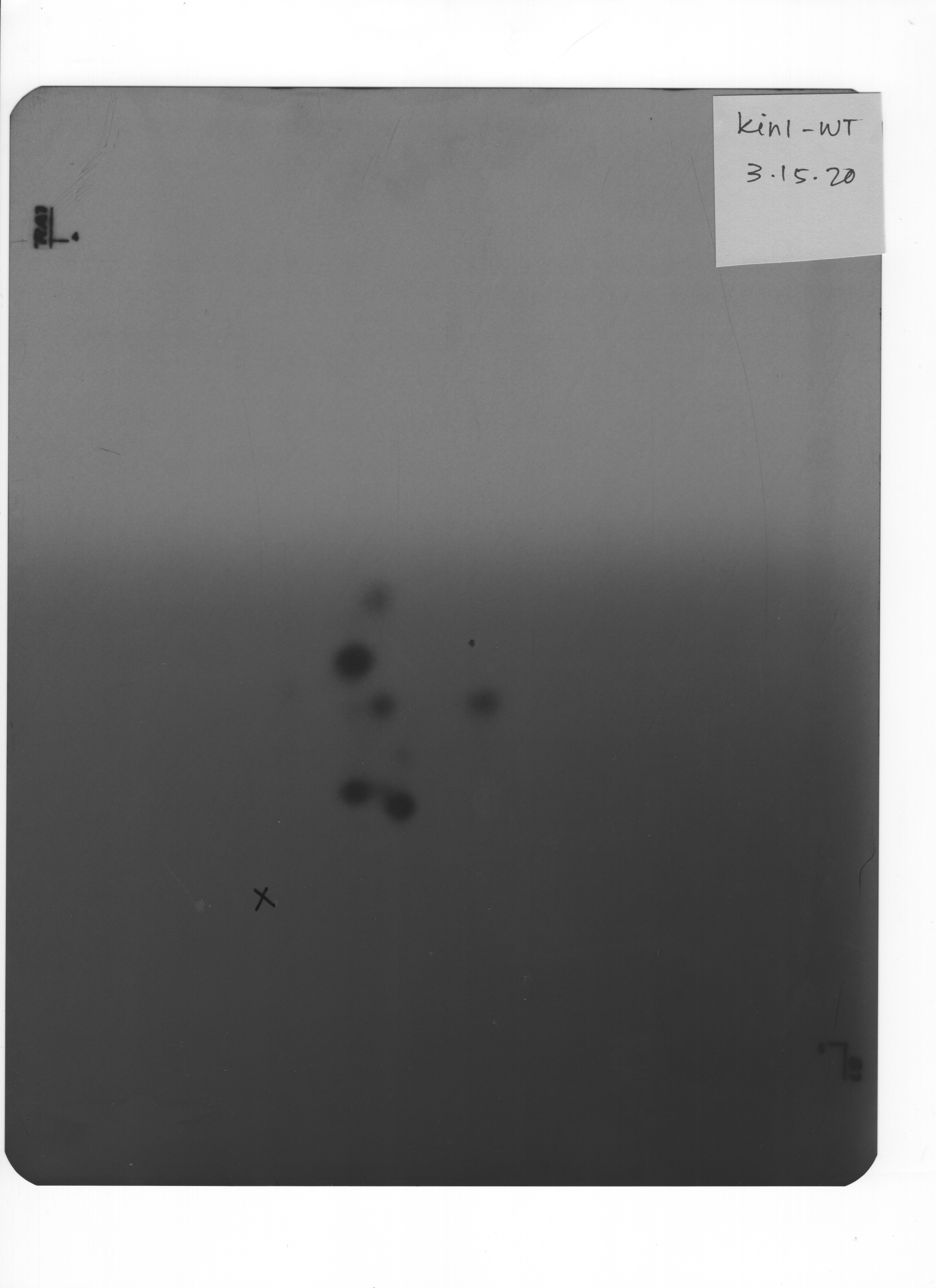

Supplement: Figure 3—figure supplement 1—source data 1. [file elife-83062-fig3-figsupp1-data1.zip › Figure 3-figure supplement 1/kin1-wt.tif]

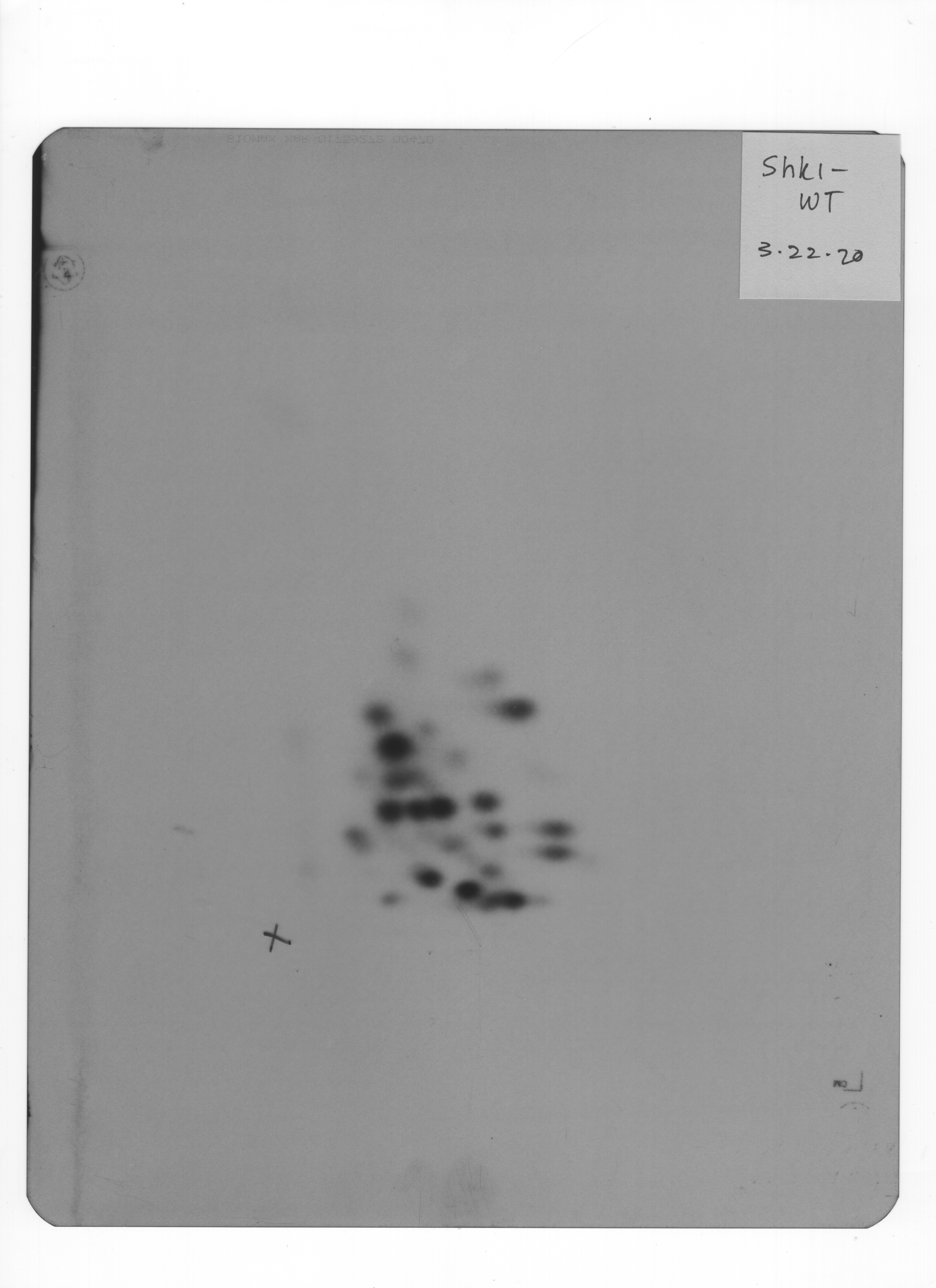

Supplement: Figure 3—figure supplement 2—source data 1. [file elife-83062-fig3-figsupp2-data1.zip › Figure 3-figure supplement 2/shk1-wt.tif]

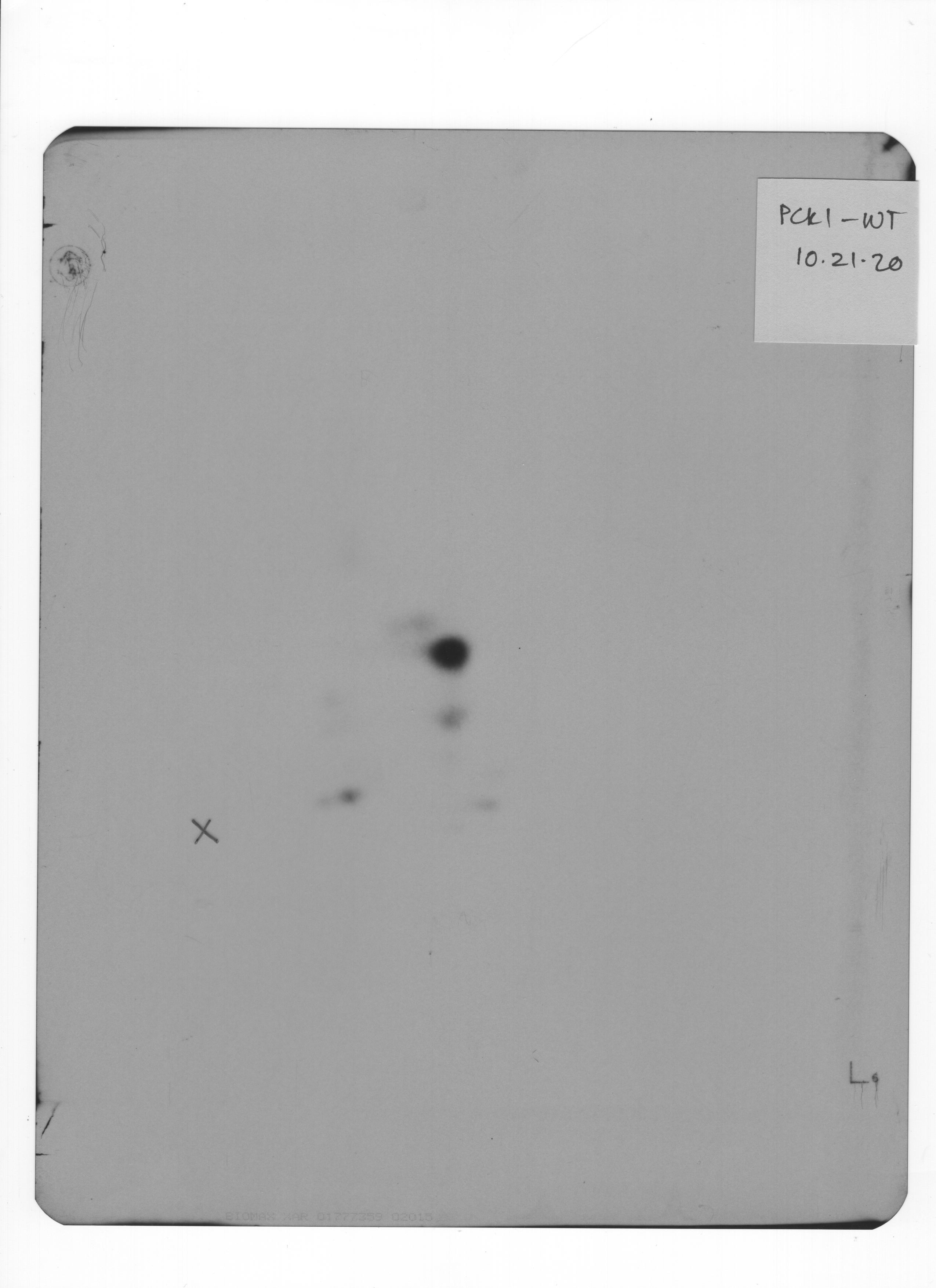

Supplement: Figure 3—figure supplement 3—source data 1. [file elife-83062-fig3-figsupp3-data1.zip › Figure 3-figure supplement 3/pck1-wt.tif]

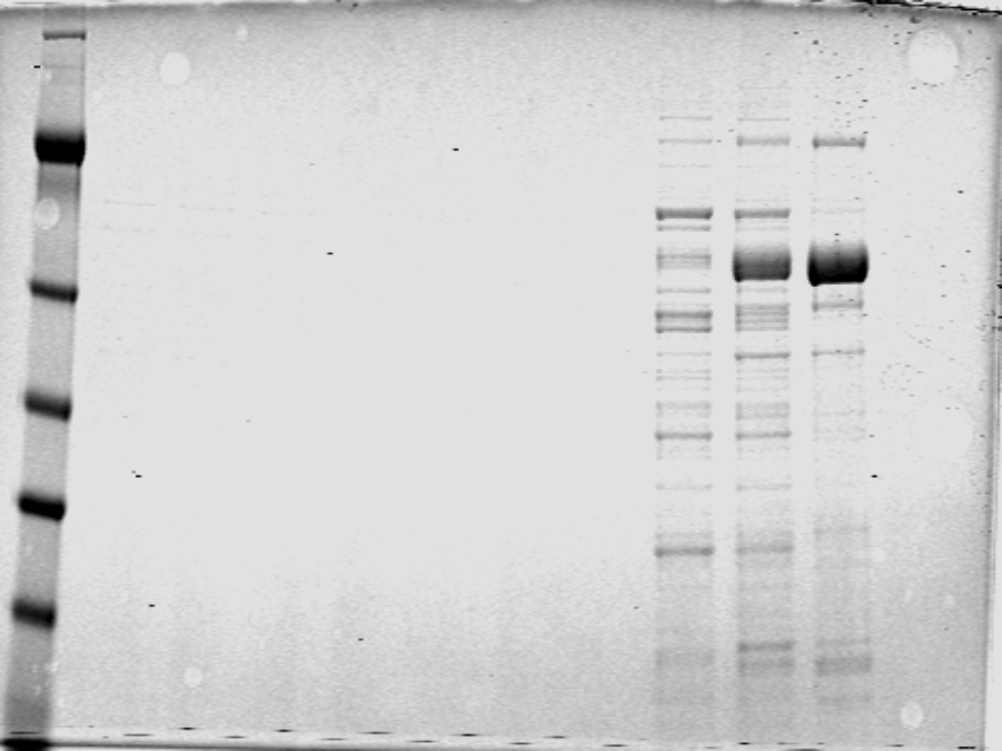

Supplement: Figure 7—figure supplement 1—source data 1. [file elife-83062-fig7-figsupp1-data1.zip › Figure 7-figure supplement 1/Figure 7-figure supplement 1A- Coomassie stain.tif]

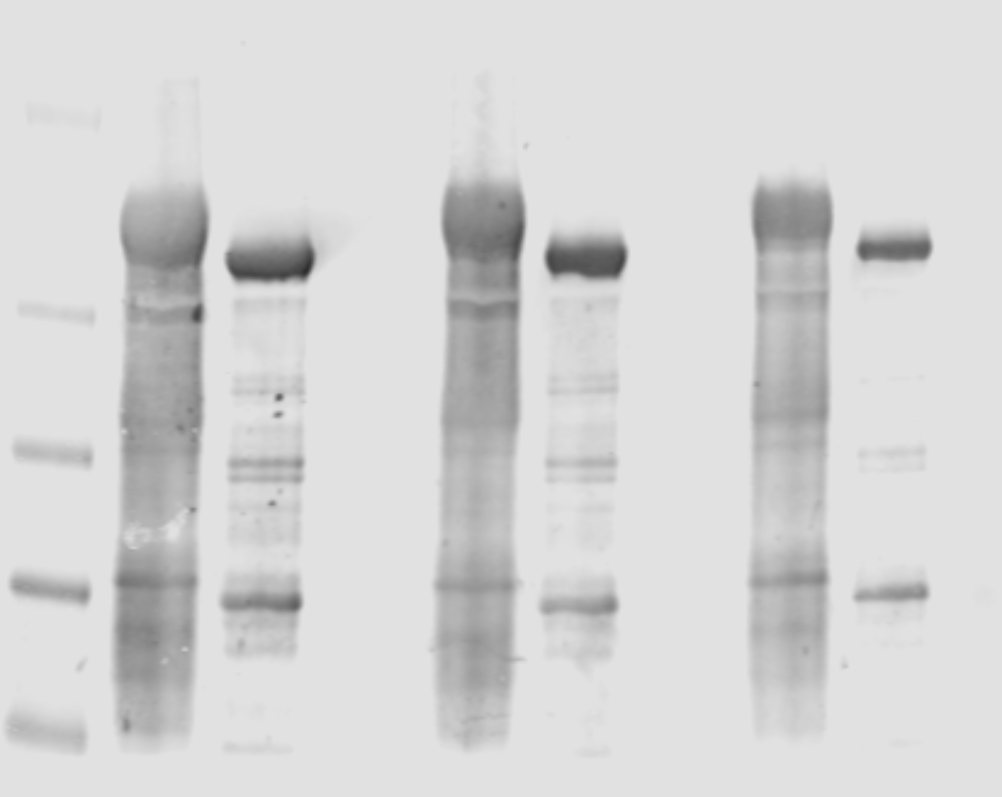

Supplement: Figure 7—figure supplement 1—source data 1. [file elife-83062-fig7-figsupp1-data1.zip › Figure 7-figure supplement 1/Figure 7-figure supplement 1B-Anti Cdc15.tif]

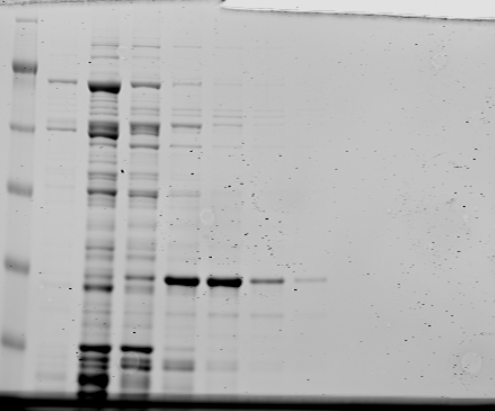

Supplement: Figure 8—figure supplement 1—source data 1. [file elife-83062-fig8-figsupp1-data1.zip › Figure 8-figure supplement 1/Figure 8-figure supplement 1A-Coomassie.tif]

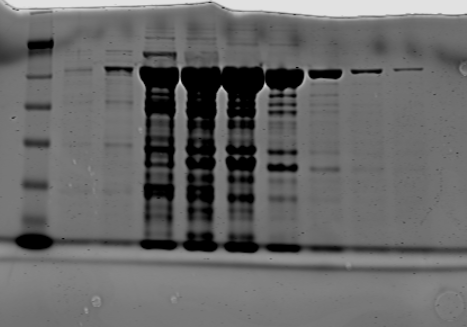

Supplement: Figure 8—figure supplement 1—source data 1. [file elife-83062-fig8-figsupp1-data1.zip › Figure 8-figure supplement 1/Figure 8-figure supplement 1C-Coomassie.tif]

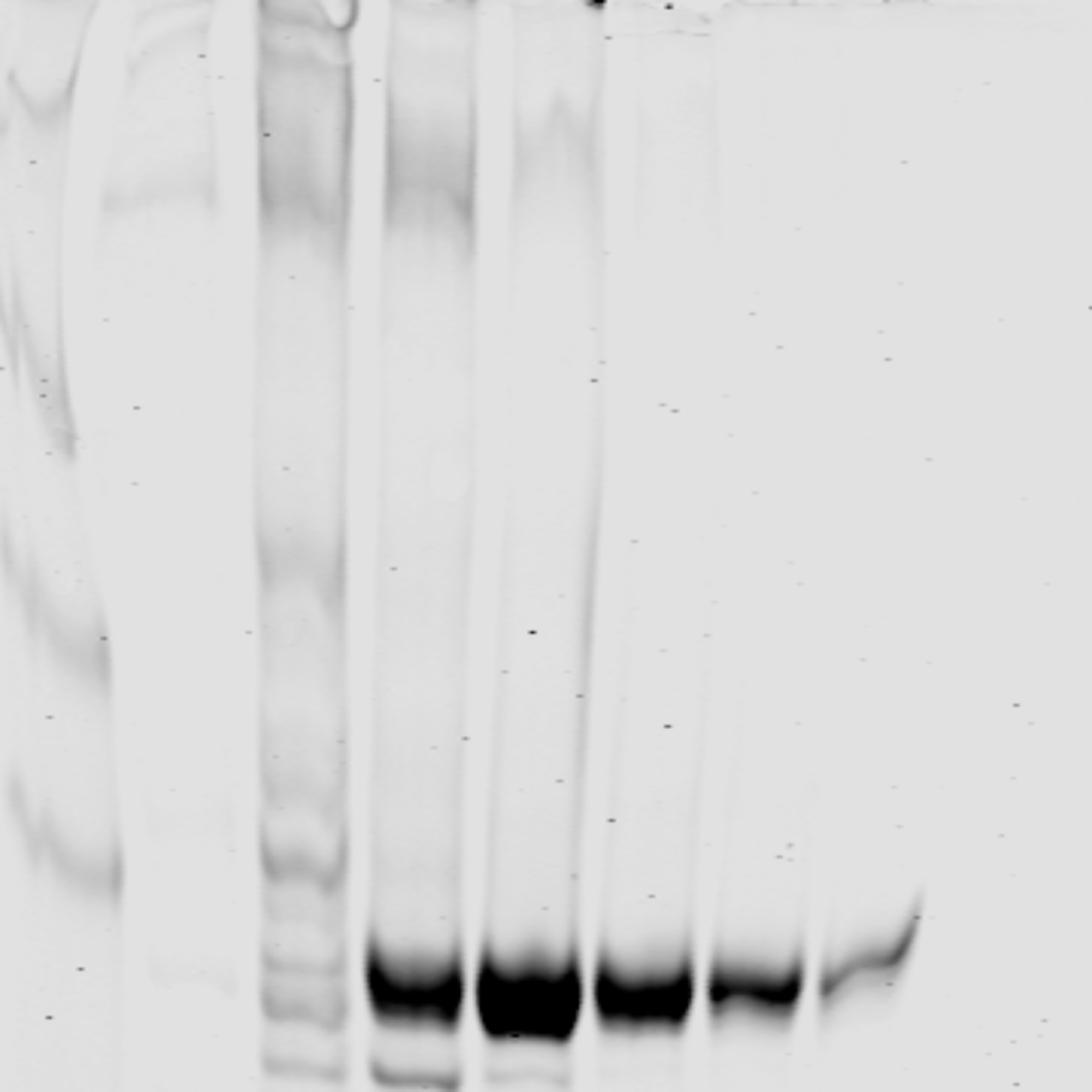

Supplement: Figure 8—figure supplement 1—source data 1. [file elife-83062-fig8-figsupp1-data1.zip › Figure 8-figure supplement 1/Figure 8-figure supplement 1B-Comassie.tif]

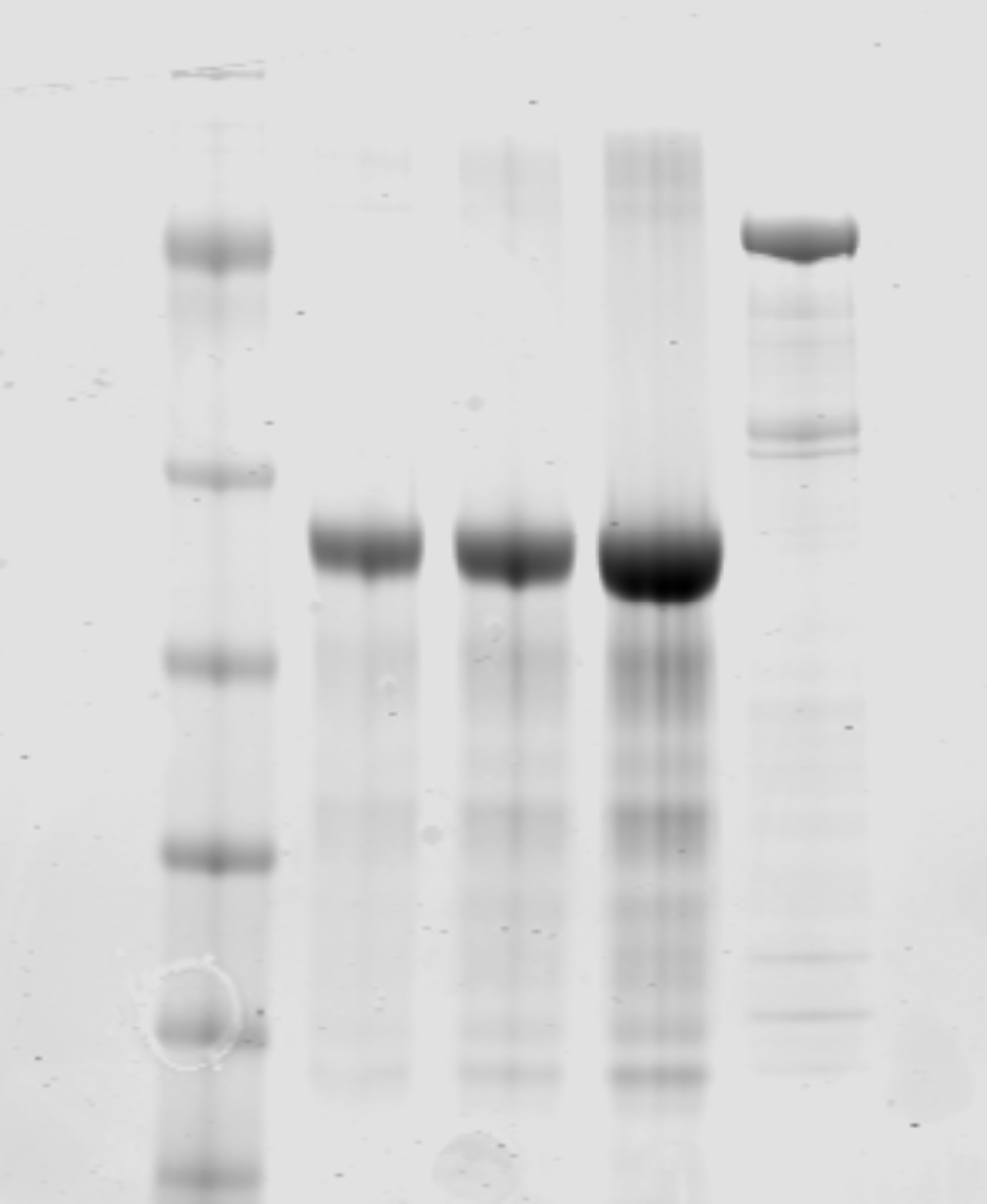

Supplement: Figure 9—figure supplement 1—source data 1. [file elife-83062-fig9-figsupp1-data1.zip › Figure 9-figure supplement 1/Figure 9-figure supplement 1A-Coomassie.tif]

# Figure 9-figure supplement 1A

## Coomassie stain

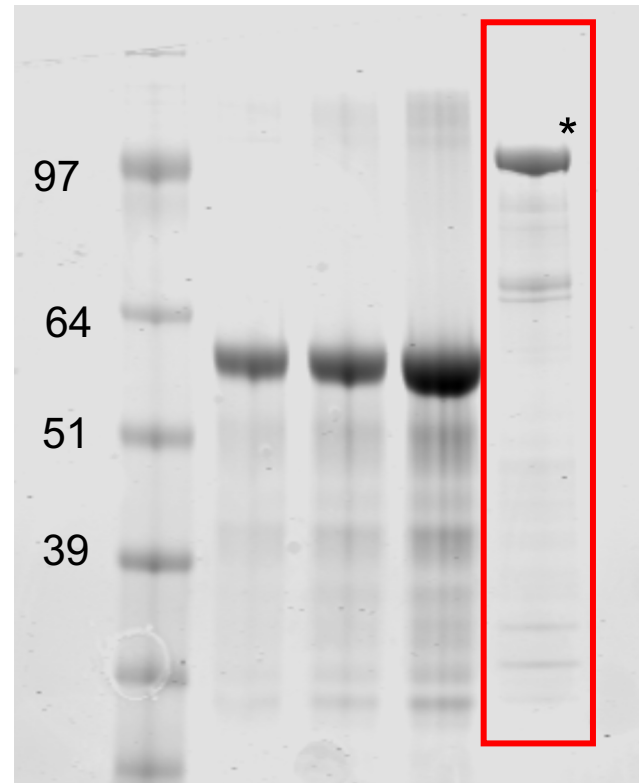

=Cdc15 (E30K, E152K)

Supplement: Figure 9—figure supplement 1—source data 1. [file elife-83062-fig9-figsupp1-data1.zip › Figure 9-figure supplement 1/Figure 9-figure supplement 1.pdf]

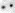

Supplement: Figure 10—source data 1. [file elife-83062-fig10-data1.zip › Figure 10/9C/Fusion-raw-2.tif]

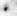

Supplement: Figure 10—source data 1. [file elife-83062-fig10-data1.zip › Figure 10/9C/Fission_raw.tif]

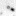

Supplement: Figure 10—source data 1. [file elife-83062-fig10-data1.zip › Figure 10/9C/fusion-raw-1.tif]
