# Supplementary material for: Multiple polarity kinases inhibit phase separation of F-BAR protein Cdc15 and antagonize cytokinetic ring assembly in fission yeast
Source: eLife. 2023 Feb 7;12:e83062. doi: 10.7554/eLife.83062 (PMC9904764; doi:10.7554/eLife.83062)
Supplement: Figure 2—source data 1. [file elife-83062-fig2-data1.zip › Figure 2-new source files/Figure 2-source files labeled.pdf]

Figure-2A

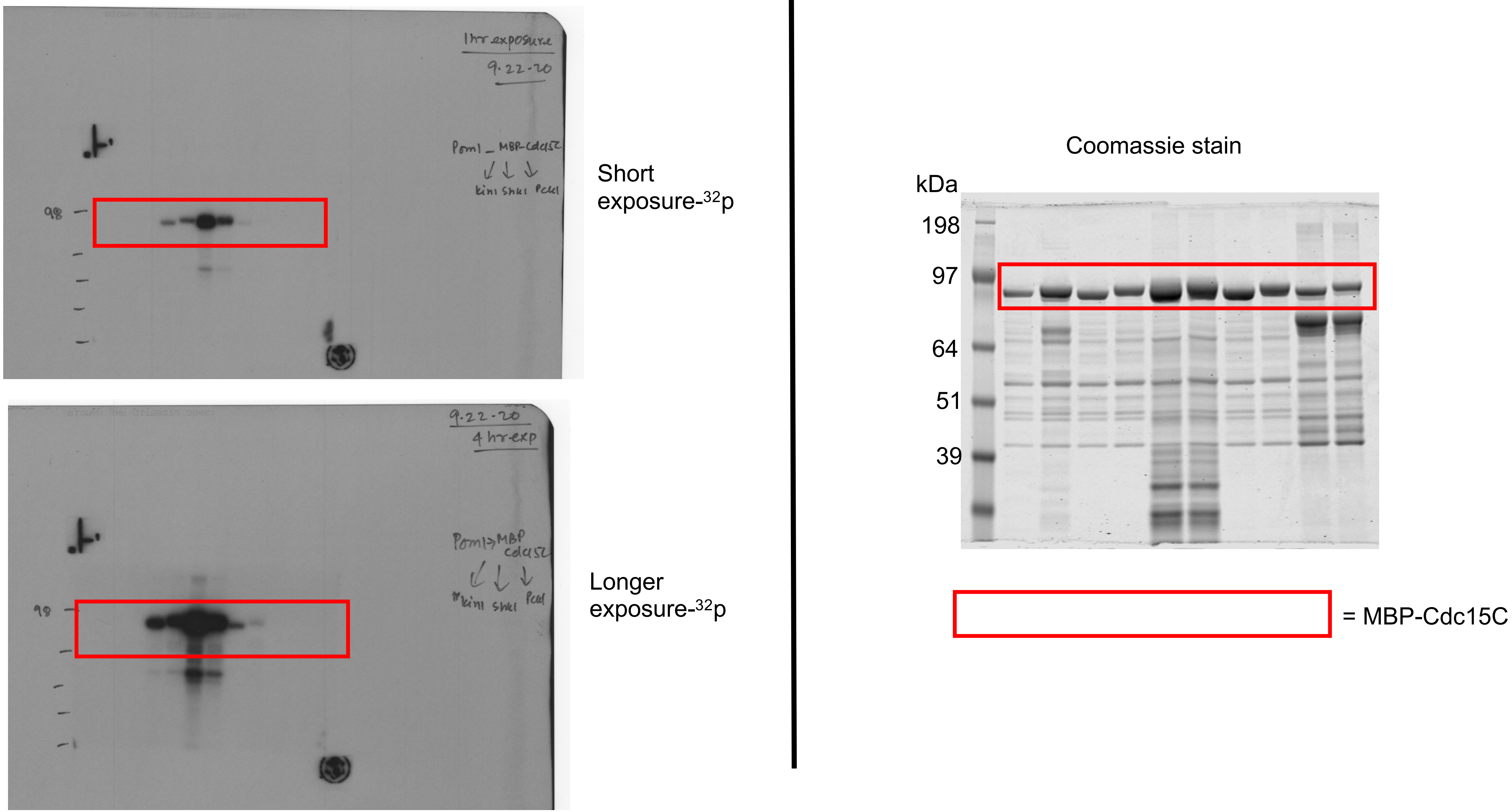
1hr exposure
9.22.20
98
Short exposure-^{32}p
9.22.20
4 hr exp
98
Longer exposure-^{32}p
Coomassie stain
kDa
198
97
64
51
39
= MBP-Cdc15C

Figure-2B

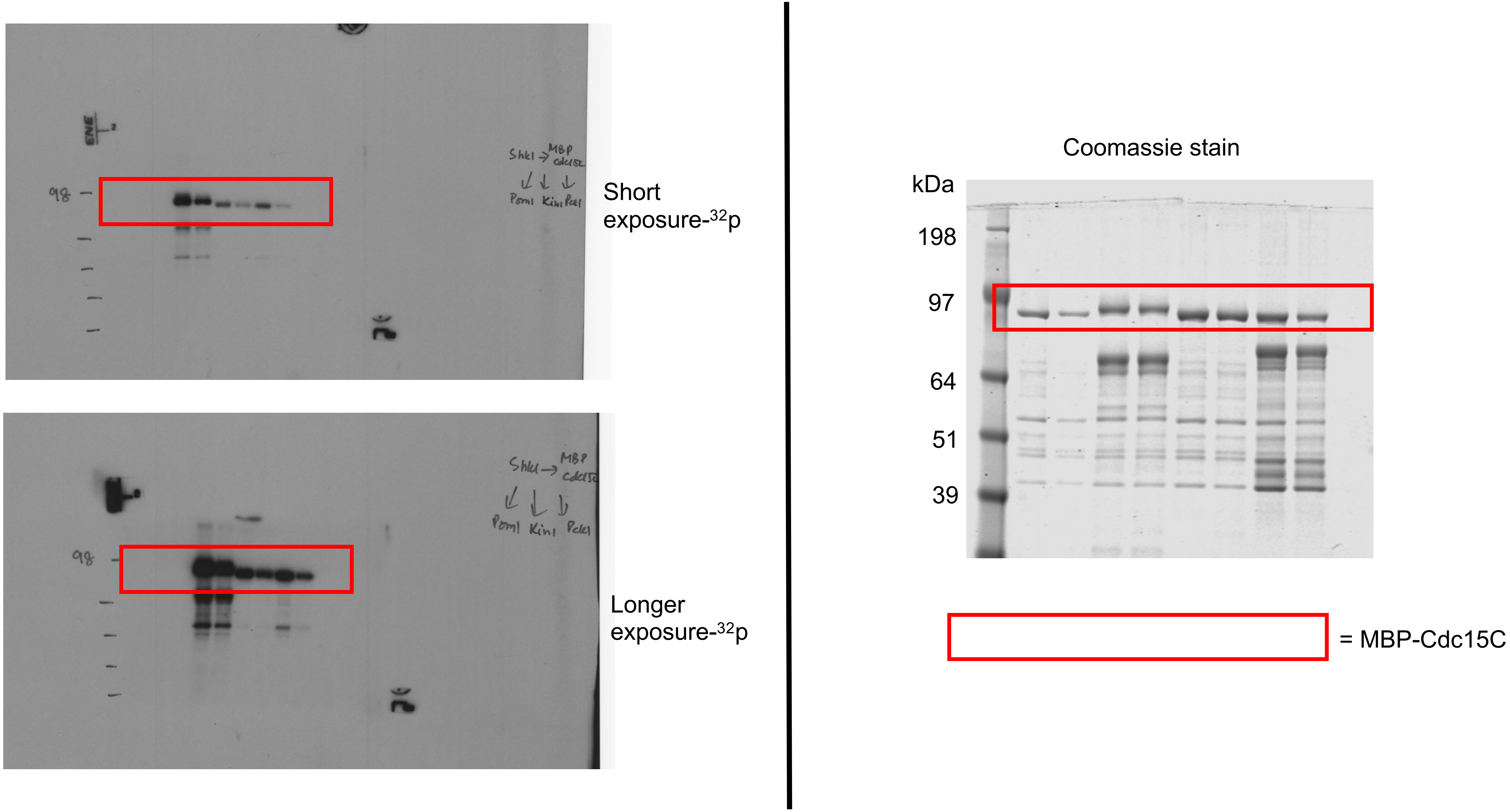
98
Short exposure-^{32}p
98
Longer exposure-^{32}p
Coomassie stain
kDa
198
97
64
51
39
= MBP-Cdc15C

Figure-2C

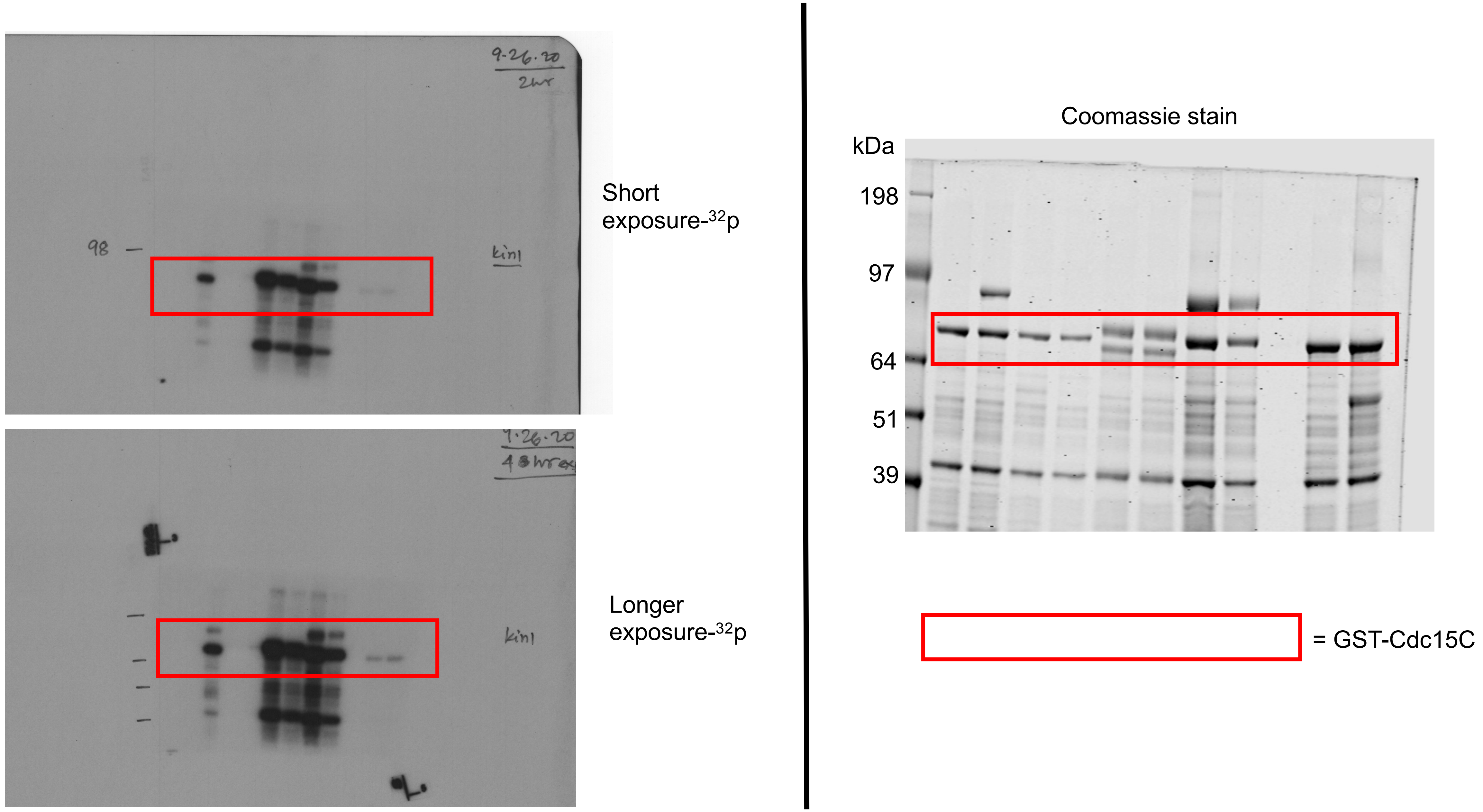
9-26-20
2hr
98
kin1
Short exposure-^{32}p
kin1
Longer exposure-^{32}p
Coomassie stain
kDa
198
97
64
51
39
= GST-Cdc15C

Figure-2D

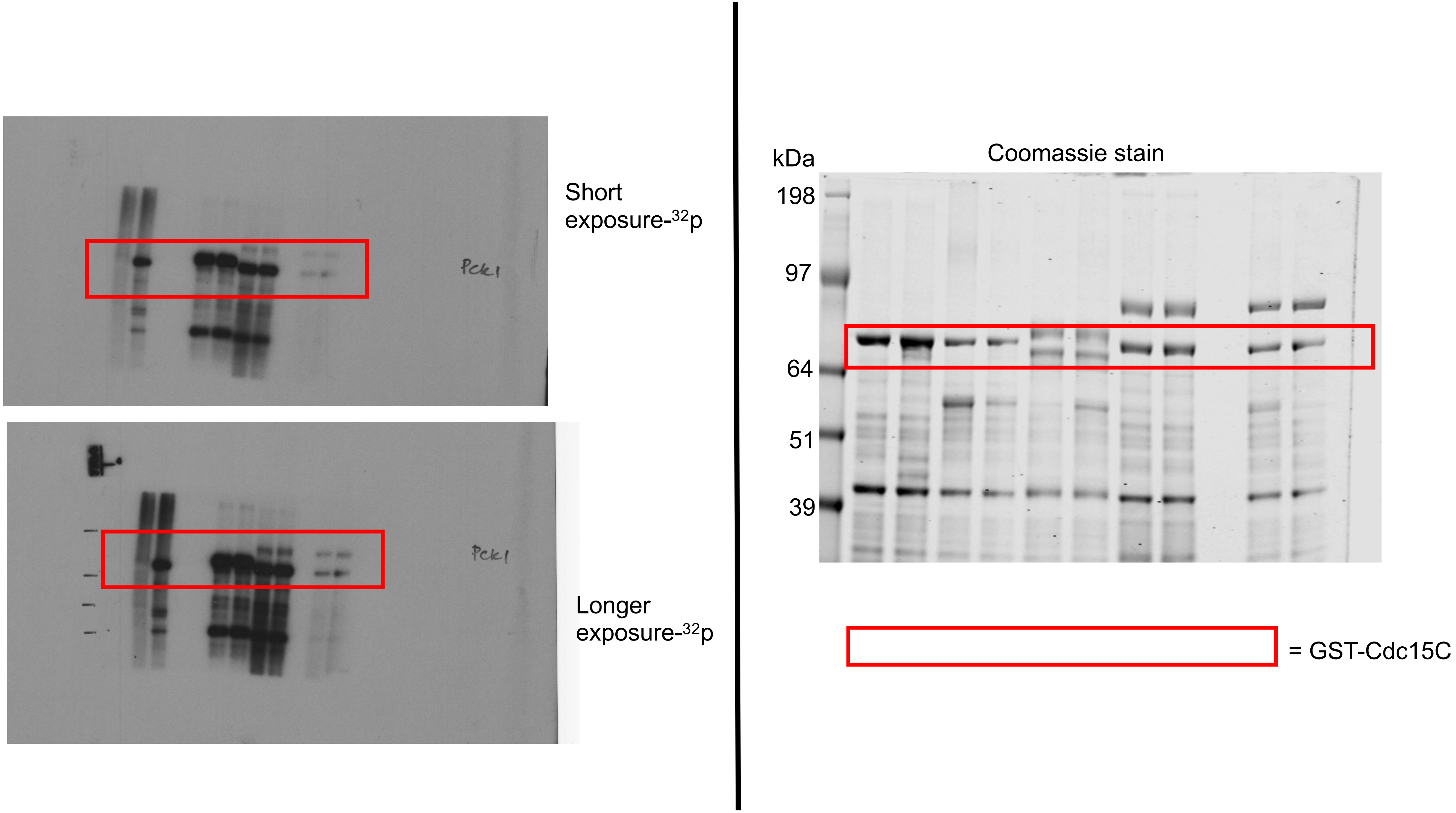
Short
exposure-32p
Pck1
Longer
exposure-32p
Pck1
kDa
Coomassie stain
198
97
64
51
39
= GST-Cdc15C
