# Supplementary material for: Multiple polarity kinases inhibit phase separation of F-BAR protein Cdc15 and antagonize cytokinetic ring assembly in fission yeast
Source: eLife. 2023 Feb 7;12:e83062. doi: 10.7554/eLife.83062 (PMC9904764; doi:10.7554/eLife.83062)
Supplement: Figure 2—figure supplement 1—source data 1. [file elife-83062-fig2-figsupp1-data1.zip › Figure 1-figure supplement 1/Figure 1-figure supplement 1.pdf]

# Figure 1-figure supplement-1A (Kin1)

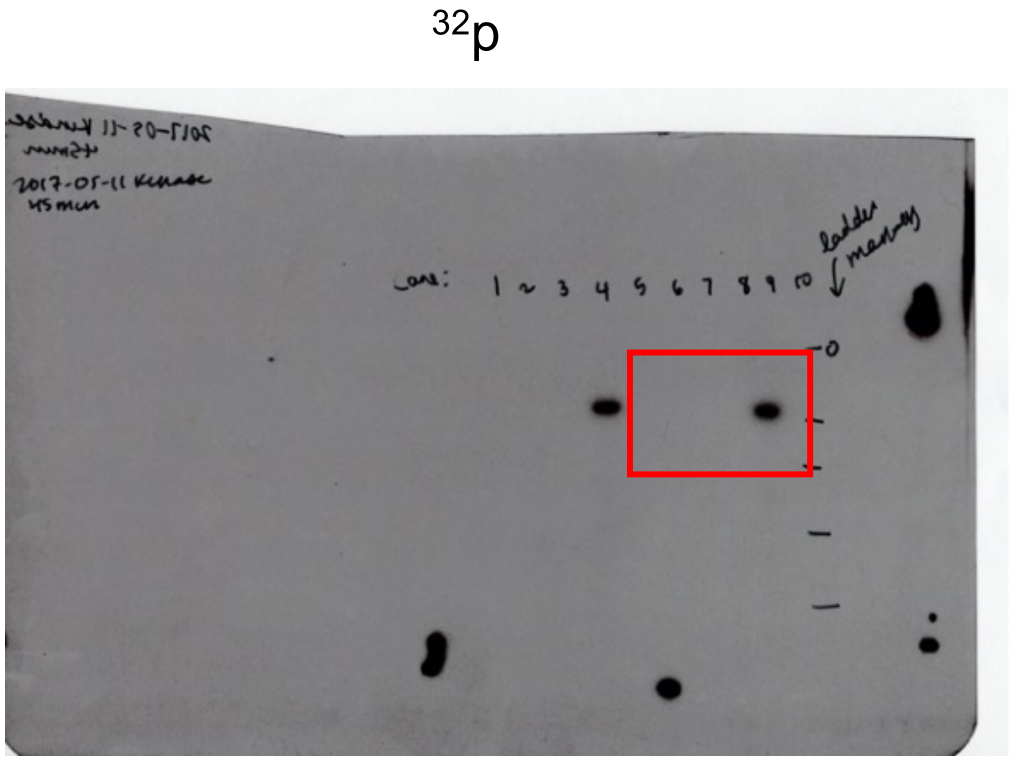


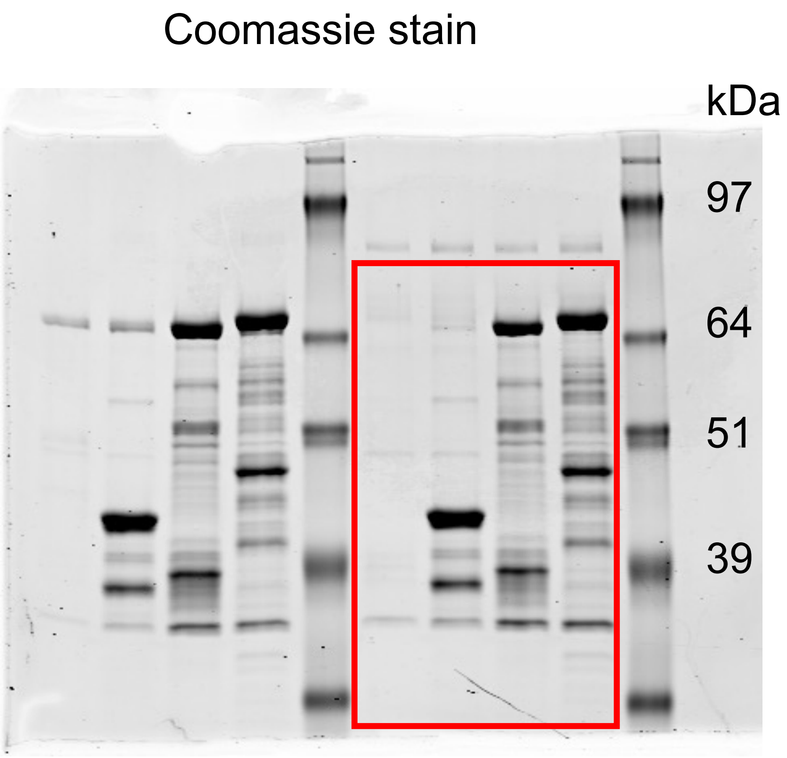


=MBP-Cdc15N, MBP-Cdc15C

=No substrate, MBP, MBP-Cdc15N, MBP-Cdc15C

# Figure 1-figure supplement-1A (Shk1 and Pck1)

$^{32}p$

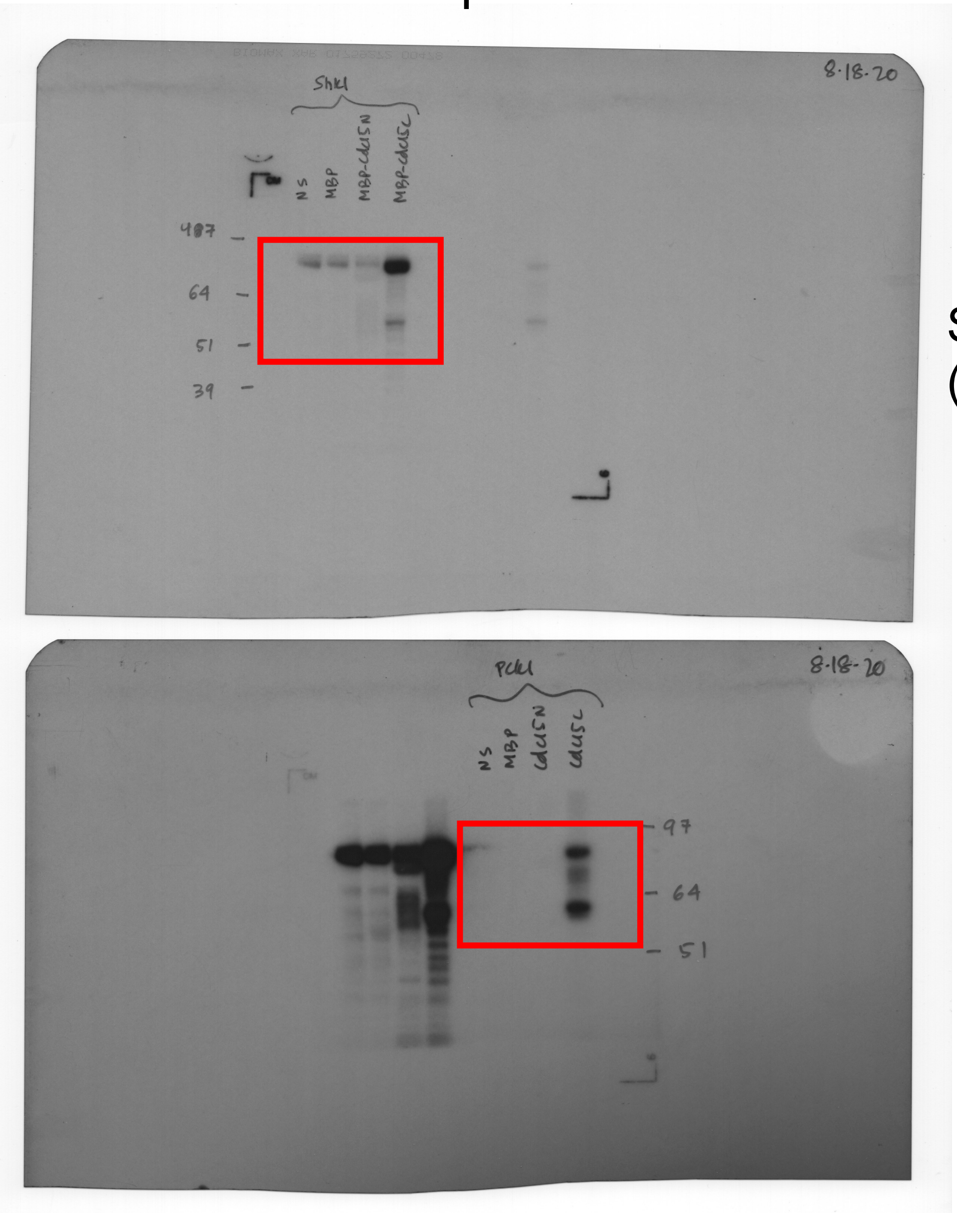


Shk1 (top)

Pck1 (bottom)

Coomassie Stain

Shk1 (left)

Pck1 (right)

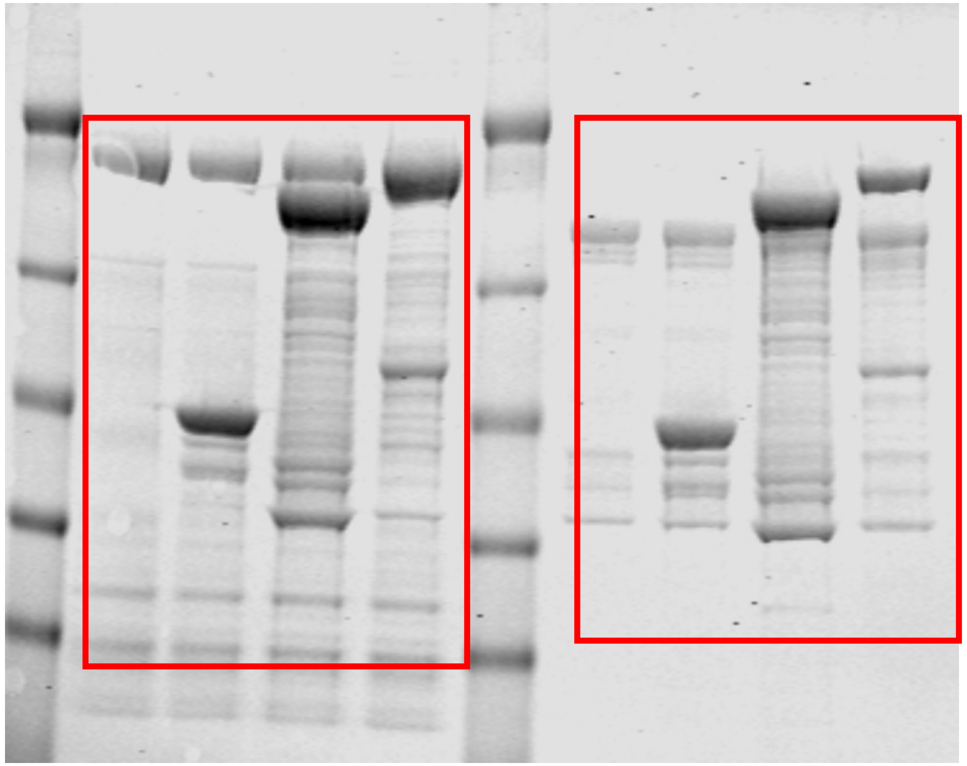

# Figure 1-figure supplement-1B (Kin1-left, Shk1-middle and Pck1-right)-Coomassie

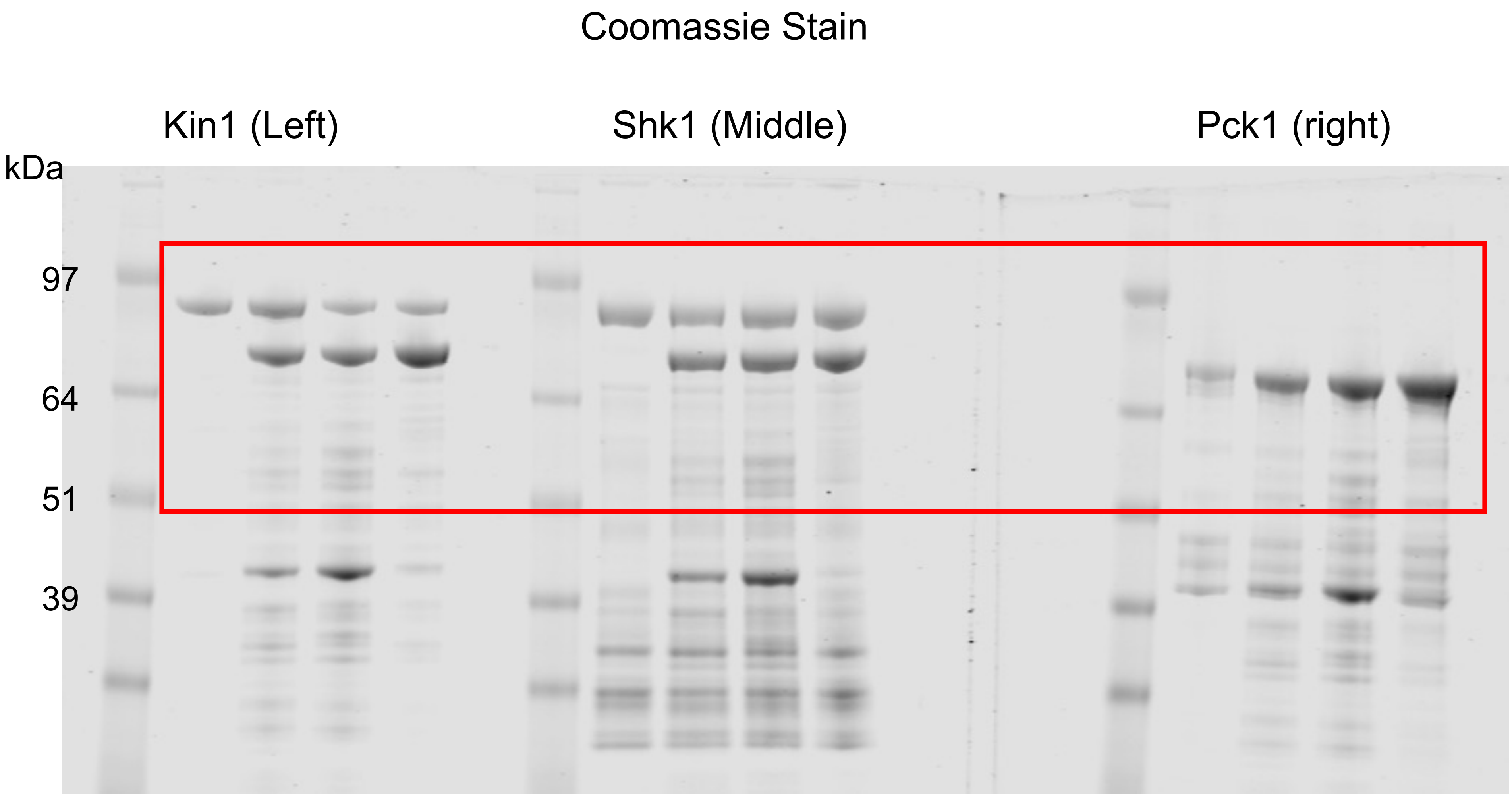

# Figure 1-figure supplement-1B (Kin1-left top, Shk1-right and Pck1-left bottom)-$^{32}$p

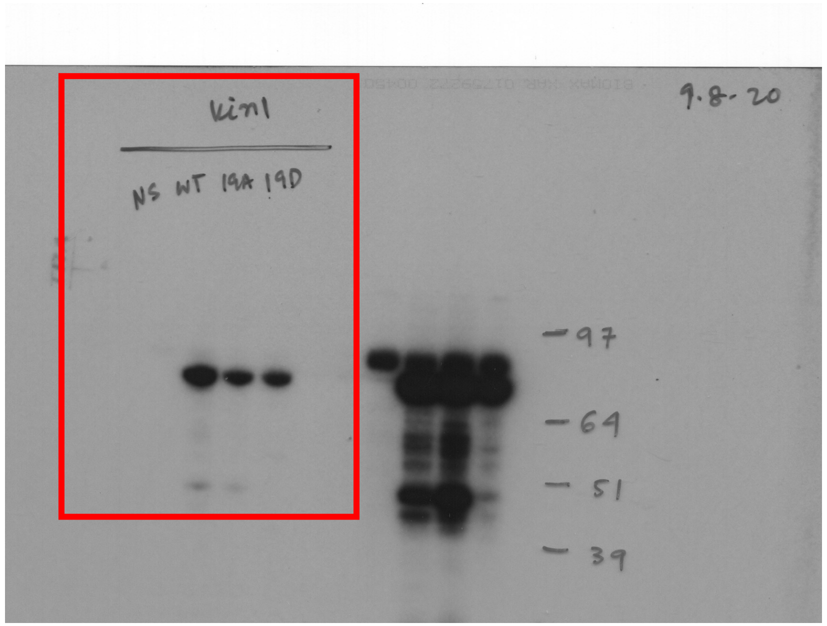


Kin1

$^{32}$p

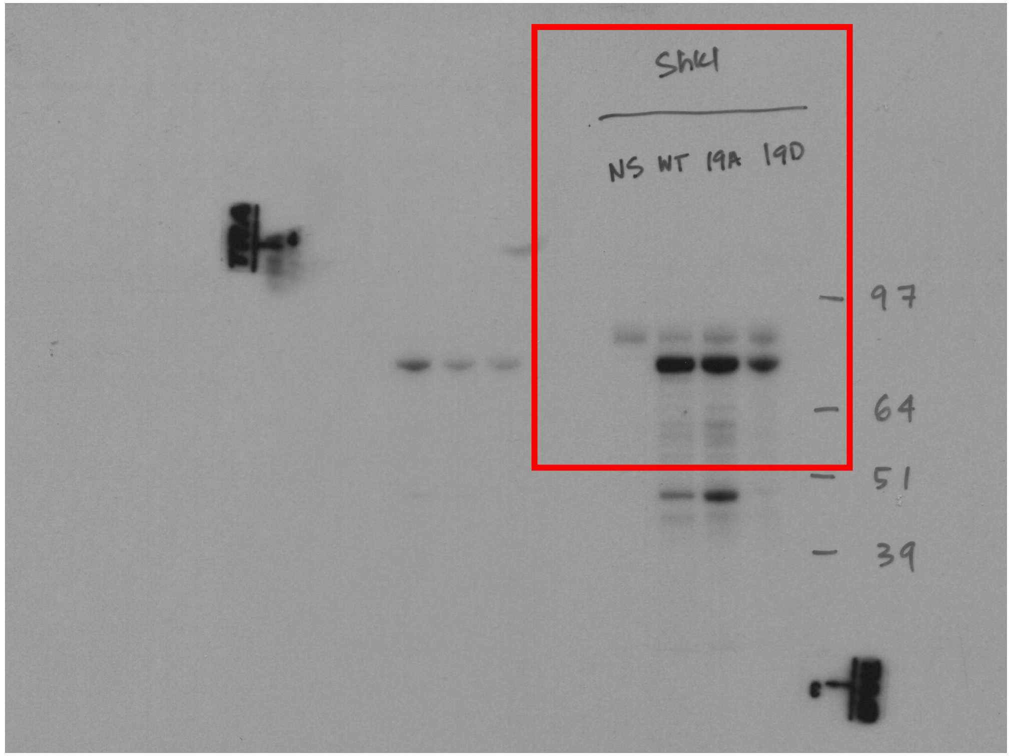


Shk1

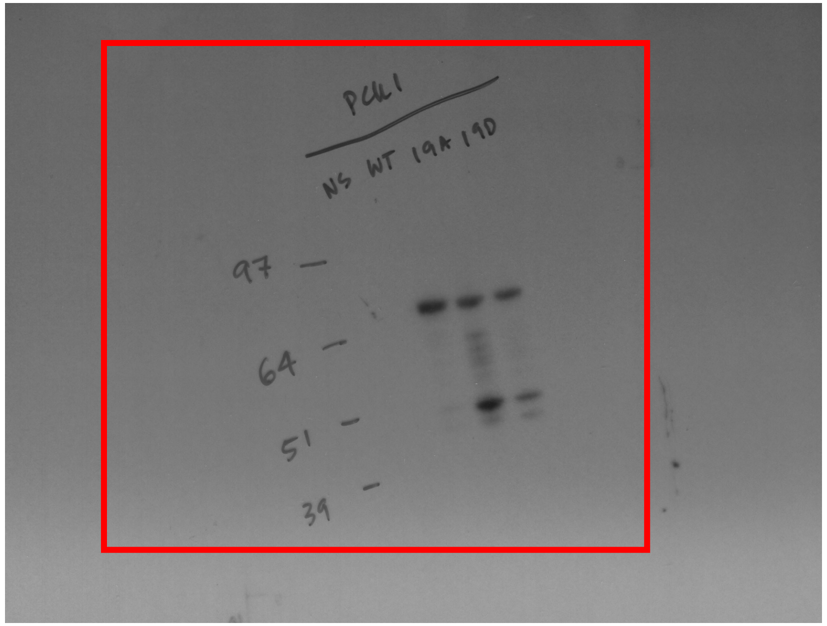


Pck1
