# Supplementary material for: Multiple polarity kinases inhibit phase separation of F-BAR protein Cdc15 and antagonize cytokinetic ring assembly in fission yeast
Source: eLife. 2023 Feb 7;12:e83062. doi: 10.7554/eLife.83062 (PMC9904764; doi:10.7554/eLife.83062)
Supplement: Figure 2—figure supplement 1—source data 1. [file elife-83062-fig2-figsupp1-data1.zip › Figure 1-figure supplement 1/Figure 1-supplemen 1A (Kin1)/Kin1-p32.pdf]

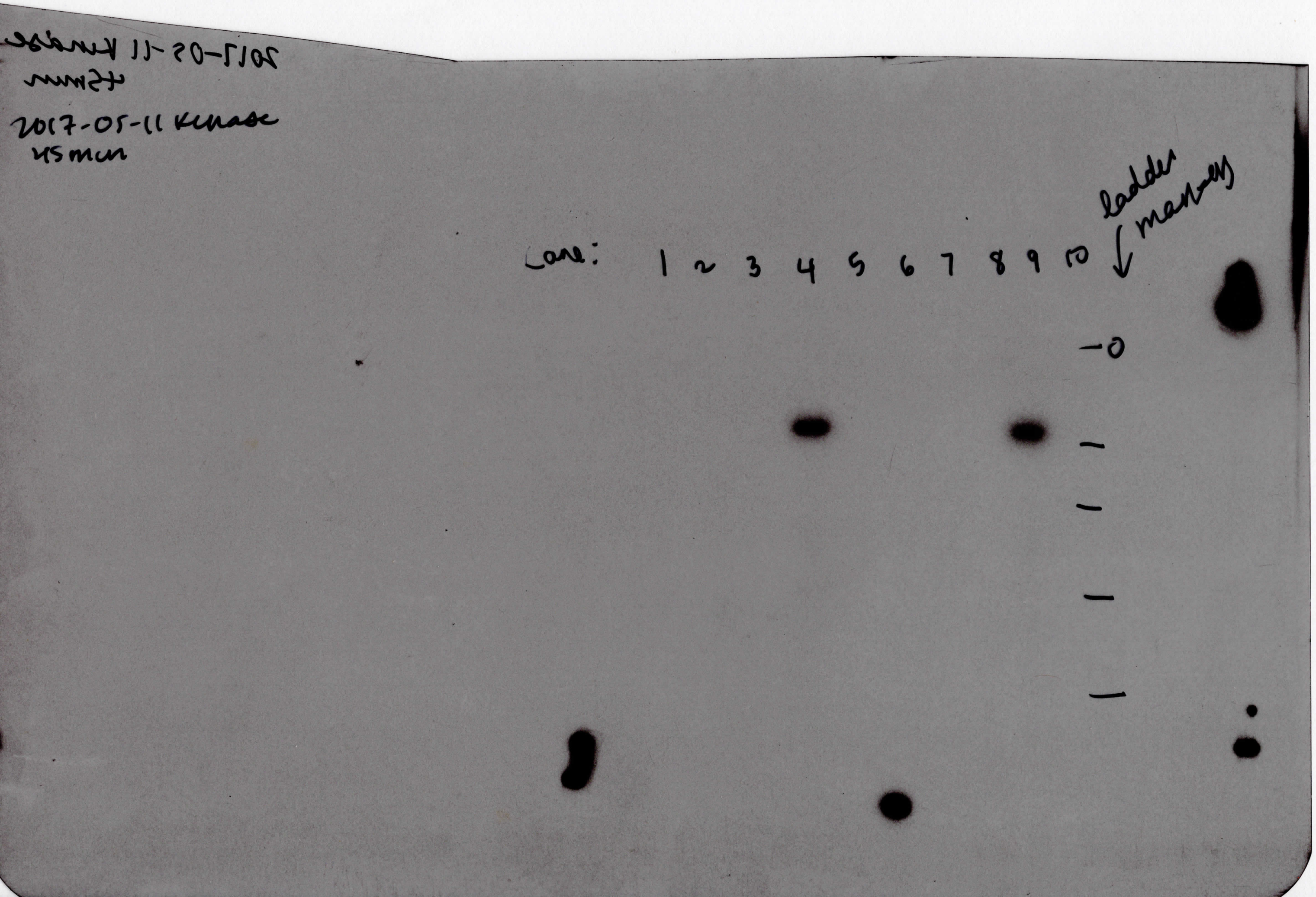
2017-05-11 Kinase
45min
Lane: 1 2 3 4 5 6 7 8 9 10
ladder markers
0
