# Supplementary material for: Multiple polarity kinases inhibit phase separation of F-BAR protein Cdc15 and antagonize cytokinetic ring assembly in fission yeast
Source: eLife. 2023 Feb 7;12:e83062. doi: 10.7554/eLife.83062 (PMC9904764; doi:10.7554/eLife.83062)

Figure 7-figure supplement 1A

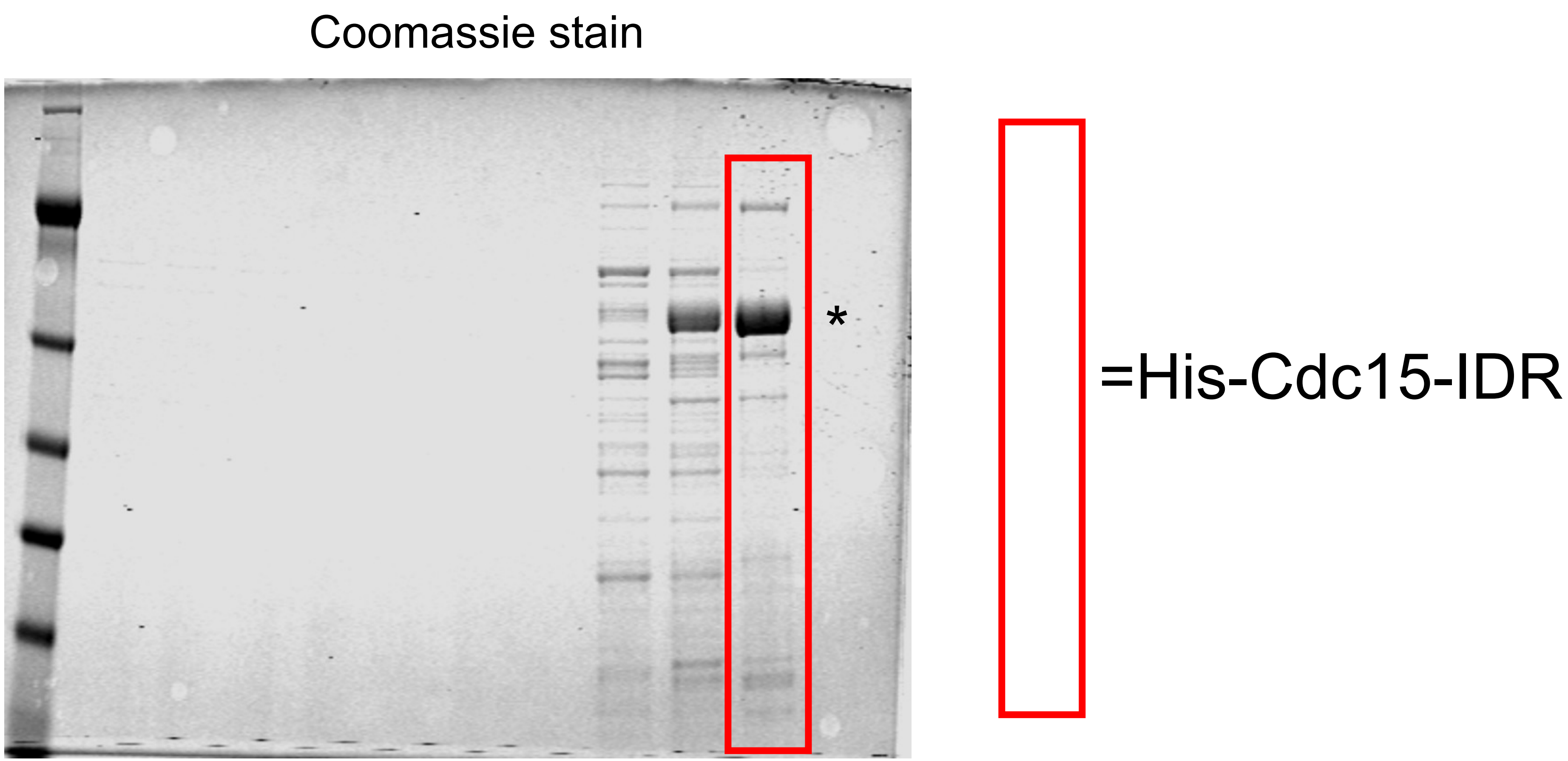

Figure 7-figure supplement 1B

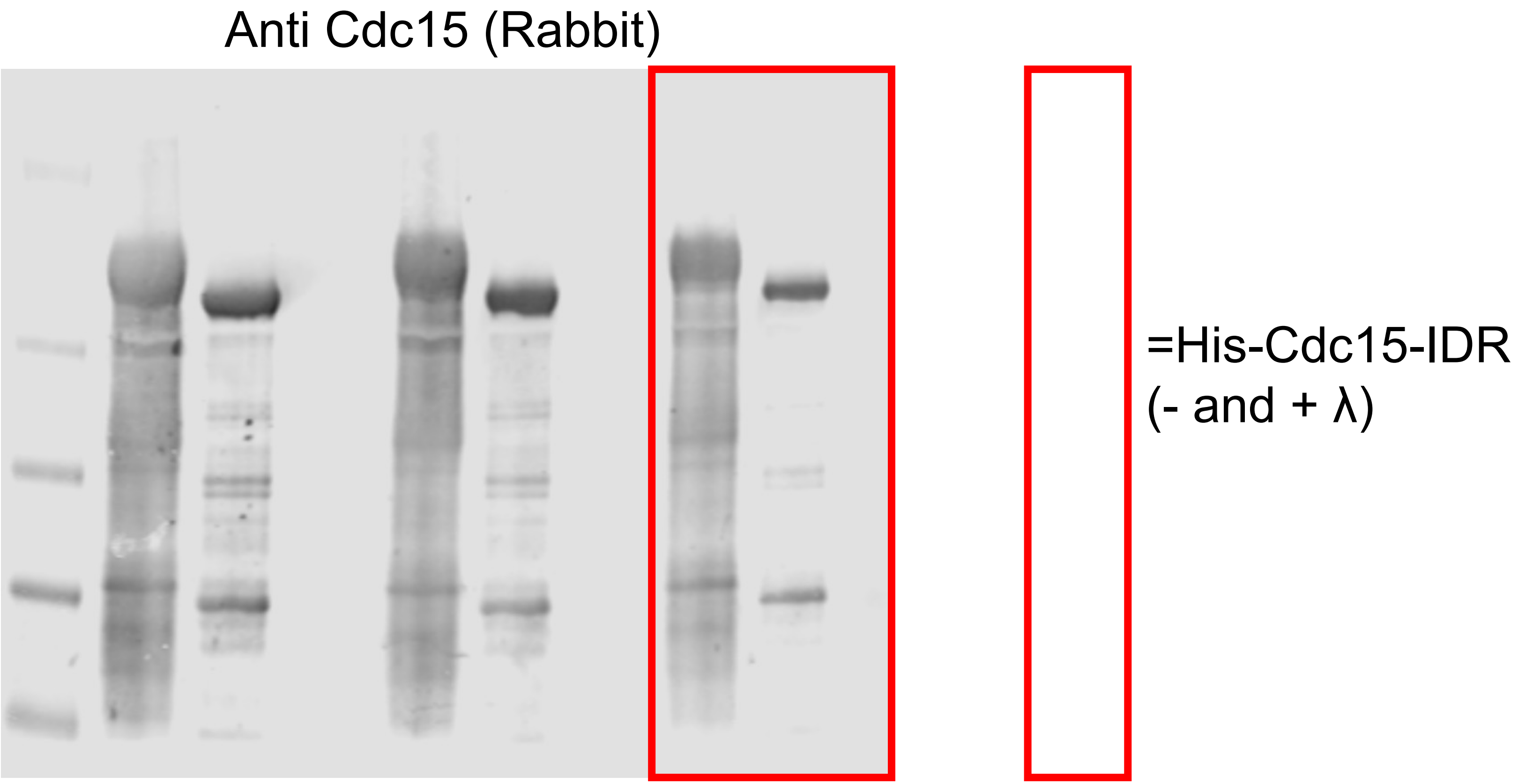

Supplement: Figure 7—figure supplement 1—source data 1. [file elife-83062-fig7-figsupp1-data1.zip › Figure 7-figure supplement 1/Figure 7-figure supplement 1.pdf]
