# Supplementary material for: Multiple polarity kinases inhibit phase separation of F-BAR protein Cdc15 and antagonize cytokinetic ring assembly in fission yeast
Source: eLife. 2023 Feb 7;12:e83062. doi: 10.7554/eLife.83062 (PMC9904764; doi:10.7554/eLife.83062)
Supplement: Figure 8—figure supplement 1—source data 1. [file elife-83062-fig8-figsupp1-data1.zip › Figure 8-figure supplement 1/Figure 8-figure supplement 1.pdf]

Figure 8-figure supplement 1A

*

Coomassie stain

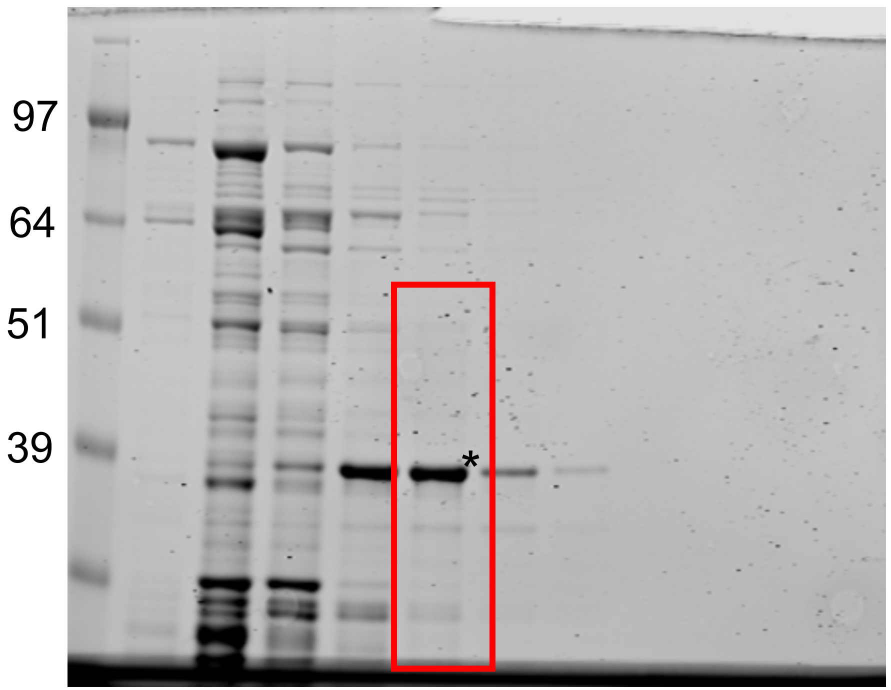

=Fic1-His$_6$

Figure 8-figure supplement 1B

*

Coomassie stain

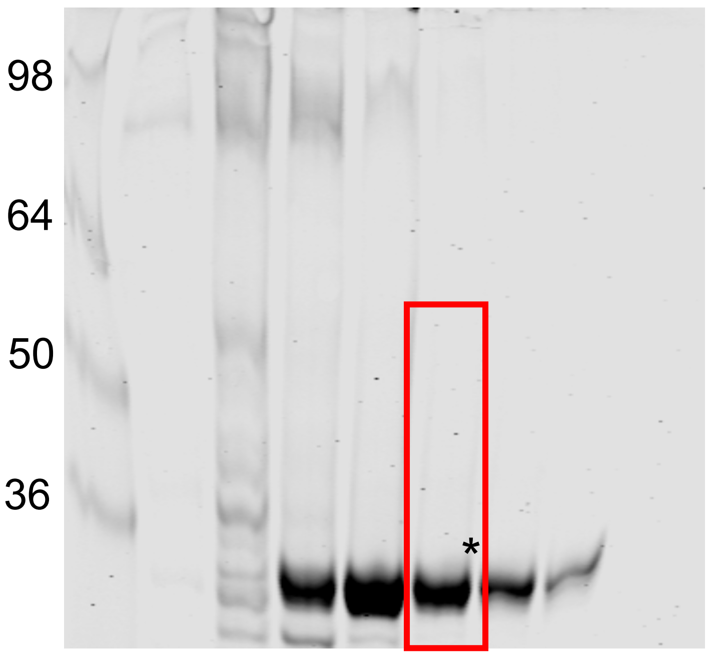

=His-mCherry

Figure 8-figure supplement 1C

*

Coomassie stain

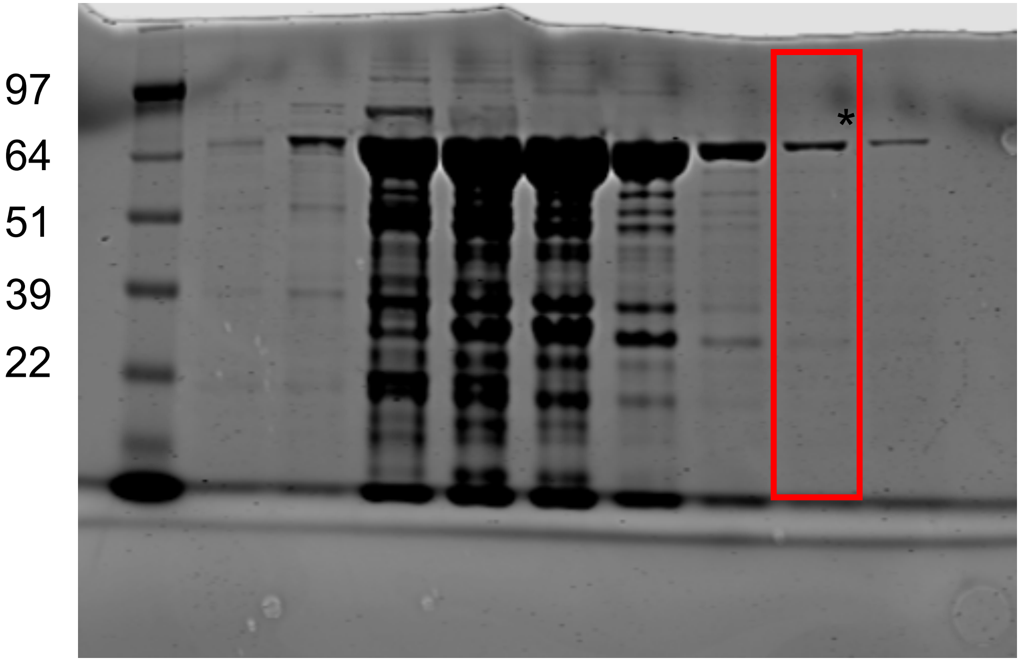

=His-Cdc15-IDR-SH3 (W903S)
