# Supplementary material for: Multiple polarity kinases inhibit phase separation of F-BAR protein Cdc15 and antagonize cytokinetic ring assembly in fission yeast
Source: eLife. 2023 Feb 7;12:e83062. doi: 10.7554/eLife.83062 (PMC9904764; doi:10.7554/eLife.83062)
Supplement: Supplementary file 1. [file elife-83062-supp1.docx]

**Supplemental Table 1. *S. pombe* strains used in this study**

| Strain number | Genotype | Source |
| --- | --- | --- |
| **Figure 1** |  |  |
| KGY246 | *ade6-M210 ura4-D18 leu1-32 h^-^* | Lab stock |
| KGY11562 | *cdc15-22A ade6-M210 ura4-D18 leu1-32 h^-^* | Lab stock |
| KGY2001-2 | *pom1^as1^-tdTomato:nat^R^ ade6-M210 ura4-D18 leu1-32 h^-^* | (Martin and Berthelot-Grosjean, 2009) |
| KGY1516-2 | *kin1^as1^-FLAG:kan^R^ ura4-294 leu1-32 ade6-704 h^+^* | This study |
| KGY2002-2 | *kin1^as1^-FLAG:kan^R^ pom1^as1^-tdTom:natR leu1-32 ade6-X ura4-X h^+^* | This study |
| KGY15733-2 | *kin1::ura4^+^ ade6-M210 ura4-D18 leu1-32 h^+^* | (Bimbo et al., 2005) |
| KGY11570-2 | *cdc15-22A kin1::ura4^+^ ade6-M21X ura4-D18 leu1-32h-* | This study |
| KGY19282 | *shk1::ClonNat^R^ shk1^as2^(M460A)-Hph^R^ leu1-32 ura4-D18 ade6-M210 h^−^* | (Cipak et al., 2011) |
| KGY1954-2 | *cdc15-22A shk1::ClonNat^R^ shk1-as^2^(M460A)-Hph^R^ ade6-M21X leu1-32 ura4-D18 h^-^* | This study |
| KGY6404 | *pck1::ura4^+^ ade6-M21X ura4-D18 leu1-32h^-^* | This study |
| KGY19615 | *cdc15-22A orb2-34 ade6-M21X leu1-32 ura4-D18 h^-^* | Lab stock |
| KGY1956-3 | *kin1^as1^:kan^R^ shk1::ClonNat^R^ shk1^as2^(M460A)-Hph^R^ pom1^as1^:nat^R^ ade6-M21X leu1-32 ura4-D18 h^-^* | This study |
| KGY1953-2 | *kin1^as1^:kan^R^ shk1::ClonNat^R^ shk1^as2^(M460A)-Hph^R^ ade6-M21X leu1-32 ura4-D18 h^-^* | This study |
| KGY1912-2 | *shk1::ClonNat^R^ shk1^as2^(M460A)-Hph^R^ pom1^as1^:nat^R^ ade6-M21X leu1-32 ura4-D18 h^-^* | This study |
| KGY7477 | *orb2-34 ade6-M21X leu1-32 ura4-D18 h^-^* | (Verde et al., 1995) |
| KGY1962-2 | *kin1^as1^:kan^R^ shk1::ClonNat^R^ shk1^as2^(M460A)-Hph^R^ pom1^as1^:nat^R^ pck1^as2^-HAx3:hyg^R^ ade6-M21X leu1-32 ura4-D18 h^-^* | This study |
| KGY526-2 | *pck1^as2^-FLAG:kan^R^ ade6-M21X leu1-32 ura4-D18 h^+^* | Lab stock |
| KGY19598 | *cdc15-22A pck1^as2^-HAx3:hyg^R^ ade6-M21X leu1-32 ura4-D18 h^-^* | This study |
| **Figure 3** |  |  |
| KGY246 | *ade6-M210 ura4-D18 leu1-32 h^-^* | Lab stock |
| KGY6012-2 | *cdc15-31A ade6-M210 ura4-D18 leu1-32 h^-^* | This study |
| KGY56 | *nda3-km311leu1-32h^+^* | Lab stock |
| KGY5099 | *cps1-191 leu1-32lys1-151ura4-D18h^+^* | Lab stock |
| **Figure 4** |  |  |
| KGY5365-2 | *mNG-cdc15 rlc1-mCherry:nat^R^ sid4-mCherry:hyg^R^ leu1-32 ura4-D18 ade6-M210 h^-^* | Lab stock |
| KGY5366-2 | *mNG-cdc15-31A rlc1-mCherry:nat^R^ sid4-mCherry:hyg^R^ leu1-32 ura4-D18 ade6-M210 h^-^* | This study |
| KGY6053-2 | *cdc15-31A rlc1-mNG:hyg^R^ sid4-mNG:Kan^R^ ade6-21x ura4-D18 leu1-32 h^+^* | This study |
| KGY19083 | *rlc1-mNeonGreen:hygR sid4-mNeonGreen:kan^R^ ade6-M21X ura4-d18 leu1-32 h^+^* | Lab stock |
| KGY1498-2 | *mNG-cdc15 ade6-M21X ura4-D18 leu1-32 h^-^* | Lab stock |
| KGY6011-2 | *mNG-Cdc15-31A ade6-21x ura4-D18 leu1-32 h^-^* | This study |
| **Figure 5** |  |  |
| KGY1498-2 | *mNG-cdc15 ade6-M21X ura4-D18 leu1-32 h^-^* | Lab stock |
| KGY4013-2 | *pom1^as1^-tdTomato:nat^R^ mNG-cdc15 ade6-M21X ura4-D18 leu1-32 h^-^* | This study |
| KGY16119 | *kin1^as1^-FLAG:kan^R^ mNG-cdc15 ade6-M21X ura4-D18 leu1-32 h^+^* | This study |
| KGY1753-3 | *shk1::ClonNat^R^ shk1^as2^(M460A)-Hph^R^ mNG-cdc15 ura4-294 leu1-32 ade6-M21X h^-^* | This study |
| KGY1766-3 | *pck1^as2^-FLAG:kan^R^ mNG-cdc15 ura4-294 leu1-32 ade6-M21X h^-^* | This study |
| KGY4110-2 | *pom1^as1^-tdTomato:nat^R^ kin1^as1^-FLAG:kan^R^ mNG-cdc15 ade6-M21X ura4-D18 leu1-32 h^-^* | This study |
| KGY4075-2 | *pom1^as1^-tdTomato:nat^R^ shk1::ClonNat^R^ shk1^as2^(M460A)-Hph^R^ mNG-cdc15 ade6-M21X ura4-D18 leu1-32 h^-^* | This study |
| KGY4097-2 | *pom1^as1^-tdTomato:nat^R^ pck1^as2^-FLAG:kan^R^mNG-cdc15 ade6-M21X ura4-D18 leu1-32 h^-^* | This study |
| KGY4132-2 | *kin1^as1^-FLAG:kan^R^ shk1::ClonNat^R^ shk1^as2^(M460A)-Hph^R^ mNG-cdc15 ade6-M21X ura4-D18 leu1-32 h^-^* | This study |
| KGY4151-2 | *kin1^as1^-FLAG:kan^R^ pck1^as2^-FLAG:kan^R^mNG-cdc15 ade6-M21X ura4-D18 leu1-32 h^-^* | This study |
| KGY4133-2 | *shk1::ClonNat^R^ shk1^as2^(M460A)-Hph^R^ pck1^as2^-FLAG:kan^R^mNG-cdc15 ade6-M21X ura4-D18 leu1-32 h^-^* | This study |
| KGY4149-2 | *pom1^as1^-tdTomato:nat^R^ kin1^as1^-FLAG:kan^R^ shk1::ClonNat^R^ shk1^as2^(M460A)-Hph^R^ mNG-cdc15 ade6-M21X ura4-D18 leu1-32 h^-^* | This study |
| KGY4148-2 | *pom1^as1^-tdTomato:nat^R^ shk1::ClonNat^R^ shk1^as2^(M460A)-Hph^R^pck1^as2^-FLAG:kan^R^ mNG-cdc15 ade6-M21X ura4-D18 leu1-32 h^-^* | This study |
| KGY4152-2 | *pom1^as1^-tdTomato:nat^R^ kin1^as1^-FLAG:kan^R^ pck1^as2^-FLAG:kan^R^mNG-cdc15 ade6-M21X ura4-D18 leu1-32 h^-^* | This study |
| KGY4153-2 | *kin1^as1^-FLAG:kan^R^ shk1::ClonNat^R^ shk1^as2^(M460A)-Hph^R^ pck1^as2^-FLAG:kan^R^mNG-cdc15 ade6-M21X ura4-D18 leu1-32 h^-^* | This study |
| KGY1183-3 | *mNG-Cdc15-1A ade6-21x ura4-D18 leu1-32 h^-^* | This study |
| KGY706-2 | *mNG-Cdc15-5A ade6-21x ura4-D18 leu1-32 h^-^* | This study |
| KGY6007-2 | *mNG-Cdc15-11A ade6-21x ura4-D18 leu1-32 h^-^* | This study |
| KGY19616 | *mNG-Cdc15-22A ade6-21x ura4-D18 leu1-32 h^-^* | Lab stock |
| KGY6011-2 | *mNG-Cdc15-31A ade6-21x ura4-D18 leu1-32 h^-^* | This study |
| KGY3019 | *cdc15-GFP::kan^R^ ade6-M210 ura4-D18 leu1-32 h^-^* | Lab stock |
| KGY9446 | *cdc15::cdc15(SP11A)-GFP:kan^R^ ade6-M210 ura4-D18 leu1-32 h^-^* | Lab stock |
| KGY9444 | *cdc15::cdc15(RXXS13A)-GFP:kan^R^ ade6-M210 ura4-D18 leu1-32 h^-^* | Lab stock |
| KGY8461 | *cdc15::cdc15(SP11+others-18A)-GFP:kan^R^ ade6-M210 ura4-D18 leu1-32 h^-^* | Lab stock |
| KGY10307 | *cdc15::cdc15(RXXS+SP+others27A)-GFP:kan^R^ ade6-M210 ura4-D18 leu1-32 h^+^* | Lab stock |
| **Figure 9** |  |  |
| KGY1498-2 | *mNG-cdc15 ade6-M21X ura4-D18 leu1-32 h^-^* | Lab stock |
| KGY19616 | *mNG-Cdc15-22A ade6-21x ura4-D18 leu1-32 h^-^* | Lab stock |
| KGY6011-2 | *mNG-Cdc15-31A ade6-21x ura4-D18 leu1-32 h^-^* | This study |
| KGY6058-2 | *cdc15-mCherry:Nat^R^ rlc1-mNG:hyg^R^ ade6-M210 ura4-D18 leu1-32 h^-^* | This study |
| KGY6061-2 | *cdc15-31A-mCherry:Nat^R^ rlc1-mNG:hyg^R^ ade6-M210 ura4-D18 leu1-32 h^-^* | This study |
| KGY5994-2 | *cdc15-mCherry:Nat^R^ fic1-mNG:Kan^R^ ade6-M21x ura4-D18 leu1-32 h-* | This study |
| KGY5993-2 | *cdc15-31A-mCherry:Nat^R^ fic1-mNG:Kan^R^ ade6-M21x ura4-D18 leu1-32 h-* | This study |
| **Figure 5-figure sup1** |  |  |
| KGY1498-2 | *mNG-cdc15 ade6-M21X ura4-D18 leu1-32 h^-^* | Lab stock |
| KGY4013-2 | *pom1^as1^-tdTomato:nat^R^ mNG-cdc15 ade6-M21X ura4-D18 leu1-32 h^-^* | This study |
| KGY16119 | *kin1^as1^-FLAG:kan^R^ mNG-cdc15 ade6-M21X ura4-D18 leu1-32 h^+^* | This study |
| KGY1753-3 | *shk1::ClonNat^R^ shk1^as2^(M460A)-Hph^R^ mNG-cdc15 ura4-294 leu1-32 ade6-M21X h^-^* | This study |
| KGY1766-3 | *pck1^as2^-FLAG:kan^R^ mNG-cdc15 ura4-294 leu1-32 ade6-M21X h^-^* | This study |
| KGY4110-2 | *pom1^as1^-tdTomato:nat^R^ kin1^as1^-FLAG:kan^R^ mNG-cdc15 ade6-M21X ura4-D18 leu1-32 h^-^* | This study |
| KGY4075-2 | *pom1^as1^-tdTomato:nat^R^ shk1::ClonNat^R^ shk1^as2^(M460A)-Hph^R^ mNG-cdc15 ade6-M21X ura4-D18 leu1-32 h^-^* | This study |
| KGY4097-2 | *pom1^as1^-tdTomato:nat^R^ pck1^as2^-FLAG:kan^R^mNG-cdc15 ade6-M21X ura4-D18 leu1-32 h^-^* | This study |
| KGY4132-2 | *kin1^as1^-FLAG:kan^R^ shk1::ClonNat^R^ shk1^as2^(M460A)-Hph^R^ mNG-cdc15 ade6-M21X ura4-D18 leu1-32 h^-^* | This study |
| KGY4151-2 | *kin1^as1^-FLAG:kan^R^ pck1^as2^-FLAG:kan^R^mNG-cdc15 ade6-M21X ura4-D18 leu1-32 h^-^* | This study |
| KGY4133-2 | *shk1::ClonNat^R^ shk1^as2^(M460A)-Hph^R^ pck1^as2^-FLAG:kan^R^mNG-cdc15 ade6-M21X ura4-D18 leu1-32 h^-^* | This study |
| KGY4149-2 | *pom1^as1^-tdTomato:nat^R^ kin1^as1^-FLAG:kan^R^ shk1::ClonNat^R^ shk1^as2^(M460A)-Hph^R^ mNG-cdc15 ade6-M21X ura4-D18 leu1-32 h^-^* | This study |
| KGY4148-2 | *pom1^as1^-tdTomato:nat^R^ shk1::ClonNat^R^ shk1^as2^(M460A)-Hph^R^pck1^as2^-FLAG:kan^R^ mNG-cdc15 ade6-M21X ura4-D18 leu1-32 h^-^* | This study |
| KGY4152-2 | *pom1^as1^-tdTomato:nat^R^ kin1^as1^-FLAG:kan^R^ pck1^as2^-FLAG:kan^R^mNG-cdc15 ade6-M21X ura4-D18 leu1-32 h^-^* | This study |
| KGY4153-2 | *kin1^as1^-FLAG:kan^R^ shk1::ClonNat^R^ shk1^as2^(M460A)-Hph^R^ pck1^as2^-FLAG:kan^R^mNG-cdc15 ade6-M21X ura4-D18 leu1-32 h^-^* | This study |
| KGY1498-2 | *mNG-cdc15 ade6-M21X ura4-D18 leu1-32 h^-^* | Lab stock |
| KGY1183-3 | *mNG-Cdc15-1A ade6-21x ura4-D18 leu1-32 h^-^* | This study |
| KGY706-2 | *mNG-Cdc15-5A ade6-21x ura4-D18 leu1-32 h^-^* | This study |
| KGY6007-2 | *mNG-Cdc15-11A ade6-21x ura4-D18 leu1-32 h^-^* | This study |
| KGY19616 | *mNG-Cdc15-22A ade6-21x ura4-D18 leu1-32 h^-^* | Lab stock |
| KGY6011-2 | *mNG-Cdc15-31A ade6-21x ura4-D18 leu1-32 h^-^* | This study |
| **Figure 5- figure sup 2** |  |  |
| KGY246 | *ade6-M210 ura4-D18 leu1-32 h^-^* | Lab stock |
| KGY2711 | *mid1::ura4^+^ ade6-M210 ura4-D18 leu1-32 h^+^* | Lab stock |
| KGY4951-2 | *mid1::ura4^+^ pom1-as1-tdTomato:nat^R^ ade6-M210 ura4-D18 leu1-32 h^+^* | Lab stock |
| KGY1516-2 | *kin1^as1^-FLAG:kan^R^ ura4-294 leu1-32 ade6-704 h^+^* | This study |
| KGY19282 | *shk1::ClonNat^R^ shk1^as2^(M460A)-Hph^R^ leu1-32 ura4-D18 ade6-M210 h^−^* | (Cipak et al., 2011) |
| KGY526-2 | *pck1^as2^-FLAG:kan^R^ ade6-M21X leu1-32 ura4-D18 h^+^* | Lab stock |
| KGY19781 | *mid1::ura4^+^ kin1^as1^-FLAG:kan^R^ ura4-294 leu1-32 ade6-704 h^+^* | This study |
| KGY19782 | *mid1::ura4^+^ pck1^as2^-FLAG:kan^R^ ade6-M21X leu1-32 ura4-D18 h^+^* | This study |
| KGY5911-2 | *mid1::ura4^+^ shk1::ClonNat^R^ shk1^as2^(M460A)-Hph^R^ leu1-32 ura4-D18 ade6-M210 h^-^* | This study |

**References**

Bimbo, A., Jia, Y., Poh, S.L., Karuturi, R.K., den Elzen, N., Peng, X., Zheng, L., O'Connell, M., Liu, E.T., Balasubramanian, M.K., and Liu, J. (2005). Systematic deletion analysis of fission yeast protein kinases. Eukaryot Cell *4*, 799-813. 10.1128/EC.4.4.799-813.2005.

Cipak, L., Zhang, C., Kovacikova, I., Rumpf, C., Miadokova, E., Shokat, K.M., and Gregan, J. (2011). Generation of a set of conditional analog-sensitive alleles of essential protein kinases in the fission yeast Schizosaccharomyces pombe. Cell Cycle *10*, 3527-3532. 10.4161/cc.10.20.17792.

Martin, S.G., and Berthelot-Grosjean, M. (2009). Polar gradients of the DYRK-family kinase Pom1 couple cell length with the cell cycle. Nature *459*, 852-856. 10.1038/nature08054.

Verde, F., Mata, J., and Nurse, P. (1995). Fission yeast cell morphogenesis: identification of new genes and analysis of their role during the cell cycle. J Cell Biol *131*, 1529-1538. 10.1083/jcb.131.6.1529.
